# Supplementary material for: Giant chromosomes of a tiny plant—the complete telomere-to-telomere genome assembly of the simple thalloid liverwort Apopellia endiviifolia (Jungermanniopsida, Marchantiophyta)
Source: Gigascience. 2025 Nov 29;15:giaf145. doi: 10.1093/gigascience/giaf145 (PMC12885004; doi:10.1093/gigascience/giaf145)
Supplement: giaf145_Supplemental_Files [file giaf145_supplemental_files.zip › GIGA-D-25-00252-34-36.pdf]

Supplementary Table S1. Tandem centromeric repeats of *in silico* validated centromeric regions in *A. endiviifolia* assembly. Centromeres in chromosomes 1-3 and 6-9 were identified by the Centier program, and centromeres in chromosomes 4 and 5 were identified by the quarTeT program. All centromeres were validated using a Pore-C interaction heatmap.

Chromosomes	Tandem centromeric repeats
Ch1	CCCTAAAATAGTTTGCTGCTTCTTATGACATTCATTGTTTTGATCAACATAGC<br>AACACGAGAGAAGGTTTATAATGAATCCTGCCA
Ch2	AATTGAAATCATAAATGAAAACCAG
Ch3	ACAACAACATGAGAGAAGGTTTATAATGAATCCTGCCACCCTAAAATCATTT<br>GCTGCTTCTTATGAAATCATTGTTTTGATCGAC
Ch4	GAGGAGTGGTAACCTGAGTATGCAGGTCACCAGTGACCTAAACTATAGGTCA<br>CTACCTACTTCACACCTACCTCAAGTCATATCGCCTACCTCAAACCACATGAA<br>CCATGTGGTTCCTAACACATGCATCATGTGTTAGTACTTAGTACTAACTAATC<br>ATTTAGGCTAAGCTAGAGCTTGGAGTATTAACA
Ch5	TCACCGATGACCTGATCTATAGGTCACTACCTACTTCACACCTACCTCAAGTC<br>ATATGACCTACCTCAAACCTCATGAACCATGTGGTTCCTAACACATACATCAT<br>GTGTTAGTACTTAGTCCTAATTAATCATTTAGGCTAAGCTAGAACTTGGAGTA<br>TTAACAGTTTCTTCCACCAGAACTCGGAGGGTGA
Ch6	GGTTGTGAGCCACTTTTAACCGCGCGGAGTTGATCCCGGGTACAAATTTCCCC<br>AGAAGTATATATGAGGGGGAGGGGTTCGGCGTCTCGGTCCAGGCGCACGGA<br>ACGGACCGGCCTCGGGTCTCTTCGGTTGGTCCGGCTCGGTG
Ch7	AAATCATTTGCTGCTTCTTATGAAATCATTGTTTTGATCGACACAGCACCATG<br>AGAGAAGGTTTATAATGAATCCTGTCACCCTAAAATCATTTGCTGCTTCTTAT<br>GAAATCATTGTTTTGATCGACACAGCACCATGAGAGAAGGTTTATAATGAAT<br>CCTGTCACCCTAAAATCATTTGCTGCTTCTTATGAAATCATTGTTTTGATCGAC<br>ACAGCACCATGAGAGAAGGTTTATAATGAATCCTGTCACCCTA
Ch8	CGGGTTGTGAGCCACTCTTAACCGCGCGGAGTTGATCCCGGGTACAAATTTCC<br>CCAGAAGTATATATGAGGGGGAGGCGTTCGGCGTCTCGGTCCAGGCGCACGG<br>AACGGACCGGCCTCGGGTCTCGGCTTGTGCTCGGTTGAGG
Ch9	TCTCTCTCTCTCTCTCTCTCTCTCTCTCCCTGCCATCGCATCTCGAGCTGATCA<br>GAGCTCGACCTCGTCTTCTCTCTCTCTCTCTCTCTCTCTCTCTCTCTCTCTGC<br>CATCGCATTTCGAGCTGATCAGAGTTCGACCTCGTCCT

Supplementary figures

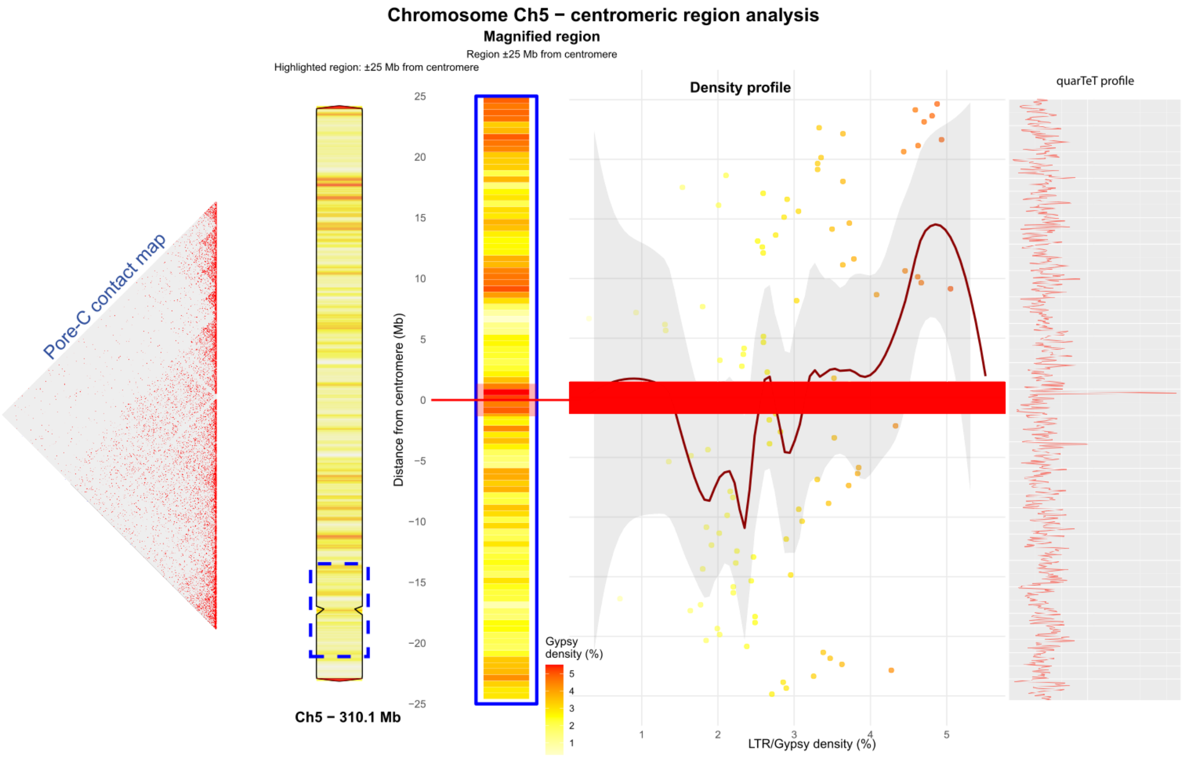

Figure S1. Detailed examination of the predicted centromere of chromosome 5 – Analysis of *Gypsy* retrotransposon distribution and chromatin interactions across chromosome 5. In the density profile, each dot represents a single genomic window, plotting its LTR/Gypsy density (X-axis) against its chromosomal position (Y-axis). The solid dark red line shows the overall trend in density, calculated using a LOESS regression, while the surrounding light grey shaded area indicates the 95% confidence interval for this trend. The thick red horizontal bar marks the position of the centromere across the center of the magnified plots.

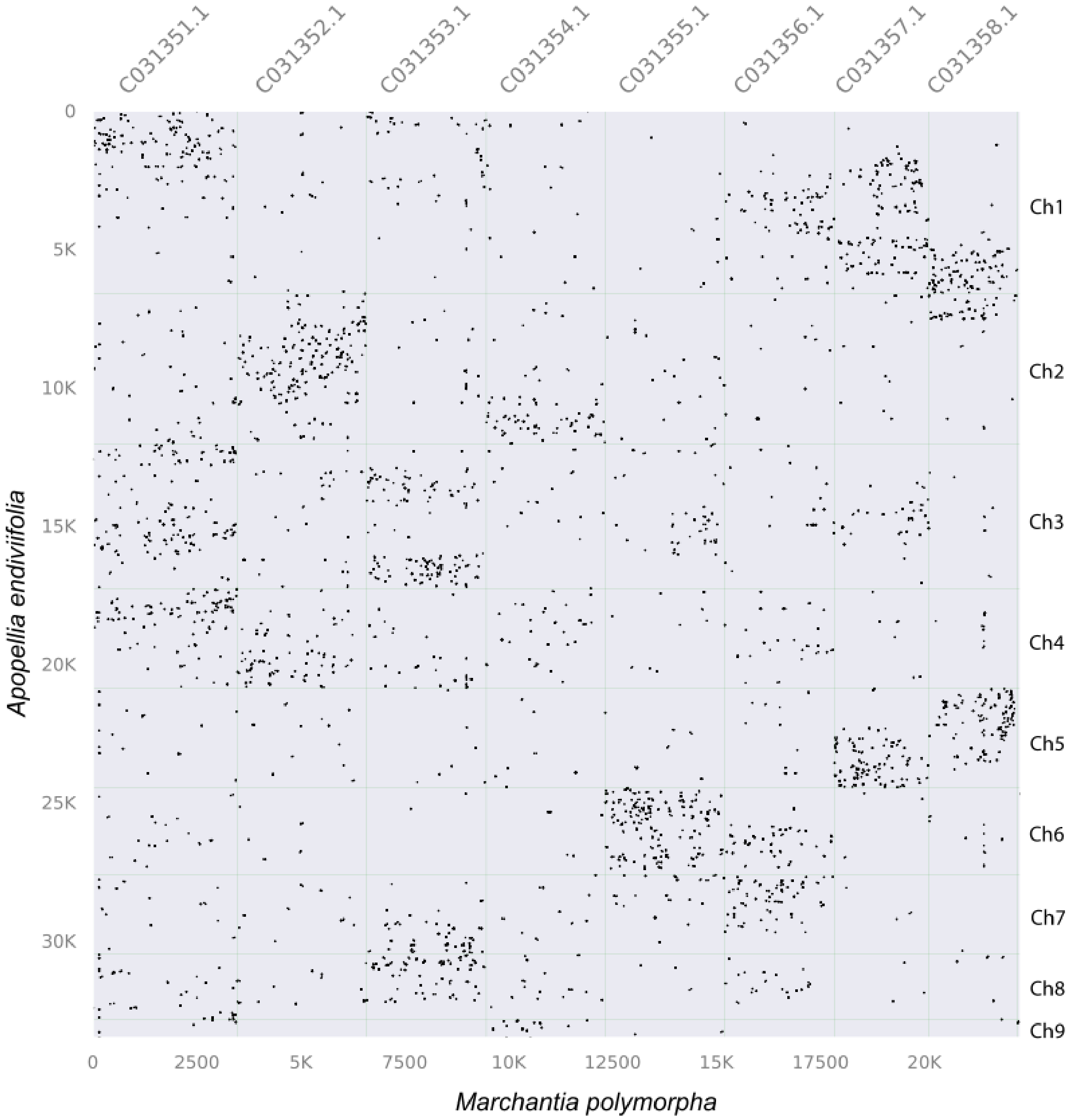

Figure S2. Synteny dot plot comparing the genomes of *M. polymorpha* and *A. endiviifolia*.
