# Supplementary material for: Giant chromosomes of a tiny plant—the complete telomere-to-telomere genome assembly of the simple thalloid liverwort Apopellia endiviifolia (Jungermanniopsida, Marchantiophyta)
Source: Gigascience. 2025 Nov 29;15:giaf145. doi: 10.1093/gigascience/giaf145 (PMC12885004; doi:10.1093/gigascience/giaf145)
Supplement: giaf145_GIGA-D-25-00252_Revision_1 [file giaf145_giga-d-25-00252_revision_1.pdf]

GigaScience

Giant chromosomes of a tiny plant - the complete telomere-to-telomere genome assembly of the simple thalloid liverwort Apopellia endiviifolia (Jungermanniopsida, Marchantiophyta)

--Manuscript Draft--

**Manuscript Number:**	GIGA-D-25-00252R1
**Full Title:**	Giant chromosomes of a tiny plant - the complete telomere-to-telomere genome assembly of the simple thalloid liverwort Apopellia endiviifolia (Jungermanniopsida, Marchantiophyta)
**Article Type:**	Data Note
**Funding Information:**	Narodowe Centrum Nauki (2020/39/B/NZ8/02504) — Prof. dr hab. Jakub Sawicki
**Abstract:**	Background<br>The liverwort A. endiviifolia, a dioicous, simple thalloid species, is notable for its cryptic diversity, habitat adaptability, genomic innovation, and represents a clade that is a sister to all other Jungermanniopsida. These features make A. endiviifolia an essential model for exploring speciation mechanisms and the evolution of genome structures within liverworts.<br>Findings<br>We present the genome assembly of haploid A. endiviifolia isolate with a total size of 2,914,960,273 bp and an N50 of 468,157,909 bp, demonstrating high completeness (99.2% BUSCO) and a high consensus quality (QV 47.6). The assembly consisted of nine chromosomes, which included validated 18 telomeres and nine centromeres (ranging from 1.9 to 5 Mbp in length). RNA-seq-based annotation identified 34,615 genes, predominantly protein-coding. The TEs comprised 12.16% LTR elements and 57 Helitrons. Among the retroelements, the Copia and Gypsy superfamilies comprised 8.94% and 2.95% of the genome, respectively. The Ty3/Gypsy superfamily was found to be significantly enriched in centromeric regions. The average GC content ranged from 38.8% to 39.6%, with gene density varied between a value 5.52 and 9.78 genes per 500 kbp. Synteny analysis of related liverwort species has revealed complex chromosomal relationships, indicating extensive genome rearrangements among species.<br>Conclusions<br><br>This study provides the first high-quality reference genome assembly of the haploid liverwort A. endiviifolia. Assembly and annotation offers valuable resources for investigating liverwort evolution, centromere biology, and genome expansion in simple thalloid liverworts.
**Corresponding Author:**	Joanna Szablińska-Piernik<br>University of Warmia and Mazury in Olsztyn: Uniwersytet Warminsko-Mazurski w Olsztynie<br>Olsztyn, POLAND
**Corresponding Author Secondary Information:**	
**Corresponding Author's Institution:**	University of Warmia and Mazury in Olsztyn: Uniwersytet Warminsko-Mazurski w Olsztynie
**Corresponding Author's Secondary Institution:**	
**First Author:**	Joanna Szablińska-Piernik
**First Author Secondary Information:**	
**Order of Authors:**	Joanna Szablińska-Piernik
	Paweł Sulima
	Jakub Sawicki

Powered by Editorial Manager® and ProduXion Manager® from Aries Systems Corporation

Order of Authors Secondary Information:	
Response to Reviewers:	We thank the Editor and Reviewers for all their valuable comments that helped us improve our manuscript. Below are detailed answers to each question or comment raised in the reviews.<br><br>Responses to Editor comments:<br><br>1. All web links and URLs should be given a reference number and included in the reference list rather than within the text of the manuscript. Please remove the URLs, cite them as reference and adjust the order of the reference accordingly. If you are using a reference manager then it's much easier for you to fix this.<br><br>Answer: All links and URLs have been added to the reference list.<br><br>2. In the methods section it would be useful if you add RRID details to some of the software tools and sequencers. These needed to be listed after resources in brackets. If you are citing papers for these resources, the RRID do not replace these, and both should be included. This can be included in the methods section of the paper similar to the RRIDs included here:<br>DNBSEQ-T7 (RRID:SCR_017981); PacBio Sequel II System (RRID:SCR_017990); PLINK (RRID:SCR_001757)<br><br>Answer: RRID details have been added to all software tools and sequencers for which they were found.<br><br>Responses to Reviewer #1:<br>Reviewer #1: Review to the manuscript entitled: "Giant chromosomes of tiny plant - the complete telomere-1 to-telomere genome assembly of the simple thalloid liverwort Apopellia endiviifolia (Jungermanniopsida,Marchantiophyta)"<br>Overall quality<br>The manuscript by Joanna Szablińska-Piernik et al. reports the annotated genome of the liverwort Apopellia endiviifolia. The manuscript properly follows the formal requirements of genome reports published in Gigascience. It contains a sufficiently detailed introduction a well-structured result, a method and a short discussion section. The manuscript is relatively easy to read but its text would benefit from some editing and streamlining. Interpretation of the data is aided by figures and tables as well as supplementary files. Assembly and annotation files are provided and available on Gigascience`s ftp site. Figures and tables are informative with some errors that must be corrected prior publishing. The methods section needs also further improvement as some analyses are not sufficiently described in detail. The A. endiviifolia genome is of great importance because of its phylogenetic position (first simple thalloid genome sequenced) as well as its large size which contrasts with currently sequenced liverwort genomes.<br>Furthermore, A. endiviiifolia has been already used in the past as a model to study cytology, molecular biology, speciation as well as physiology of liverworts. This genome sequence will make it possible to develop this species into a proper model system within liverworts. Therefore, the manuscript provides a great contribution to the field, and the liverwort community will benefit from the availability of the chromosomal-scale assembly of A. endiviifolia.<br><br>Answer: We sincerely appreciate your detailed, thorough, and constructive review. We have carefully considered all your comments and suggestions, and have revised the manuscript accordingly to enhance its overall quality.<br><br>Nevertheless, the manuscript needs heavy editing to be publishable. My general concerns are the following:<br>Intro: The intro and the results section refer to liverworts as well as to Apopellia as a basal linage. This term should not be used as lineages are not basal or derived and their position is only relative and depends on the way (top, bottom) the phylogenetic tree was drawn (which is in itself an arbitrary decision). Please, revise these statements and rather refer to the age of the split between Pelliaceae and other liverworts. A similar statement must be revised claiming that liverworts are sister to all land plants as well as that they retained more ancestral characters than vascular plants.

Powered by Editorial Manager® and ProduXion Manager® from Aries Systems Corporation

Answer: Thank you for this important clarification regarding the terminology and phylogenetic interpretation. We have revised the Background and Results sections accordingly, avoiding phrases ‘basal lineage’ and ‘sister to all land plants’ and a clear statement about 'ancestral traits'.

Methods: for many of the software codes used the version is not reported. This is necessary to make the analyses repeatable. Also, some analyses lack sufficient details that must be further supplemented.

Answer: Thank you for pointing this out. We have now included the specific version numbers for all software tools used throughout the study to ensure full reproducibility of our analyses.

Results: One interesting part of this manuscript is the discovery of centromeres that appear to be different from what has been described for other liverworts sequenced so far. While it is tempting to believe that centromere structure of Apopellia is unique within liverworts, I think the interpretation must mention that centromeres have been only in silico predicted which represents a good but not yet fully verified hypothesis. Furthermore, the plots showing detailed genomic features of the centromeric regions, the tandem and nested putative centromeric repeats are not shown. I do think that this information would help the reader to interpret the data and vet the validity of the predicted centromeres.

Answer: Your comment has been included in the 're-use potential' section. Additionally, tandem centromeric repeats are provided in the Supplementary Table S1.

Detailed comments to the authors:
L1: "a tiny plant" instead of "tiny plant"
L19: it is "dioicous" and not "dioecious", also remove "and" after dioicous. "dioicous" is the term used for haploid plants.
L20: there is no "basal phylogenetic position". You could write "represents the deepest split within Jungemanniopsida" or "in a clade that is sister to all other Jungemanniopsida". Please, revise.
L22: What is "genomic structure", I would rather say "genome evolution" or "genome structure".
L24: write rather "haploid A. endiviifolia isolate"
L26 What does accuracy refer to ?
L27 rather "18 validated Telomeres"
L29 LTR rather than LTRs
L32: Gene density needs a unit: number of genes / 1kb or so. Please, revise.
L34: rather "extensive rearrangements among species"

Answer: All errors and typos mentioned above have been corrected in accordance with the comments.

L41 There is no "early divergent" rephrase it. Also, it would be better to say that they are part of bryophytes, but it forms a monophyletic clade with mosses. This is what the current phylogenetic data suggest.
L43-44: It is not sister to all other land plants. Revise. The best supported phylogenetic hypothesis resolves vascular plants and bryophytes within land plants each as monophyletic lineages. Within bryophytes, liverworts are sister to mosses and this clade is further sister to hornworts.
L46: again, it is not clear whether their features are retained or secondarily reduced. Currently the reduction hypothesis is preferred.

Answer: Your comments have been taken into account and the beginning of the introduction has been edited as follows: ‘Liverworts (Marchantiophyta) represent one of the earliest terrestrial plants, with fossil evidence indicating their emergence in the Middle Ordovician period approximately 419–447 million years ago [1]. They occupy a pivotal position in plant evolutionary history as members of the monophyletic bryophyte lineage, which, along with vascular plants, form two distinct monophyletic groups within terrestrial plants. Within bryophytes, liverworts are sister to mosses, forming the Setaphyta clade, which in turn is sister to hornworts. This phylogenetic framework is

Powered by Editorial Manager® and ProduXion Manager® from Aries Systems Corporation

supported by recent genomic analyses that affirm the monophyly of bryophytes and clarify the evolutionary relationships among early land plants [2]. Liverworts, along with other bryophytes such as mosses and hornworts, exhibit a suite of morphological and physiological features that shed light on plant adaptation to terrestrial environments, although it remains debated whether these features represent ancestral traits or are the result of secondary reductions, with recent evidence favoring the latter [3]. This group is often characterized by structural simplicity, a predominantly haploid life cycle, and a slow rate of molecular evolution, which corresponds to gradual morphological diversification [4].'

L47-50 I miss the citations referring to the slow evolutionary rates mentioned.

Answer: The citation has been completed: Linde AM, Singh S, Bowman JL, Eklund M, Cronberg N, Lagercrantz U. Genome Evolution in Plants: Complex Thalloid Liverworts (Marchantiopsida). Genome Biol Evol. Oxford University Press; 2023; doi: 10.1093/GBE/EVAD014.

L57 there is no "basal lineage", revise.

Answer: This has been removed.

L84 approximately, does this refer to the mean value?

Answer: Yes, this refers to the average value, 'approximately' has been removed.

L134: In vitro cultures are not "conducted" rather "established".

Answer: This has been corrected.

L140: Explain the meaning of culture sterility of 95%.

Answer: The results of culture sterility were given here to demonstrate the high effectiveness of the sterilization method, which we have used. Only sterile plants were used in the studies.

L169: I think it is protein digestion and not degradation.

Answer: The original ONT protocol (https://nanoporetech.com/document/extraction-method/plant-pore-c) uses the word 'degradation' and we agree that it is degradation rather than digestion.

L189 Please, report concentration of proteinase K and RNase used.

Answer: This information has been supplemented: '100 µl proteinase K (20 µg/µL) and 3 µl RNase A (100 µg/µl)'.

L180 please report version of minknow used.

Answer: This information has been supplemented: 'v24.11.10'.

L189: please, explain why two different library preps. were used.

Answer: The entire paragraph on RNA-seq has been edited and described in more detail as follows: Total RNA for short read procedure was extracted from four samples of male A. endiviifolia: land (i) and water (ii) form, antheridia (iii) and surrounding thallus (iv). This extraction was conducted using the RNA Plant Mini Spin Kit (Qiagen) according to the manufacturer's protocol. Short-read RNA-seq libraries were prepared using two distinct protocols. For RNA extracted from the water and land form of A. endiviifolia, libraries were constructed using the TruSeq Stranded Total RNA Library Prep Kit (Illumina) using the Ribo-Zero rRNA option, whereas for antheridia and surrounding thallus, which amount of extracted RNA was insufficient for TruSeq, libraries were prepared using the QIAseq FX Single Cell RNA Library Kit (Qiagen). All libraries were sequenced on an Illumina NovaSeq 6000 platform (Macrogen, Inc., Seoul, South Korea) in 2 × 150 pair-end mode. Raw reads were deposited in

BioProject PRJNA1279829 and BioSample SAMN49506713.

L190: sequencing, how, 150 bp paired-end? Stranded? please, explain.

Answer: This information has been supplemented ‘in 2x150 pair-end mode’.

L215: You say SAMBA gap-closed assembly was…. Please, let us know the parameters used for the SAMBA gap-closing procedure.

Answer: The following information has been added: ‘Samba scaffolders for gap-closing were run with default option with exception of -m parameter (minimum matching length) set to 9000 as recommended for large plant genomes [37].’

L218: citation of dorado is missing.

Answer: The citation has been completed: https://github.com/nanoporetech/dorado.

L223: I would also report results for the embryophyte ODB data set.
Answer: we added embryophyte OBD10 BUSCO results to the Table 1
L233: Could you explain why did you prefer EGAPx over other genome annotation pipelines? For instance, EGAPx only uses a restricted set of plant proteins. Especially bryophyte proteins are missing.

Answer: In our internal testing EGAPx generates more accurate annotation than BREAKER2, which was used in our previous studies [Levins et al. 2025 (Levins J, Paukszto Ł, Krawczyk K, Maździarz M, Arch BC, Cargill DC, et al.. Evolution of sexual systems and regressive evolution in Riccia. New Phytologist. John Wiley & Sons, Ltd; 2025; doi: 10.1111/NPH.20454.), Krawczyk et al. 2025 (Krawczyk K, Szablińska-Piernik J, Paukszto Ł, Maździarz M, Sulima P, Przyborowski JA, et al.. Chromosome-scale telomere to telomere genome assembly of common crystalwort (Riccia sorocarpa Bisch.). Scientific Data 2025 12:1. Nature Publishing Group; 2025; doi: 10.1038/s41597-025-04373-6.)]. Moreover, EGAPx enables the use of a custom protein set (in this study we used a protein set from Marchantia polymorpha (Tak-1/2 v7.1).

L234: Was this a male isolate as the RNAseq used was from a male?

Answer: Yes, all isolates used in this study were from males.

L247: which edta version was used? Some versions have issues, so it is important to report the version.

Answer: This information has been supplemented: ‘v2.2.2.’

L256: For each software please describe the version and options used. If it was run using default options, please, state this.

Answer: The information for each software version and option has been supplemented.

L277 indicate M poly genome version.

Answer: Marchantia polymorpha genome version is ‘Tak-1/2 v7.1’

L278 CHEK GENBANK accession

Answer: Yes, we made a mistake here, the accession numbers have been corrected as follows: ‘The genomic sequences (FASTA) and gene annotation files (GFF3) for M. polymorpha (Tak-1/2 v7.1, GCA_037833965.1) and A. endiviifolia (JBRAUX000000000) were obtained from GenBank.’

L312: gggenomes package version

Answer: This information has been supplemented: ‘v1.0.1’.

Powered by Editorial Manager® and ProduXion Manager® from Aries Systems Corporation

L339_341: "differring evolutionayr pressures"? could you explain this a bit more in details?

Answer: Thank you for pointing this out. We agree that the phrase “differing evolutionary pressures” was rather unclear, and we have revised the text to provide a clearer explanation: ‘Additionally, the observed variation in genome size, particularly in the large genomes of some Jungermanniopsida, may reflect lineage-specific differences in genome dynamics, such as variation in transposable element activity, gene duplication, and structural rearrangements.’

L466: Close the bracket.

Answer: This has been corrected.

References: formatting must be checked. Some formatting is incorrect (see L508).

Answer: This has been corrected.

Results
Table 2: reports telomere but the identification of telomeres is not described in the methods. ** shows verification with poreC and quartet what about the others? Have they been identified in another way?

Answer: The identification of telomeres was performed using quartet (RRID:SCR_025258).
‘*’ and ‘**’ explanations have been changed as follows: * - telomeres with a non-canonical ACGCAGC/TGCGTCG motif; ** - centromeres verification based only on quarTeT and Pore-C contact map.
Tandem centromeric repeats were provided in the Supplementary Table S1.

Fig5 Do tips labelled with the colored circles represent the centromere localized LTRs?

Answer: Yes, the marked tips represent centromere enriched LTRs. This has already been stated in the caption of the figure.

Fig6 needs some further explanations in the legend. Lines, dots are not clearly explained. Also, meaning of the shaded area and that of the Y axis is not explained.

Answer: Additional explanation has been provided: ‘In the density profile, each dot represents a single genomic window, plotting its LTR/Gypsy density (X-axis) against its chromosomal position (Y-axis). The solid dark red line shows the overall trend in density, calculated using a LOESS regression, while the surrounding light grey shaded area indicates the 95% confidence interval for this trend. The thick red horizontal bar marks the position of the centromere across the center of the magnified plots.’

Fig 8. The legend states that colored line thickness reflects collinearity. I think the ribbons just connect collinear regions, but their thickness has nothing to do with the strengths/extent of collinearity at all. Please, revise it.

Answer: The legend has been revised: ‘Figure 8. Synteny plot of the differences and similarities between the assembly of A. endiviifolia chromosomes and that of H. hutchinsiae. Colored lines connect collinear regions between the species' genomes.’

Fig9. The legend does not state that boxes refer to genome sizes of the species. Also, a scale to the genome sizes is missing. Revise.

Answer: The legend has been revised: ‘Figure 9. Phylogenetic relationships among liverworts determined by conserved single-copy orthologs. Size of orange and blue boxes corresponds to genome size variations in liverwort species. All nodes have the maximum statistical support.’
The genome size scale is shown in the figure. It is the black box representing 1000 Mbp.

FigS1 The legend must be revised and extended to provide more information on which

Powered by Editorial Manager® and ProduXion Manager® from Aries Systems Corporation

symbol refers to which feature of the genome. Also, it is not clear whether the pore-c contact map spans the entire chromosome or not as it lacks a scale. I would also suggest changing the representation a bit as the figure mainly focuses on the density of Gypsy elements, but it does not show the tandem-nested putative centromeric repeats. Finally, the gypsy element density appears to be only considerably high on one side of the putative centromere while the other side is not that rich. I think this should be mentioned as this type of centromere identification is ambiguous. This is fine but must be acknowledged.

Answer: The pore-C range is now better explained on Figure S1 and Figure 6 and is no longer misleading. We also provide detailed captions to Gypsy density plot. ‘In the density profile, each dot represents a single genomic window, plotting its LTR/Gypsy density (X-axis) against its chromosomal position (Y-axis). The solid dark red line shows the overall trend in density, calculated using a LOESS regression, while the surrounding light grey shaded area indicates the 95% confidence interval for this trend. The thick red horizontal bar marks the position of the centromere across the center of the magnified plots.’

Responses to Reviewer #2:
Reviewer #2: The authors present a telomere-to-telomere (T2T) genome assembly of the liverwort Apopellia endiviifolia using Oxford Nanopore long-read sequencing combined with Pore-C proximity ligation data. The study reports a complete assembly of nine chromosomes with telomeres and centromeres identified, alongside genome annotation and synteny analyses. This work represents a valuable addition to bryophyte genomics, particularly given the scarcity of high-quality T2T assemblies for liverworts.

However, several aspects of the manuscript require substantial clarification and expansion before it can be considered for publication. In its current form, the manuscript reads more like a technical report than a genomic resource paper that provides novel biological insights. Key biological findings are underdeveloped, figures lack clarity, and comparisons with existing T2T plant genomes are minimal. I recommend major revisions.

Answer: We sincerely appreciate your insightful and constructive feedback, which has been instrumental in refining and strengthening our manuscript. Below, we detail the revisions made in response to your comments.

Major Comments:
1. The background section devotes substantial space to discussing speciation mechanisms and RNA editing. However, these topics are not addressed in the results or discussion, leaving the reader with unfulfilled expectations. If these topics are retained in the introduction, relevant analyses or interpretations should be provided in later sections; otherwise, the background should be streamlined.

Answer: Thank you for highlighting this point. We have taken your suggestion into account and consequently removed the section on RNA editing from the Introduction to maintain focus and avoid raising expectations that are not addressed later in the manuscript.

2. The authors report non-canonical telomeric sequences (ACGCAGC and TGCGTCG) on chromosomes 3 and 6, which is an unusual and potentially important finding. However, no biological interpretation or comparison with other bryophytes or plant lineages is provided. This omission weakens the impact of the study. The authors should at least speculate on possible mechanisms or evolutionary significance, or cite similar patterns in other species if known.

Answer: We have added commentary on telomeres in the 're-use potential' section: ‘The unusual presence of non-canonical telomeric-like repeats (ACGCAGC and TGCGTCG) on chromosomes 3 and 6 in A. endiviifolia suggests species-specific telomere evolution or chromosomal rearrangements, though their biological significance is unclear. Further comparative and functional studies are needed to understand their origin and role. These findings contribute to the genomic landscape of

A. endiviifolia and support future research on plant telomere diversity and evolution. Notably, the centromeres have been predicted solely through in silico analyses, representing a plausible but not yet fully validated hypothesis.'

3. While the authors identify centromeres and note Gypsy/Tat family enrichment, there is minimal discussion of how this compares to other bryophyte or land plant centromeres. For example, how does the 1.9-5 Mbp centromere size compare to Riccia, Marchantia, or Physcomitrium? Are these unusually large? What might this suggest about karyotype or genome evolution? The current treatment is descriptive but lacks insight.

Answer: This article has been prepared in 'Data Note' format. As required by the publisher, this type of article in GigaScience does not include discussion within its structure. The section on actor instructions includes the following information: 'Data Notes focus on a particular dataset, and provide detailed methodology on data production, validation, and potential reuse. … Manuscripts containing more detailed biological, medical or technical analyses of data should be submitted as a Research Article. One of the aims of a Data Note is to incentivize and more rapidly release data before subsequent detailed analysis has been carried out.'

4. The synteny analysis between A. endiviifolia and other liverworts is reduced to a brief mention of 'fragmented collinearity.' No quantification of syntenic block sizes, gene retention, or structural rearrangements is provided. Similarly, the phylogenetic tree, though technically sound, is not interpreted in depth. What does this tree reveal about genome size evolution or divergence times? The authors should expand the discussion and contextualize their findings.

Answer: Details of the detected syntenic blocks between A. endiviifolia and H. hutchinsiae chromosomes, including chromosome coordinates and strand orientation, are provided in Supplementary Table S2.

Minor Comments
Background
-"dioecious" should be corrected to "dioicious."

Answer: This has been corrected.

-The term "basal phylogenetic position" is problematic. In phylogenetic trees, clade relationships are dichotomous, and describing a clade as "basal" is misleading.

-The authors state, "They hold a crucial position in plant evolutionary history as sister groups to all other extant land plants." However, the phylogenetic relationships among the three bryophyte lineages and their placement relative to other land plants have been the subject of long-standing debate. Recent genomic analyses tend to support bryophyte monophyly, with liverworts and mosses forming a clade.

Answer: Your comments have been taken into account, the beginning of the introduction has been edited as follows: 'Liverworts (Marchantiophyta) represent one of the earliest terrestrial plants, with fossil evidence indicating their emergence in the Middle Ordovician period approximately 419–447 million years ago [1]. They occupy a pivotal position in plant evolutionary history as members of the monophyletic bryophyte lineage, which, along with vascular plants, form two distinct monophyletic groups within terrestrial plants. Within bryophytes, liverworts are sister to mosses, forming the Setaphyta clade, which in turn is sister to hornworts. This phylogenetic framework is supported by recent genomic analyses that affirm the monophyly of bryophytes and clarify the evolutionary relationships among early land plants [2]. Liverworts, along with other bryophytes such as mosses and hornworts, exhibit a suite of morphological and physiological features that shed light on plant adaptation to terrestrial environments, although it remains debated whether these features represent ancestral traits or are the result of secondary reductions, with recent evidence favoring the latter [3]. This group is often characterized by structural simplicity, a predominantly haploid life cycle, and a slow rate of molecular evolution, which corresponds to gradual morphological diversification [4].

-The authors write, "Its chloroplast genome exhibits an intermediate level of RNA editing, bridging the gap between non-editing liverworts, such as Marchantia polymorpha, and seed plants." In liverworts, only the complex thalloid lineage lacks RNA editing; most other liverworts possess RNA editing sites in their organellar genomes. Please refer to:
Dong SS, Zhao CX, Zhang SZ, Wu H, Mu WX, Wei T, Li N, Wan T, Liu H, Cui J, Zhu RL, Goffinet B, Liu Y. 2019. The amount of RNA editing sites in liverwort organellar genes is correlated with GC content and nuclear PPR protein diversity. Genome Biology and Evolution 11: 3233-3239.

Answer: The text section regarding RNA editing has been removed.

Material and Methods
-Was a voucher specimen prepared for the sequenced sample? If so, please indicate where it is deposited.

Answer: A voucher specimen was prepared for the sequenced sample. It has been deposited in the Herbarium of University of Warmia and Mazury in Olsztyn (OLS), under the accession number OLS-H2024P004.

-Although the experiments appear to have used axenic culture of the liverwort (please confirm if this is correct), the culture was not initiated from a single spore/shoot. This implies that the material may represent a population of the species. When assembling such sequence reads, how were polymorphisms among the reads handled? Please clarify.

Answer: Yes, the experiments appear to have used axenic culture of the liverwort. And also the culture was initiated from a single fragment of the thallus, that was used for the growth and development of the plant, which was then multiplied in vitro. The plants used in the study were genotypically identical.

-For RNA-seq, the tissue type used for RNA extraction was not specified. Please provide this information.

Answer: This information has been supplemented: ‘Total RNA for short read procedure was extracted from four samples of male A. endiviifolia: land (i) and water (ii) form, antheridia (iii) and surrounding thallus (iv). This extraction was conducted using the RNA Plant Mini Spin Kit (Qiagen) according to the manufacturer’s protocol.’

-The statement "A custom Python script (analyze_genome_wide_ltr_domains.py) was developed" should be accompanied by information on where this script is deposited.

-Similarly, for "Custom Python scripts were used to extract the protein sequences of the single-copy orthologs shared across all 14 species", please clarify what these scripts are and where they are deposited.

Answer: All custom scripts used have been provided with citations indicating where they were deposited. [50 - GitHub - kubek78/PhylogenomicsME: Phylogenomics Made Easy. https://github.com/kubek78/PhylogenomicsME/tree/main]

Results
-The manuscript mentions six remaining gaps but does not indicate whether these are intergenic, pericentromeric, or subtelomeric. Given the emphasis on T2T quality, this should be specified.

Answer: We clarified that these were intergenic regions.

-The reported 68.5 Gbp of Nanopore raw data would theoretically yield only ~20× average depth, and the reads have a mean quality score of 19. This raises concerns about sequencing accuracy; in such cases, ~100× coverage with WGS short reads would be advisable to improve assembly accuracy.

Answer: Thank you for pointing it out. We provide SRA accession number for the second nanopore library we used for polishing and final coverage calculation (the value

	was calculated using both libraries) but the SRR35424238 wasn't used in initial assembly steps, due to lower N50 of raw reads.<br><br>Figures and other:<br>-The manuscript alternates between "Helitron" and "Helintron" in the text and figure captions. Please ensure consistent and correct use of TE family names.<br><br>Answer: Thank you for pointing that out. It was a typo. The correct name is 'Helitron'.<br><br>-Several key figures (e.g., dot plots, Circos plots) are difficult to interpret due to low resolution and unclear labeling. Axis labels, legends, and color schemes should be revised for clarity and readability.<br><br>Answer: We acknowledge that the current figures provided are of lower resolution for the initial review. We will prepare and submit higher-resolution versions of the key figures to enhance clarity and readability during the production stage.<br><br>-Data availability is incomplete. Only a submission ID (SUB15403958) is provided for the genome assembly. Please include complete accession numbers for raw data, assemblies, and annotations (e.g., BioProject, BioSample, GCA/GCF IDs) before publication.<br><br>Answer: Information has been added: For A. endiviifolia genome assembly 'The assembled genome and gene annotation can be found in the NCBI assembly with submission number JBRAUX000000000.' For RNA-seq 'The raw reads were deposited in BioProject PRJNA1279829 and BioSample SAMN49506713.' For M. polymorpha 'GCA_037833965.1' and for H. hutchinsiae 'GCA_965112325.1'.<br><br>Language and phrasing issues:<br>-Some phrases are awkward or unclear. For example, in the Findings section: "gene density varied between a value 5.52 and 9.78" should be revised to "gene density ranged from 5.52 to 9.78 genes per xx kbp." Similar issues occur in lines 383-384. A careful language edit throughout the manuscript would improve readability.<br><br>Answer: This has been corrected: '5.52 and 9.78 genes per 500 kbp.'
**Additional Information:**	
**Question**	**Response**
Are you submitting this manuscript to a special series or article collection?	No
**Experimental design and statistics**<br><br>Full details of the experimental design and statistical methods used should be given in the Methods section, as detailed in our Minimum Standards Reporting Checklist. Information essential to interpreting the data presented should be made available in the figure legends.<br><br>Have you included all the information requested in your manuscript?	Yes
**Resources**	Yes

A description of all resources used, including antibodies, cell lines, animals and software tools, with enough information to allow them to be uniquely identified, should be included in the Methods section. Authors are strongly encouraged to cite Research Resource Identifiers (RRIDs) for antibodies, model organisms and tools, where possible.<br><br>Have you included the information requested as detailed in our Minimum Standards Reporting Checklist?	
**Availability of data and materials**<br><br>All datasets and code on which the conclusions of the paper rely must be either included in your submission or deposited in publicly available repositories (where available and ethically appropriate), referencing such data using a unique identifier in the references and in the “Availability of Data and Materials” section of your manuscript.<br><br>Have you have met the above requirement as detailed in our Minimum Standards Reporting Checklist?	Yes
GigaScience has policies and guidelines in place for the use of generative AI-writing tools such as ChatGPT. If you have used such writing tools to assist with writing the manuscript this must be declared and cited in the text. Authors should not list AI-writing tools and other AI-assisted technologies as an author or co-author and should acknowledge that they are fully responsible for text generated or refined by AI-writing tools.<p><br><br>A summary of use (particularly in the introduction or among methods) needs to be included at the end of the paper, and the outputs should also be included as a supplementary file hosted in GigaDB or other open repositories. Please read our guidelines for more information. <p>	No

By submitting to GigaScience, you are aware of the journal's AI-writing tools policy, and if you have declared use of such tools below, you have acknowledged this where appropriate in your manuscript and have made a summary of use and outputs available. <p><br><b>AI-assisted writing tools have been used in the preparation of this manuscript?	

Manuscript Click here to access/download;Manuscript;Szablinska_Apopelia_genome_re

Giant chromosomes of a tiny plant - the complete telomere-to-telomere genome assembly of the simple thalloid liverwort *Apopellia endiviifolia* (Jungermanniopsida, Marchantiophyta)

Joanna Szablińska-Piernik[1,*], Paweł Sulima[2], Jakub Sawicki[1,*]

1. Department of Botany and Evolutionary Ecology, University of Warmia and Mazury in Olsztyn, Plac Łódzki 1, Olsztyn, 10-719, Poland

2. Department of Genetics, Plant Breeding and Bioresource Engineering, University of Warmia and Mazury in Olsztyn, Plac Łódzki 3, Olsztyn, 10-724, Poland

* corresponding authors: joanna.szablinska@uwm.edu.pl, jakub.sawicki@uwm.edu.pl

Keywords: *Apopellia endiviifolia*, liverworts, telomere-to-telomere [T2T], genome assembly, centromere

Abstract

Background

The liverwort *A. endiviifolia*, a dioicous, simple thalloid species, is notable for its cryptic diversity, habitat adaptability, genomic innovation, and represents a clade that is a sister to all other Jungermanniopsida. These features make *A. endiviifolia* an essential model for exploring speciation mechanisms and the evolution of genome structures within liverworts.

Findings

We present the genome assembly of haploid *A. endiviifolia* isolate with a total size of 2,914,960,273 bp and an N50 of 468,157,909 bp, demonstrating high completeness (99.2% BUSCO) and a high consensus quality (QV 47.6). The assembly consisted of nine chromosomes, which included validated 18 telomeres and nine centromeres (ranging from 1.9 to 5 Mbp in length). RNA-seq-based annotation identified 34,615 genes, predominantly protein-coding. The TEs comprised 12.16% LTR elements and 57 Helitrons. Among the retroelements, the *Copia* and *Gypsy* superfamilies comprised 8.94% and 2.95% of the genome, respectively. The Ty3/*Gypsy* superfamily was found to be significantly enriched in centromeric regions. The average GC content ranged from 38.8% to 39.6%, with gene density varied between a value 5.52 and 9.78 genes per 500 kbp. Synteny analysis of related liverwort species

has revealed complex chromosomal relationships, indicating extensive genome rearrangements among species.

Conclusions

This study provides the first high-quality reference genome assembly of the haploid liverwort *A. endiviifolia*. Assembly and annotation offers valuable resources for investigating liverwort evolution, centromere biology, and genome expansion in simple thalloid liverworts.

Background

Liverworts (Marchantiophyta) represent one of the earliest terrestrial plants, with fossil evidence indicating their emergence in the Middle Ordovician period approximately 419-447 million years ago [1]. They occupy a pivotal position in plant evolutionary history as members of the monophyletic bryophyte lineage, which, along with vascular plants, form two distinct monophyletic groups within terrestrial plants. Within bryophytes, liverworts are sister to mosses, forming the Setaphyta clade, which in turn is sister to hornworts. This phylogenetic framework is supported by recent genomic analyses that affirm the monophyly of bryophytes and clarify the evolutionary relationships among early land plants [2]. Liverworts, along with other bryophytes such as mosses and hornworts, exhibit a suite of morphological and physiological features that shed light on plant adaptation to terrestrial environments, although it remains debated whether these features represent ancestral traits or are the result of secondary reductions, with recent evidence favoring the latter [3]. This group is often characterized by structural simplicity, a predominantly haploid life cycle, and a slow rate of molecular evolution, which corresponds to gradual morphological diversification [4]. However, such generalizations primarily reflect the characteristics of the Haplomitriopsida and Marchantiopsida classes (complex thalloid liverworts) [5]. Significant variation exists within the broader liverwort lineage, particularly in Jungermanniopsida, which includes leafy and simple thalloid liverworts. This class comprises the vast majority (>80%) of extant liverwort species, and demonstrates a significantly higher degree of structural complexity and a notably accelerated rate of molecular evolution. This is evidenced by their diverse leafy morphologies, intricate branching patterns, and specialized reproductive structures, which facilitate their adaptation to a wide array of ecological niches [6–8]. Within Jungermanniopsida, the order Pelliales consists of two families: *Noterocladaceae* and *Pelliaceae* [9]. Recent molecular and morphological studies have refined the taxonomy within *Pelliaceae*, distinguishing *Apopellia* as a separate genus that includes three species: *A. apicola*, *A. megaspora*, and *A. endiviifolia* [10].

A. endiviifolia is a dioicous, simple thalloid liverwort characterized by its cuneate apical cell, the absence of a midrib in the thallus, a spherical capsule, and a robust seta. It is widely distributed across the Northern Hemisphere and thrives in a diverse array of habitats, including aquatic environments such as springs and streambanks, as well as in dry conditions often associated with limestone substrates. Its capacity to grow on limestone rocks, arid soils, aquatic habitats (hydrophytes), and decaying wood (epixyl) underscores its remarkable ecological versatility [11]. This species exemplifies cryptic speciation, with European populations diverging into two lineages: A (typical form) and B (water form), which are differentiated by molecular markers and microhabitat preferences [8,10,12,13]. This pattern reflects broader taxonomic revisions within *Pellia* s.l., which have been split into *Apopellia* and *Pellia* s.s. through integrative approaches, highlighting its importance in studying speciation mechanisms [10]. The combination of cryptic diversity, habitat versatility, and genomic novelty renders *A. endiviifolia* a valuable model for exploring speciation mechanisms and structural genomic evolution in liverworts.

Bryophytes, including liverworts, mosses, and hornworts, are generally characterized by relatively small nuclear genomes compared to other plant groups. However, significant variation exists within these groups, particularly among liverworts. On average, hornwort genomes measure 244 Mbp (median 205 Mbp), moss genomes average around 504 Mbp (median 433 Mbp), and liverwort genomes tend to be larger, averaging 1,844 Mbp with a median of 751 Mbp [14]. Flow cytometry data further reveal that liverwort genome sizes vary widely, ranging from 206.2 Mbp in *Lejeunea cavifolia* to 20,006 Mbp in *Phyllothallia fuegiana* [15,16]. Within liverworts, the *Pelliaceae* family is notable for its particularly large genomes: *Pellia borealis* has a genome size of 7,238.3 Mbp, *P. epiphylla* 3,719.2 Mbp, and *A. endiviifolia* 3,364.0 Mbp [15]. Despite efforts to determine the nuclear genome size of over 100 liverwort species, comprehensive genomic resources remain limited, with only a handful of liverwort genomes sequenced at the chromosomal level [17–21].

Although the variation in DNA content within the liverwort genome is lower than that in the nuclear genomes of angiosperms [22], the variation in genome size among liverworts is substantial and an intriguing area of research, particularly given the limited understanding of the patterns and rates of structural changes within these genomes [16]. Studies of genome evolution, particularly in complex thalloid liverworts, have revealed the absence of ancient whole-genome duplication events, minimal rates of gene duplication and chromosomal rearrangements, and rare occurrences of transposable element (TE) bursts. A comparative analysis of the nuclear genomes of the model liverwort *M. polymorpha* (286.7 Mbp) and

Lunularia cruciata, which is distinguished by a genome size nearly twice as large (565.6 Mbp), highlight the role of *Ty3/Gypsy* retrotransposon proliferation in genome size expansion [4]. Furthermore, recent advances in long-read sequencing technologies have enabled the assembly of entire genomes at the telomere-to-telomere scale, providing unprecedented insights into highly repetitive regions such as centromeres and telomeres [23]. These advances have revealed a correlation between centromere structure and chromosome length evolution, with findings suggesting that chromosomes possessing longer centromeres tend to contain a higher proportion and greater length of *Copia* transposable elements enriched within their centromeric regions. This enrichment of *Copia* elements may contribute to a positive association between centromere length and overall chromosome length, potentially influencing karyotype evolution [24]. In the field of bryophyte research, the application of near telomere-to-telomere genome assembly has facilitated the identification of centromere sequences in the moss species, *Physcomitrium patens*. This has enabled the precise characterization of its centromeres, revealing 26 monocentric chromosomes, each containing a single centromeric region enriched with RLC5 retrotransposons from the *Bryco* clade of the *Copia* superfamily [25]. Moreover, this multi-method study elucidated the evolutionary dynamics of centromeres in non-seed plants, highlighting their unique composition and recent evolution. It also provides a gap-free genomic framework for investigating its role in chromosome stability and segregation. In contrast, a study of *M. polymorpha* revealed that its centromeres consist of simple 162-bp satellite repeats and lack extensive pericentromeric heterochromatin and Long Terminal Repeats (LTRs) retrotransposon enrichment typical of flowering plants. Instead, these centromeres are flanked by a specific LINE transposon family [26].

To further advance our understanding of genome evolution and the pivotal role of repetitive elements in the centromere architecture and biology of liverworts, our study provides the first comprehensive, high-quality reference genome assembly of the haploid liverwort *A. endiviifolia* using primary Oxford Nanopore long-read sequencing and Pore-C technology. This high-quality genome assembly and annotation serves as a vital resource for further exploration of liverwort evolution, and provides new perspectives on centromere biology and genome expansion mechanisms in simple thalloid liverworts.

Material and Methods

Sample collection and *in vitro* culture

A. endiviifolia plants were collected from the Nature Reserve of the Sources of the Łyna River (NE Poland; 54.6208°N, 21.2267°E). *In vitro* cultures of *A. endiviifolia* were established in the

Plant Biotechnology Laboratory at the Department of Genetics, Plant Breeding and Bioresource Engineering, University of Warmia and Mazury in Olsztyn (Poland). *A. endiviifolia* fragments were washed for 15 min in tap water, surface-disinfected with 0.5% calcium hypochlorite and 0.05% TWEEN-20 for 10 min, and then triple-rinsed with sterile distilled water (5, 10, and 15 min). The sterilization method was highly effective, achieving a culture sterility of 95.65%. Sterile explants were cultured on solid ½ Gamborg's B5 medium (½ basal salts, organics, vitamins [27], 20 g/l sucrose, 8 g/l agar, pH 6.0) at 24°C under a 16h light/8h dark. The upper segments of the sterilized plants served as secondary explants for micropropagation on the same medium, spaced 1–2 cm apart. Micropropagated plants were used in the subsequent experiments (Fig. 1).

A voucher specimen for the sequenced sample has been prepared and deposited in the Herbarium of the University of Warmia and Mazury in Olsztyn (OLS), with the accession number OLS-H2024P004.

DNA extraction

For genome assembly, genomic DNA was extracted from the aerial parts of the thallus using a modified ultra-long DNA extraction protocol [28]. Briefly, 500 mg of the material was ground into a fine powder in liquid nitrogen and incubated in 30 mL of homogenization buffer (HB) for 15 min on ice. The suspension was then filtered through a 40 μm cell strainer. Following centrifugation (3,000 × g) and two wash cycles with HB buffer, the pellet was dissolved in SDS lysis buffer containing 5 μL RNase A (20 μg/μL) and 75 μL proteinase K (20 μg/μL) and incubated at 50°C for 3 h. Subsequently, the DNA was extracted using chilled phenol:chloroform:isoamyl alcohol. The DNA was precipitated with isopropanol, washed with 80% ethanol, and eluted in 50 μL of water. The fragment length distribution and DNA integrity were assessed using TapeStation with the Genomic DNA ScreenTape Assay (Agilent), reaching a maximum intensity peak with length > 60,000 bp and DIN 8.2, respectively. The concentration was determined using the Qubit fluorometer HS DNA assay kit and amounted 65.2 ng/μL.

Pore-C procedure

The restriction enzyme Pore-C protocol for plant samples (RE-Pore-C, ONT) was employed to capture three-dimensional DNA interactions within chromatin, with a few modifications as previously described by Krawczyk et al. [18]. Chromatin was preserved with formaldehyde and the crosslinked plant material was cryogenically ground. The resulting suspension was

filtered through a 40 μm strainer, purified, and digested with the NlaIII (NEB) restriction enzyme for 18 hours at 37°C, followed by heat denaturation. Subsequently, a proximity ligation reaction was performed using 40,000 U of T4 DNA ligase for 6 hours at 16°C. This was followed by protein degradation and chromatin de-crosslinking with 100 μl proteinase K (20 μg/μL) and 3 μl RNase A (100 μg/μl) for 18 hours at 56°C, with additional rounds of proteinase K digestion, as per the HiPore-C v1 protocol [29]. DNA was extracted using chilled phenol:chloroform:isoamyl alcohol and EDTA, precipitated with NaCl, washed with ethanol, and eluted with TE buffer. DNA quality was assessed using TapeStation, which reached a peak length of 11,142 bp and DIN 5.8. The concentration was 43 ng/μL, as measured using a Qubit fluorometer.

Nanopore sequencing

Libraries were prepared for nanopore sequencing of native DNA and proximity-ligated DNA fragments using the Ligation Sequencing Kit V14 (SQK-LSK114) following the manufacturer's protocol. DNA sequencing data were generated using the Oxford Nanopore Technologies PromethION 2 platform on R10.4.1 flowcells (PRO-114M) and the MinKNOW sequencing software.

RNA-seq

Total RNA for short read procedure was extracted from four samples of male *A. endiviifolia*: land (i) and water (ii) form, antheridia (iii) and surrounding thallus (iv). This extraction was conducted using the RNA Plant Mini Spin Kit (Qiagen) according to the manufacturer's protocol. Short-read RNA-seq libraries were prepared using two distinct protocols. For RNA extracted from the water and land form of *A. endiviifolia*, libraries were constructed using the TruSeq Stranded Total RNA Library Prep Kit (Illumina) using the Ribo-Zero rRNA option, whereas for antheridia and surrounding thallus, which amount of extracted RNA was insufficient for TruSeq, libraries were prepared using the QIAseq FX Single Cell RNA Library Kit (Qiagen). All libraries were sequenced on an Illumina NovaSeq 6000 platform (Macrogen, Inc., Seoul, South Korea) in 2 × 150 pair-end mode. Raw reads were deposited in BioProject PRJNA1279829 and BioSample SAMN49506713.

Genome size estimation, assembly and quality evaluation

Basecalling

Raw Nanopore signal data from both the standard and Pore-C libraries were basecalled using Dorado v0.9.1 (RRID:SCR_025883) [30]. The superior accuracy model SUP dna_r10.4.1_e8.2_400bps_sup@v5.0.0, was employed to generate high-fidelity basecalls in BAM format. In the case of high molecular weight sequencing reads, the DNA modification v3 models were used to detect all context 6mA, 4mC, and 5mC methylation at the single-base accuracy level.

A fastq file was used to count K-mer frequencies using *kmerfreq* v4.0 [31]. Subsequently, the genome size was estimated using the *GCE* v1.0.2 program [32] and kmerfreq files. The estimated genome size for k-mers from 14 to 21 fell within the 2,950-3,090 Mbp range.

Initial Contig Assembly with Hifiasm

An initial *de novo* assembly was generated directly from the base-called Nanopore long reads using Hifiasm v0.25.0 (RRID:SCR_021069) [33], which supports assembly from high-quality Nanopore data using the–ont option. Simplex basecalled reads were used as input. Hifiasm was run with parameters suitable for Nanopore reads -t64–l0. This produced a set of high-quality initial contigs. To organize the initial contig assembly into chromosome-level structures, we utilized proximity ligation sequencing reads from the Pore-C library processed using the epi2me-labs/wf-pore-c pipeline v1.3.0 [34] to generate a BED file of pairwise chromatin contacts. This contact map served as the input for scaffolding with YaHS v1.2.2 (RRID:SCR_022965) [35] with three iterative rounds. Following automated scaffolding, the resulting assembly and the Pore-C contact map were loaded into Juicerbox v2.17 [36] for manual inspection and curation. Gap-closing of assembly was performed using SAMBA with default options with exception of -m parameter (minimum matching length) set to 9000 as recommended for large plant genomes [37].

The gap-closed assembly was polished using Dorado v0.9.1 aligner and polishing functionalities. The required input for polishing, a sorted BAM file containing alignments of basecalled reads with necessary metadata, was generated using a dorado aligner. This dorado aligner output BAM, along with the gap-closed assembly FASTA, was then processed using the dorado polish command to achieve the final polished assembly.

Quality check

To ensure high quality of the final genome assembly, we employed a multi-faceted approach to evaluate the completeness, structural integrity, and assembly of repetitive regions. Gene content completeness was assessed using BUSCO v5.8.2 (RRID:SCR_015008) [38] with the

viridiplantae_odb10, eukaryota_odb10, and embryophta_odb10 lineage datasets. The structural accuracy and continuity of the nine assembled chromosomes were evaluated using Inspector v1.3.1 [39]. This was performed in a reference-free manner by mapping the long reads back to the assembly to identify potential misassemblies, structural variants, and other inconsistencies. Finally, to assess the completeness of the repetitive landscape, the LTR Assembly Index (LAI) was calculated using the EDTA v2.2.2 (Extensive *de novo* TE Annotator, RRID:SCR_022063) package [40].

Genome annotation

Structural and functional annotation of the genome was conducted using the NCBI EGAPx v0.3.2 pipeline [41] empirical evidence from all four RNA-seq libraries. Based on the provided taxonomy ID, the pipeline automatically selected appropriate protein sets for homology-based evidence, which were aligned to the assembly using miniprot v0.15 [42]. The RNA-seq reads were aligned using STAR v2.7.11 (RRID:SCR_004463) [43] to generate transcript-based evidence. The core of the annotation was performed using Gnomon [44], which first chained the protein and transcript alignments into putative gene models. Gnomon supplemented these with *ab initio* predictions derived from HMM models to identify genes lacking direct evidence. Finally, functional information was added based on the model quality and orthology, and the complete annotation set was generated as a GFF3 file.

Repeatome annotation

To identify and characterize repetitive elements within the *A. endiviifolia* genome, comprehensive repeatome annotation was performed. Initially, repetitive sequences were identified using EDTA v2.2.2 (RRID:SCR_022063) [40]. Further refinement and classification of transposable element (TE) families were performed using TESorter [45], which leverages machine learning to accurately classify diverse TE types. A phylogenetic approach was employed for the specific annotation of LTR retrotransposons. Individual LTR sequences were first aligned using MAFFT (RRID:SCR_011811) [46] with the default settings. Subsequently, based on the obtained alignment and evolutionary relationships among the distinct LTR retrotransposon families, phylogenetic trees were constructed using IQ-TREE 2 (RRID:SCR_017254) with 1000 bootstrap replicates [47].

Identification and characteristics of telomeres and centromeres

The telomeric sequences and centromeric regions within the *A. endiviifolia* genome assembly were identified using quartet (RRID:SCR_025258) [48]. The centromeres of each chromosome were further analyzed and confirmed using CentIER [49]. Both software were used with default settings.

A custom Python script (analyze_ltr_enrichment.py) [50] was developed and employed to identify LTR retrotransposon domain families that are preferentially associated with centromeric regions. Initially, all LTR element sequences were extracted from the whole-genome LTR GFF3 annotation file using reference genome assembly and subjected to six-frame translation. The resulting protein sequences were scanned for conserved LTR protein domains using HMMER (hmmsearch) against the REXdb database. This process generated a comprehensive genome-wide map linking LTR elements (identified by their unique GFF IDs) to their constituent REXdb protein domain types (e.g., Athila, Bryco, and SIRE). Next, these genome-wide LTR domain annotations were used for enrichment analysis within the predefined centromeric regions. For each REXdb domain type, the script calculated the total occupied length and the total count of distinct LTR elements containing that domain separately for centromeric and non-centromeric portions of the genome. The noncentromeric portion was determined by subtracting the total length of the defined centromeric regions from the total genome size. To assess the statistical significance of domain enrichment in centromeres, Fisher's exact test (one-tailed, testing for enrichment) was performed for each domain type based on both its total length and element count in centromeric versus non-centromeric regions.

Synteny analysis

The genomic sequences (FASTA) and gene annotation files (GFF3) for *M. polymorpha* (Tak-1/2 v7.1, GCA_037833965.1) and *A. endiviifolia* (JBRAUX000000000) were obtained from GenBank. The protein sequences for each species were extracted from their respective genomic FASTA and GFF3 files using gffread v0.12.7 (RRID:SCR_018965) [51]. For each gene, the longest protein isoform was retained for downstream analysis.

Gene coordinates were converted into BED6 format using the jcvi.formats.gff bed (v1.5.4 from JCVI utilities [52]) from the GFF3 files. The gene identifiers in these BED files were subsequently cleaned to remove prefixes (e.g., "rna-") and complex locus tag components to ensure compatibility across tools. Specifically, *Marchantia* gene IDs were processed in the format MPTK2_... and *A. endiviifolia* gene IDs were processed in egapxtmp_...-R... by using custom awk scripts. These cleaned BED files provided the chromosome, start, end, cleaned gene ID, dummy score, and strand information. Orthologous gene pairs between *M.*

polymorpha and *A. endiviifolia* were identified using OrthoFinder v3.0.1b1 (RRID:SCR_017118) [53]. The extracted protein sequences from both the species were used as inputs. OrthoFinder was run with DIAMOND v2.1.11 (RRID:SCR_016071) [54] as the sequence search tool for all-vs.-all protein comparisons. Syntenic blocks between *M. polymorpha* and *A. endiviifolia* were identified using the scan action within the jcvi.compara.synteny module (v1.5.4; JCVI utilities [52]). The cleaned BED files and pairwise ortholog files were used as inputs. The analysis was performed using relaxed parameters that are suitable for distantly related species. A minimum of two collinear gene pairs (--min_size=2) was required to define a syntenic block, and a maximum gap of 100 non-collinear genes (--dist=100) was allowed within a block. The output anchor file, containing the identified syntenic gene pairs, was retained. The identified syntenic relationships were visualized as a dot plot using jcvi.graphics.dotplot (v1.5.4, JCVI utilities [52]).

Synteny analysis of *A. endiviifolia* and the second available T2T genome assembly of Jungermanniopsida, *H. hutchinsiae* was performed using ntSynt v1.0.2 [55] since gene annotations file wasn't available in GenBank (GCA_965112325.1).The ntSynt workflow begins by generating ordered minimizer sketches for each genome. These sketches were filtered to retain only the single-copy minimizers present in all assemblies, which were then used to construct an initial graph. After simplifying this graph, linear paths were identified to compute an initial set of synteny blocks. The algorithm refines these by reanalyzing regions not covered by the initial blocks with a smaller window size and augmenting the graph. The pipeline was run with default settings and -d 40 divergence value, and the resulting synteny blocks were then visualized using the ntSynt-viz v1.0.0 pipeline [55]. The *Apopellia* genome was used as the reference, and the strands of chromosomes in the *Herbertus* genome were normalized relative to it. The final visualization of syntenic relationships was rendered as a ribbon plot using gggenomes v1.0.1 [56] R package.

Phylogenomic analysis

Chromosome-scale genome assemblies for 13 liverwort species ranging in size from 200 to 3 Gbp were obtained from the GenBank genome database in FASTA format. Single-copy orthologous genes were identified using BUSCO v5.4.7 (Benchmarking Universal Single-Copy Orthologs, RRID:SCR_015008) [38] with the Embryophyta_odb10 database, which contains 1,614 conserved ortholog groups from 50 plant species. BUSCO was run in genome mode with 32 threads using the default parameters. Custom Python scripts [50] were used to extract the protein sequences of the single-copy orthologs shared across all 14 species. Only

orthologs present in a single copy in all genomes were retained for downstream analysis, resulting in 502 shared single-copy orthologs. Individual protein sequences for each ortholog were aligned using MAFFT v7.505 (RRID:SCR_011811) [46] with the automatic algorithm selection option (--auto), which selects the optimal alignment strategy based on sequence characteristics. The alignments were subsequently trimmed to remove poorly aligned regions using trimAl v1.4 (RRID:SCR_017334) [57] with the automated1 option, which applies a heuristic selection of the optimal automated trimming method. Trimmed alignments of all 502 single-copy orthologs were concatenated into a supermatrix using custom Python scripts. Partition information was recorded for potential partitioned phylogenetic analyses with each gene treated as a separate partition. Maximum likelihood phylogenetic analysis was performed using IQ-TREE v2.2.0 (RRID:SCR_017254) [47] with automatic model selection using ModelFinder [58] implemented in IQ-TREE (-m MFP option). The analysis included 1,000 ultrafast bootstrap replicates [59] to assess branch support (− B 1000). The final phylogenetic tree was visualized using the R package ape v5.6 (RRID:SCR_017343) [60], phangorn v2.10 (RRID:SCR_017302) [61], and phytools (RRID:SCR_015502) [62]. All custom scripts used in this analysis are available at Github repository [50].

Results

Complete reference genome assembly and annotation for *A. endiviifolia*

The integration of 68.5 Gbp of raw data, consisting of 7,751,459 ONT long reads (N50=31 kbp) with a quality score of Q20 of 90.1% and Q30 of 81.3%, along with 103 Gbp of proximity-ligated DNA fragments, producing 434,262,093 reads with a mean quality score of 19, facilitated the generation of a complete assembly of the *A. endiviifolia* reference genome. Raw reads were assembled using hifiasm, resulting in 1860 contigs with a total size of 2,956 Mbp and an N50 length of 76.58 Mbp (Table 1). Subsequently, the initial contigs served as the backbone of the scaffold contig in the chromosomes using Pore-C data. After gap filling and polishing, the final assembly had a total size of 2,914,960,273 bp with an N50 of 468,157,909 bp (Table 1), comprising nine chromosomes (with six gaps in intergenic regions) ranging from 100,551,284 to 529,742,643 bp in length (Table 2, Fig. 2 and 3).

Assembly accuracy and completeness were evaluated using multiple methodologies. The Pore-C interaction heatmap demonstrated a high degree of consistency across all chromosomes, thereby providing robust evidence of the precision of genome sequencing (Fig. 2). The BUSCO scores for Viridiplantae_odb10 and Eukaryota_odb10 were 99.2% and 95.5%, respectively, with low gene duplication levels of 6.2% and 4.4%, respectively. Furthermore, integrity

assessments of the LTRs indicated an assembled LTR assembly index (LAI) of 20.06. The genome exhibited a consensus quality value (QV) of 47.6 (Table 1). Collectively, these findings underscore the high accuracy and reliability of the *A. endiviifolia* genome assembly. The assembled genome and gene annotation can be found in the NCBI assembly with submission number JBRAUX000000000.

In the process of annotating the genome of *A. endiviifolia*, short-read RNA sequencing was performed on the thallus of both aquatic (16.8 Gbp) and terrestrial forms (20.6 Gbp), as well as on the antheridia themselves (6 Gbp) and surrounding thallus (6.3 Gbp), generating a total of 49.7 Gbp reads. Genomic annotation identified 34,615 genes, including 33,513 protein-coding genes (Table 1). In the comprehensive analysis of the overall distribution of all significant Gene Ontology (GO) terms, the "Biological Process" category contained the highest number of unique terms, significantly surpassing the other categories with 96 terms, accounting for 45.1% of the total. The "Molecular Function" category ranked second in abundance (66 terms, 31%), while the "Cellular Component" category had the fewest unique GO terms (51 terms, 23.9%), reflecting the relatively limited number of subcellular locations or structures annotated within the genome (Fig. 4). GO enrichment analysis revealed that six terms predominated among the top 15 significantly enriched GO terms across all ontologies: "poly(A)+ mRNA export from nucleus," "ethylene-activated signaling pathway," "double-stranded DNA binding," "protein-containing complex localization," "protein export from nucleus," and "ribonucleoprotein complex localization" (Fig. 4). The remaining categories contain progressively fewer terms. The composition of the TEs included 12.16% LTRs elements and 57 Helitrons. Among the classified retroelements, the *Copia* and *Gypsy* superfamilies accounted for 8.94% and 2.95% of the assembly, respectively (Fig. 3). The phylogenetic tree illustrates the diversity and evolutionary relationships among LTR retrotransposon families identified in the analyzed genome, including major lineages such as *Athila*, *Phygy*, *Tekay*, and others (Fig. 5). The GC content and gene density were assessed in 500 kbp windows across each chromosome. The average GC content across all chromosomes ranged from 38.8% to 39.6%. The highest gene density was observed on chromosome 6 with a value of 9.78, whereas the lowest gene density was recorded on chromosome 9 with a value of 5.52 (Fig. 3).

Detection and characteristics of telomeres and centromeres

The completion and accuracy of genome sequencing have enabled the identification of telomeres and centromeres (Table 2, Fig. 3). Examination of the telomeric regions by scanning

chromosome ends for high-copy tandem repeats showed that both ends of the seven *A. endiviifolia* chromosomes (except chromosomes 3 and 6) feature telomere repeat sequences (CCCTAAA/TTTAGGG) that align with telomeric structures typical of most plant species. For these seven chromosomes, the number of repeats at the left end ranged from 352 to 481, whereas those at the right end ranged from 402 to 452 (Table 2). In chromosomes 3 and 6, the typical telomeric sequence was identifiable at one end, whereas the opposite end exhibited a substantial number of repeats of an alternative motif. Specifically, the left end of chromosome 3 contained 115 repeats of the ACGCAGC motif, whereas the right end of chromosome 6 contained 176 repeats of the TGCGTCG motif (Table 2).

The application of quarTeT, a tool that identifies centromeres by computationally detecting and mapping tandem repeats and associated retrotransposons in assembled genomes, in conjunction with CentIER, which identifies centromeres by clustering tandem repeats (Supplementary Table S1), mapping their abundance and distribution, and designating regions with extensive dense arrays as candidate centromeres, facilitated reliable identification of centromeric regions across all nine chromosomes. However, to confirm the presence of centromeres in chromosomes 4 and 5, it was necessary to verify the results obtained from the quarTeT analysis by comparing them with the gaps observed in the Pore-C interaction heatmap (Supplementary Fig. S1). Furthermore, all sites identified as centromeres on the remaining seven chromosomes were corroborated by the Pore-C interaction heatmap. Examination of these centromeric regions confirmed that LTR/*Gypsy* elements were highly enriched in the immediate vicinity of the centromere and their abundance decreased as the distance from the centromere increased, as presented in detail for chromosome 1 (Fig. 6). Additionally, this analysis demonstrated a correlation between high LTR/*Gypsy* density and formation of a specialized chromatin domain at the centromere. Moreover, LTR domains from the *Ty3/Gypsy* superfamily, particularly those belonging to the *Tat* (*TatI*, *TatII*, and *TatIII*), *Phygy*, and *Selgy* family, were found to be significantly enriched in centromeric regions compared to non-centromeric chromosomal regions. Although LTR elements from the *Bel-Pao* family are infrequently present in centromeres, they nonetheless showed a statistically significant enrichment in these regions relative to the rest of the genome (Fig. 7). Finally, the validated centromeres exhibited length variation ranging from 1.9 to 5 Mbp (Table 2). This comprehensive genomic assembly highlights the effectiveness of long-read sequencing technologies in elucidating complex genomic structures.

Genome synteny analysis

Collinearity between the nine chromosomes of *A. endiviifolia* and the leafy liverwort *Herbertus hutchinsiae*, both belonging to the Jungermanniopsida class, revealed complex and divergent synteny patterns characterized by fragmentation into small syntenic blocks distributed across multiple chromosomes rather than forming extensive chromosome-scale fusions. Details of the detected syntenic blocks between *A. endiviifolia* and *H. hutchinsiae* chromosomes, including chromosome coordinates and strand orientation, are provided in Supplementary Table S2. Only chromosome nine showed a slightly conserved relationship between the two species (Fig. 8). Furthermore, analysis of genomic conservation and structural variations between *A. endiviifolia* and the model liverwort *M. polymorpha* did not reveal significant collinearity (Supplementary Fig. S2).

Phylogenetic relationship analysis

The protein sequences of single-copy orthologs conserved across *A. endiviifolia* and 13 other liverwort species with chromosome-scale genome assemblies were selected for phylogenetic analysis to elucidate evolutionary relationships among these species. The resulting phylogenetic tree clearly separated Marchantiopsida and Jungermanniopsida into distinct evolutionary lineages. Notably, all internal nodes in the tree were supported by 100% bootstrap values, reflecting the maximal confidence in the overall tree topology. This strong support underscores the reliability of the inferred evolutionary relationships. Additionally, the observed variation in genome size, particularly in the large genomes of some Jungermanniopsida, may reflect lineage-specific differences in genome dynamics, such as variation in transposable element activity, gene duplication, and structural rearrangements (Fig. 9).

Re-use potential

The telomere-to-telomere reference genome assembly of the haploid liverwort *A. endiviifolia* is a high-quality genomic resource with a broad potential for reuse across multiple research fields. Generated using Oxford Nanopore long-read sequencing combined with Pore-C technology, this 2,914,960,273 bp assembly achieves chromosome-scale resolution with exceptional completeness (99.2% BUSCO) and accuracy (QV 47.6). It comprises nine experimentally validated chromosomes featuring 18 telomeres and nine predicted centromeres enriched in LTR/*Gypsy* retrotransposons, and includes 34,615 annotated genes. The unusual presence of non-canonical telomeric-like repeats (ACGCAGC and TGCGTCG) on chromosomes 3 and 6 in *A. endiviifolia* suggests species-specific telomere evolution or chromosomal rearrangements, though their biological significance is unclear. Further

comparative and functional studies are needed to understand their origin and role. These findings contribute to the genomic landscape of *A. endiviifolia* and support future research on plant telomere diversity and evolution. Notably, the centromeres have been predicted solely through in silico analyses, representing a plausible but not yet fully validated hypothesis. This comprehensive dataset enables comparative genomics within liverworts and across land plants, facilitating studies of genome evolution, centromere biology, chromosome end structures, genome stability, and chromosome segregation. By providing a complete, well-annotated, and experimentally validated genome, this resource provides a robust foundation for future research in plant genomics, cytogenetics, and evolutionary biology, extending beyond the scope of the current study.

Contributions

J.S.-P. and J.S. contributed to research design, J.S-P. carried out laboratory analyses, J.S. carried out bioinformatic analyses, visualized data and prepared figures, P.S. was responsible for in vitro cultures, J.S.-P. and J.S. analyzed the data and wrote the original draft. J.S. obtained funding. All authors revised and approved the final version of the manuscript.

Funding

This study was financially supported by the National Science Center, Kraków, Poland (Grant No. 2020/39/B/NZ8/02504.

Acknowledgements

We would like to thank Dr. Katarzyna Krawczyk for her assistance with the Pore-C procedure. We also extend our gratitude to the staff of the Department of Botany and Evolutionary Ecology at the University of Warmia and Mazury in Olsztyn for the pleasant working atmosphere.

References

1. Bechteler J, Peñaloza-Bojacá G, Bell D, Gordon Burleigh J, McDaniel SF, Christine Davis E, et al.. Comprehensive phylogenomic time tree of bryophytes reveals deep relationships and uncovers gene incongruences in the last 500 million years of diversification. *Am J Bot*. John Wiley and Sons Inc; 2023; doi: 10.1002/AJB2.16249.

2. Donoghue PCJ, Harrison CJ, Paps J, Schneider H. The evolutionary emergence of land plants. *Current Biology*. Cell Press; 2021; doi: 10.1016/J.CUB.2021.07.038.

3. Harris BJ, Clark JW, Schrempf D, Szöllősi GJ, Donoghue PCJ, Hetherington AM, et al.. Divergent evolutionary trajectories of bryophytes and tracheophytes from a complex common ancestor of land plants. *Nat Ecol Evol*. Nature Research; 2022; doi: 10.1038/S41559-022-01885-X.

4. Linde AM, Singh S, Bowman JL, Eklund M, Cronberg N, Lagercrantz U. Genome Evolution in Plants: Complex Thalloid Liverworts (Marchantiopsida). *Genome Biol Evol*. Oxford University Press; 2023; doi: 10.1093/GBE/EVAD014.

5. Villarreal A. JC, Crandall-Stotler BJ, Hart ML, Long DG, Forrest LL. Divergence times and the evolution of morphological complexity in an early land plant lineage (Marchantiopsida) with a slow molecular rate. *New Phytologist*. Blackwell Publishing Ltd; 2016; doi: 10.1111/NPH.13716.

6. Renner MAM, Heslewood MM, Patzak SDF, Schäfer-Verwimp A, Heinrichs J. By how much do we underestimate species diversity of liverworts using morphological evidence? An example from Australasian Plagiochila (Plagiochilaceae: Jungermanniopsida). *Mol Phylogenet Evol*. Academic Press; 2017; doi: 10.1016/J.YMPEV.2016.12.018.

7. Söderström L, Hagborg A, Von Konrat M, Bartholomew-Began S, Bell D, Briscoe L, et al.. World checklist of hornworts and liverworts. *PhytoKeys 59: 1-828*. Pensoft Publishers; 2016; doi: 10.3897/PHYTOKEYS.59.6261.

8. Paukszto Ł, Górski P, Krawczyk K, Maździarz M, Szczecińska M, Ślipiko M, et al.. The organellar genomes of Pellidae (Marchantiophyta): the evidence of cryptic speciation, conflicting phylogenies and extraordinary reduction of mitogenomes in simple thalloid liverwort lineage. *Sci Rep*. Nature Research; 2023; doi: 10.1038/S41598-023-35269-3.

9. Crandall-Stotler B, Stotler RE, Zhang L, Forrest LL. On the morphology, systematics and phylogeny of Noteroclada (Noterocladaceae, Marchantiophyta). *Nova Hedwigia*. Schweizerbart'sche Verlagsbuchhandlung; 2010; doi: 10.1127/0029-5035/2010/0091-0421.

10. Schütz N, Quandt D, Nebel M. The position of the genus Apopellia stat. Nov. within the Pelliales (Marchantiophytina: Jungermanniopsida). *Taxon*. International Association for Plant Taxonomy; 2016; doi: 10.12705/652.1.

11. Crandall-Stotler B, Stotler RE, Long DG. PHYLOGENY AND CLASSIFICATION OF THE MARCHANTIOPHYTA. *Edinb J Bot*. Cambridge University Press; 2009; doi: 10.1017/S0960428609005393.

12. Sawicki J, Krawczyk K, Ślipiko M, Szandar K, Szczecińska M. Comparative analysis of apopellia endiviifolia plastomes reveals a strikingly high level of differentiation between its terrestrial and water form. *Diversity (Basel)*. MDPI; 2021; doi: 10.3390/D13120674/S1.

13. Interpretation of Electrophoretic Patterns in Population Genetics of Bryophytes: VI. Genetic Variation and Evolution of the Liverwort Genus Pellia with Special Reference to Central European Territory on JSTOR. https://www.jstor.org/stable/20149587?seq=1 Accessed 2025 Jun 25.

14. Pellicer J, Hidalgo O, Dodsworth S, Leitch IJ. Genome Size Diversity and Its Impact on the Evolution of Land Plants. *Genes (Basel)*. MDPI AG; 2018; doi: 10.3390/GENES9020088.

15. Temsch EM, Greilhuber J, Krisai R. Genome size in liverworts. *Preslia*. 82:63–80, 2010.

16. Bainard JD, Forrest LL, Goffinet B, Newmaster SG. Nuclear DNA content variation and evolution in liverworts. *Mol Phylogenet Evol*. Academic Press; 2013; doi: 10.1016/J.YMPEV.2013.04.008.

17. Singh S, Bowman JL. The monoicous secondarily aquatic liverwort Ricciocarpos natans as a model within the radiation of derived Marchantiopsida. *Front Plant Sci*. Frontiers Media SA; 2023; doi: 10.3389/FPLS.2023.1260596/ENDNOTE.

18. Krawczyk K, Szablińska-Piernik J, Paukszto Ł, Maździarz M, Sulima P, Przyborowski JA, et al.. Chromosome-scale telomere to telomere genome assembly of common crystalwort (Riccia sorocarpa Bisch.). *Scientific Data 2025 12:1*. Nature Publishing Group; 2025; doi: 10.1038/s41597-025-04373-6.

19. Levins J, Paukszto Ł, Krawczyk K, Maździarz M, Arch BC, Cargill DC, et al.. Evolution of sexual systems and regressive evolution in Riccia. *New Phytologist*. John Wiley & Sons, Ltd; 2025; doi: 10.1111/NPH.20454.

20. Fu Y, Zhang X, Zhang T, Sun W, Yang W, Shi Y, et al.. Evidence for evolution of a new sex chromosome within the haploid-dominant Marchantiales plant lineage. *J Integr Plant Biol*. John Wiley and Sons Inc; 2025; doi: 10.1111/JIPB.13867/SUPPINFO.

21. Bowman JL, Kohchi T, Yamato KT, Jenkins J, Shu S, Ishizaki K, et al.. Insights into Land Plant Evolution Garnered from the Marchantia polymorpha Genome. *Cell*. Cell Press; 2017; doi: 10.1016/J.CELL.2017.09.030.

22. Linde AM, Eklund DM, Cronberg N, Bowman JL, Lagercrantz U. Rates and patterns of molecular evolution in bryophyte genomes, with focus on complex thalloid liverworts, Marchantiopsida. *Mol Phylogenet Evol*. Academic Press; 2021; doi: 10.1016/J.YMPEV.2021.107295.

23. Miga KH. Centromere studies in the era of ‘telomere-to-telomere’ genomics. *Exp Cell Res*. Elsevier Inc.; 2020; doi: 10.1016/J.YEXCR.2020.112127.

24. Peng D, Hong Z, Kan S, Wu Z, Liao X. The telomere-to-telomere (T2T) genome provides insights into the evolution of specialized centromere sequences in sandalwood. *Gigascience*. 2024; doi: 10.1093/gigascience/giae096.

25. Bi G, Zhao S, Yao J, Wang H, Zhao M, Sun Y, et al.. Near telomere-to-telomere genome of the model plant Physcomitrium patens. *Nature Plants* . Nature Research; 2024; doi: 10.1038/s41477-023-01614-7.

26. Montgomery SA, Tanizawa Y, Galik B, Wang N, Ito T, Mochizuki T, et al.. Chromatin Organization in Early Land Plants Reveals an Ancestral Association between H3K27me3, Transposons, and Constitutive Heterochromatin. *Curr Biol*. Cell Press; 2020; doi: 10.1016/J.CUB.2019.12.015.

27. Sawicki J, Krawczyk K, Kurzyński M, Maździarz M, Paukszto Ł, Sulima P, et al.. Nanopore sequencing of organellar genomes revealed heteroplasmy in simple thalloid and leafy liverworts. *Acta Societatis Botanicorum Poloniae*. Polish Botanical Society; 2023; doi: 10.5586/ASBP/172516.

28. Lu D, Liu C, Ji W, Xia R, Li S, Liu Y, et al.. Nanopore ultra-long sequencing and adaptive sampling spur plant complete telomere-to-telomere genome assembly. *Mol Plant*. Cell Press; 2024; doi: 10.1016/J.MOLP.2024.10.008.

29. Zhong JY, Niu L, Lin Z Bin, Bai X, Chen Y, Luo F, et al.. High-throughput Pore-C reveals the single-allele topology and cell type-specificity of 3D genome folding. *Nature*

Communications 2023 14:1. Nature Publishing Group; 2023; doi: 10.1038/s41467-023-36899-x.
30. GitHub - nanoporetech/dorado: Oxford Nanopore's Basecaller. https://github.com/nanoporetech/dorado Accessed 2025 Sep 10.
31. Liu B, Shi Y, Yuan J, Hu X, Zhang H, Li N, et al.. Estimation of genomic characteristics by analyzing k-mer frequency in de novo genome projects. 2013; doi: 10.48550/arXiv.1308.2012.
32. Wang H, Liu B, Zhang Y, Jiang F, Ren Y, Yin L, et al.. Estimation of genome size using k-mer frequencies from corrected long reads. 2020; doi: 10.48550/arXiv.2003.11817.
33. Cheng H, Concepcion GT, Feng X, Zhang H, Li H. Haplotype-resolved de novo assembly using phased assembly graphs with hifiasm. *Nat Methods*. Nature Research; 2021; doi: 10.1038/S41592-020-01056-5.
34. GitHub - epi2me-labs/wf-pore-c. https://github.com/epi2me-labs/wf-pore-c Accessed 2025 Sep 10.
35. Zhou C, McCarthy SA, Durbin R. YaHS: yet another Hi-C scaffolding tool. *Bioinformatics*. Oxford Academic; 2023; doi: 10.1093/BIOINFORMATICS/BTAC808.
36. Durand NC, Robinson JT, Shamim MS, Machol I, Mesirov JP, Lander ES, et al.. Juicebox Provides a Visualization System for Hi-C Contact Maps with Unlimited Zoom. *Cell Syst*. Cell Press; 2016; doi: 10.1016/j.cels.2015.07.012.
37. GitHub - alekseyzimin/masurca. https://github.com/alekseyzimin/masurca Accessed 2025 Sep 10.
38. Manni M, Berkeley MR, Seppey M, Simão FA, Zdobnov EM. BUSCO Update: Novel and Streamlined Workflows along with Broader and Deeper Phylogenetic Coverage for Scoring of Eukaryotic, Prokaryotic, and Viral Genomes. *Mol Biol Evol*. Oxford University Press; 2021; doi: 10.1093/MOLBEV/MSAB199,.
39. Chen Y, Zhang Y, Wang AY, Gao M, Chong Z. Accurate long-read de novo assembly evaluation with Inspector. *Genome Biol*. BioMed Central Ltd; 2021; doi: 10.1186/S13059-021-02527-4/FIGURES/4.
40. Ou S, Su W, Liao Y, Chougule K, Agda JRA, Hellinga AJ, et al.. Benchmarking transposable element annotation methods for creation of a streamlined, comprehensive pipeline. *Genome Biol*. BioMed Central Ltd.; 2019; doi: 10.1186/S13059-019-1905-Y/FIGURES/6.
41. GitHub - ncbi/egapx: Eukaryotic Genome Annotation Pipeline-External caller scripts and documentation. https://github.com/ncbi/egapx Accessed 2025 Sep 10.
42. Li H. Protein-to-genome alignment with miniprot. *Bioinformatics*. Oxford University Press; 2023; doi: 10.1093/BIOINFORMATICS/BTAD014,.
43. Dobin A, Davis CA, Schlesinger F, Drenkow J, Zaleski C, Jha S, et al.. STAR: Ultrafast universal RNA-seq aligner. *Bioinformatics*. Bioinformatics; 2013; doi: 10.1093/BIOINFORMATICS/BTS635,.
44. Gnomon - the NCBI eukaryotic gene prediction tool. https://www.ncbi.nlm.nih.gov/refseq/annotation_euk/gnomon/ Accessed 2025 Sep 10.
45. Zhang RG, Li GY, Wang XL, Dainat J, Wang ZX, Ou S, et al.. TEsorter: An accurate and fast method to classify LTR-retrotransposons in plant genomes. *Hortic Res*. Oxford University Press; 2022; doi: 10.1093/HR/UHAC017.

46. Katoh K, Standley DM. MAFFT Multiple Sequence Alignment Software Version 7: Improvements in Performance and Usability. *Mol Biol Evol*. Oxford Academic; 2013; doi: 10.1093/MOLBEV/MST010.
47. Minh BQ, Schmidt HA, Chernomor O, Schrempf D, Woodhams MD, Von Haeseler A, et al.. IQ-TREE 2: New Models and Efficient Methods for Phylogenetic Inference in the Genomic Era. *Mol Biol Evol*. Oxford University Press; 2020; doi: 10.1093/MOLBEV/MSAA015,.
48. Lin Y, Ye C, Li X, Chen Q, Wu Y, Zhang F, et al.. quarTeT: a telomere-to-telomere toolkit for gap-free genome assembly and centromeric repeat identification. *Hortic Res*. Oxford Academic; 2023; doi: 10.1093/HR/UHAD127.
49. Xu D, Yang J, Wen H, Feng W, Zhang X, Hui X, et al.. CentIER: Accurate centromere identification for plant genomes. *Plant Commun*. Elsevier; 2024; doi: 10.1016/J.XPLC.2024.101046.
50. GitHub - kubek78/PhylogenomicsME: Phylogenomics Made Easy. https://github.com/kubek78/PhylogenomicsME/tree/main Accessed 2025 Sep 10.
51. Pertea G, Pertea M. GFF Utilities: GffRead and GffCompare. *F1000Res*. NLM (Medline); 2020; doi: 10.12688/F1000RESEARCH.23297.2/DOI.
52. Tang H, Krishnakumar V, Zeng X, Xu Z, Taranto A, Lomas JS, et al.. JCVI: A versatile toolkit for comparative genomics analysis. *iMeta*. John Wiley & Sons, Ltd; 2024; doi: 10.1002/IMT2.211.
53. Emms DM, Kelly S. OrthoFinder: Phylogenetic orthology inference for comparative genomics. *Genome Biol*. BioMed Central Ltd.; 2019; doi: 10.1186/S13059-019-1832-Y.
54. Buchfink B, Reuter K, Drost HG. Sensitive protein alignments at tree-of-life scale using DIAMOND. *Nat Methods*. Nature Research; 2021; doi: 10.1038/S41592-021-01101-X.
55. Coombe L, Kazemi P, Wong J, Birol I, Warren RL. Multi-genome synteny detection using minimizer graph mappings. *bioRxiv*. Cold Spring Harbor Laboratory; 2024; doi: 10.1101/2024.02.07.579356.
56. Hackl T, Ankenbrand M, van Adrichem B, Wilkins D, Haslinger K. gggenomes: effective and versatile visualizations for comparative genomics. 2024;
57. Capella-Gutiérrez S, Silla-Martínez JM, Gabaldón T. trimAl: a tool for automated alignment trimming in large-scale phylogenetic analyses. *Bioinformatics*. Oxford Academic; 2009; doi: 10.1093/BIOINFORMATICS/BTP348.
58. Kalyaanamoorthy S, Minh BQ, Wong TKF, Von Haeseler A, Jermiin LS. ModelFinder: Fast model selection for accurate phylogenetic estimates. *Nat Methods*. Nature Publishing Group; 2017; doi: 10.1038/NMETH.4285.
59. Hoang DT, Chernomor O, Von Haeseler A, Minh BQ, Vinh LS. UFBoot2: Improving the Ultrafast Bootstrap Approximation. *Mol Biol Evol*. Oxford Academic; 2018; doi: 10.1093/MOLBEV/MSX281.
60. Paradis E, Schliep K. ape 5.0: an environment for modern phylogenetics and evolutionary analyses in R. *Bioinformatics*. Oxford Academic; 2019; doi: 10.1093/BIOINFORMATICS/BTY633.
61. Schliep KP. phangorn: phylogenetic analysis in R. *Bioinformatics*. Oxford Academic; 2011; doi: 10.1093/BIOINFORMATICS/BTQ706.

62. Revell LJ. phytools 2.0: an updated R ecosystem for phylogenetic comparative methods (and other things). *PeerJ*. PeerJ Inc.; 2024; doi: 10.7717/PEERJ.16505.

Table 1. Assembly statistics

**Genome assembly statistics**	
Genome size [bp]	2,914,960,273
Genome coverage [median]	47.6
Number of chromosomes	9
Number of telomeres	18
Number of centromeres	9
Contig N50 [bp]	76,579,043
Scaffold N50 [bp]	468,157,909
Number of gaps	6
Number of total/protein-coding genes	34,615/33,513
QV	47.6
LAI	20.06
BUSCO - genome (Eukaryota/Viridiplantae/Embryophyta) [%]	C:99.2/95.5/63.2 [S:93.0/91.1/60.0, D:6.2/4.4/3.2]

Table 2. The identified telomeres and centromeres in *A. endiviifolia* assembly

Chromosomes	Length [bp]	Telomeres		Centromeres	
		Number of repeats at the left end	Number of repeats at the right end	Start	End
Ch1	529,742,643	425	418	359,400,001	363,400,000
Ch2	473,504,363	352	422	232,800,001	237,800,000
Ch3	468,157,909	115*	414	446,400,001	449,400,000
Ch4	317,755,521	408	402	239,340,762**	241,950,761**
Ch5	310,129,949	481	421	37,548,478**	39,527,839**
Ch6	244,886,949	419	176*	242,160,000	244,159,999
Ch7	236,110,306	393	452	189,670,000	191,669,999
Ch8	234,121,349	428	403	202,730,000	204,729,999
Ch9	100,551,284	446	420	89,933,334	93,766,667

* - telomeres with a non-canonical ACGCAGC/TGCGTCG motif

** - centromeres verification based only on quarTeT and Pore-C contact map

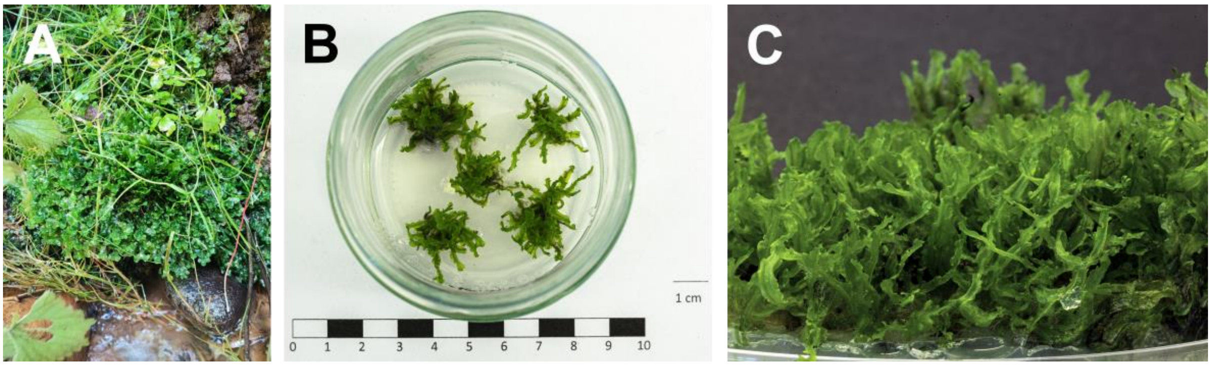

Figure 1. *A. endiviifolia* plants located in the Nature Reserve of the Sources of the Łyna River (A), *in vitro* culture (B), and thalli morphology under *in vitro* conditions (C).

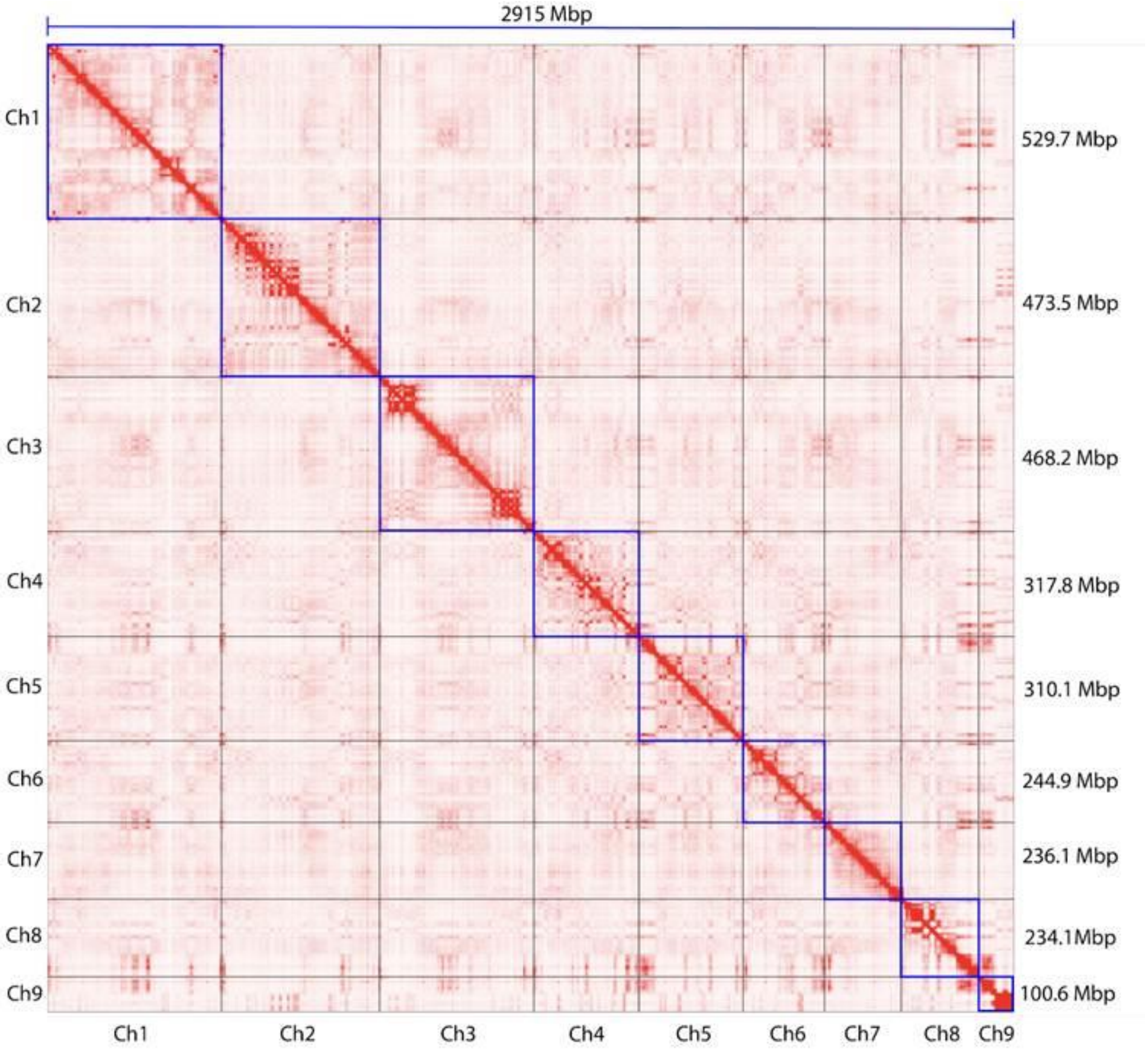

Figure 2. Pore-C interaction heatmap of the *A. endiviifolia* genome.

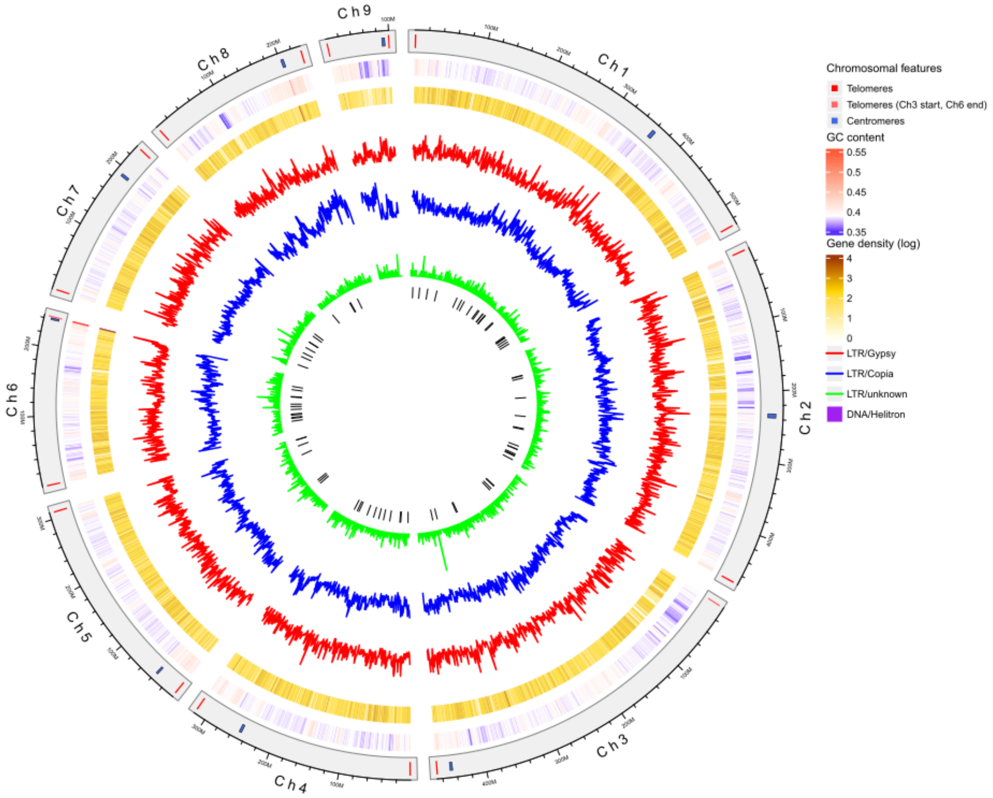

Figure 3. Circos plot showing detailed characterization of the nine *A. endiviifolia* chromosomes. From outside to inside: length of chromosomes in Mbp with marked telomeric (red bars) and centromeric regions (blue bars), GC content, gene density, distribution of LTR/*Gypsy* transposons, distribution of LTR/*Copia* transposons, distribution of LTR/unknown transposons, DNA/Helitron locations.

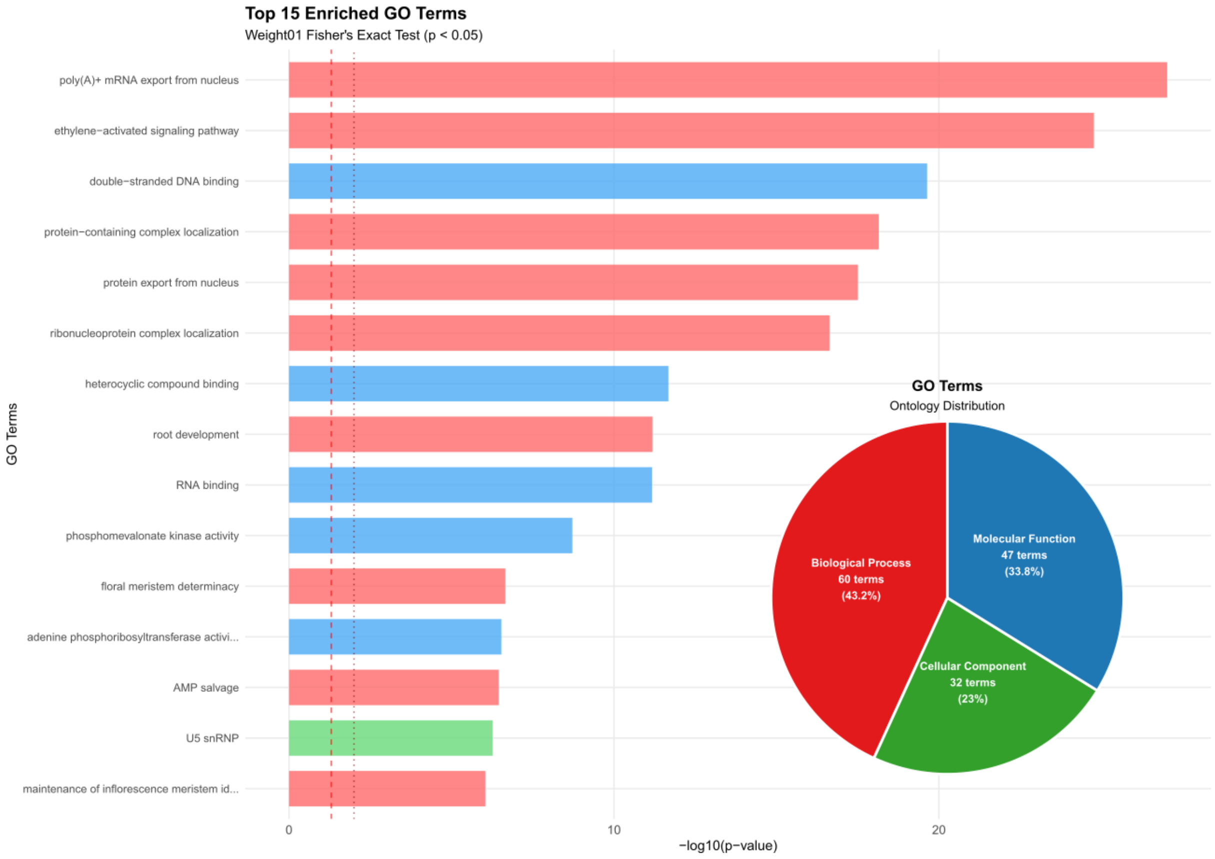

Figure 4. Distribution of all significant GO terms in the genome and the top 15 enriched GO terms across all ontologies in the genome.

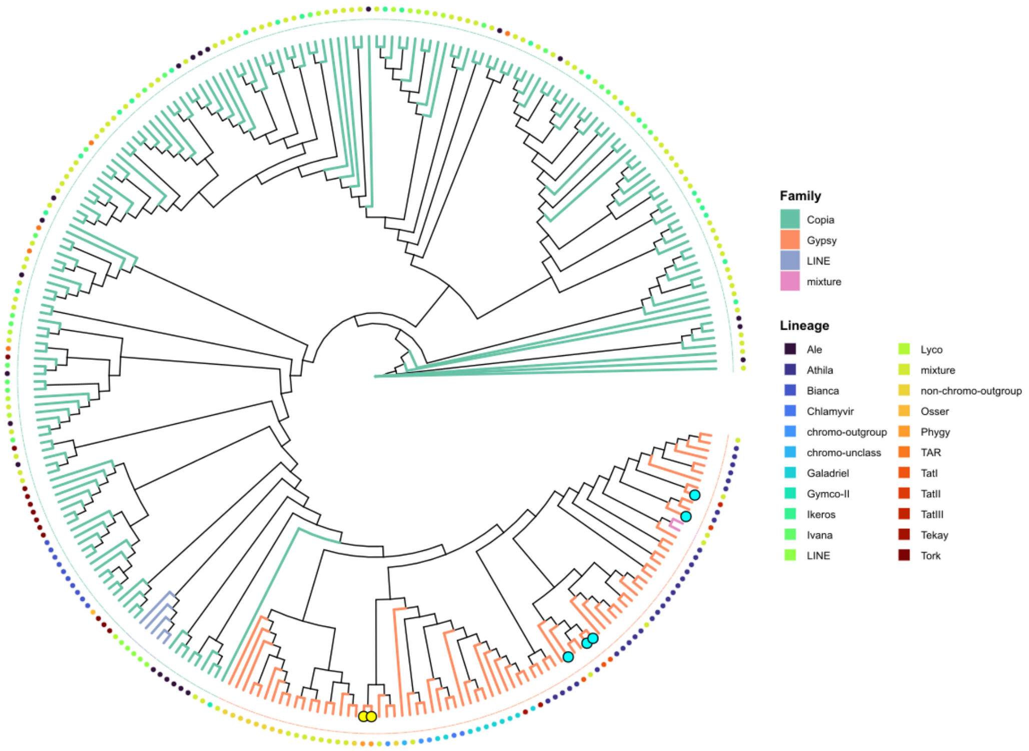

Figure 5. Maximum likelihood phylogenetic tree of LTR retrotransposon lineages identified in the analyzed genome. The tips highlighted in color represent LTR elements enriched in the centromeric regions.

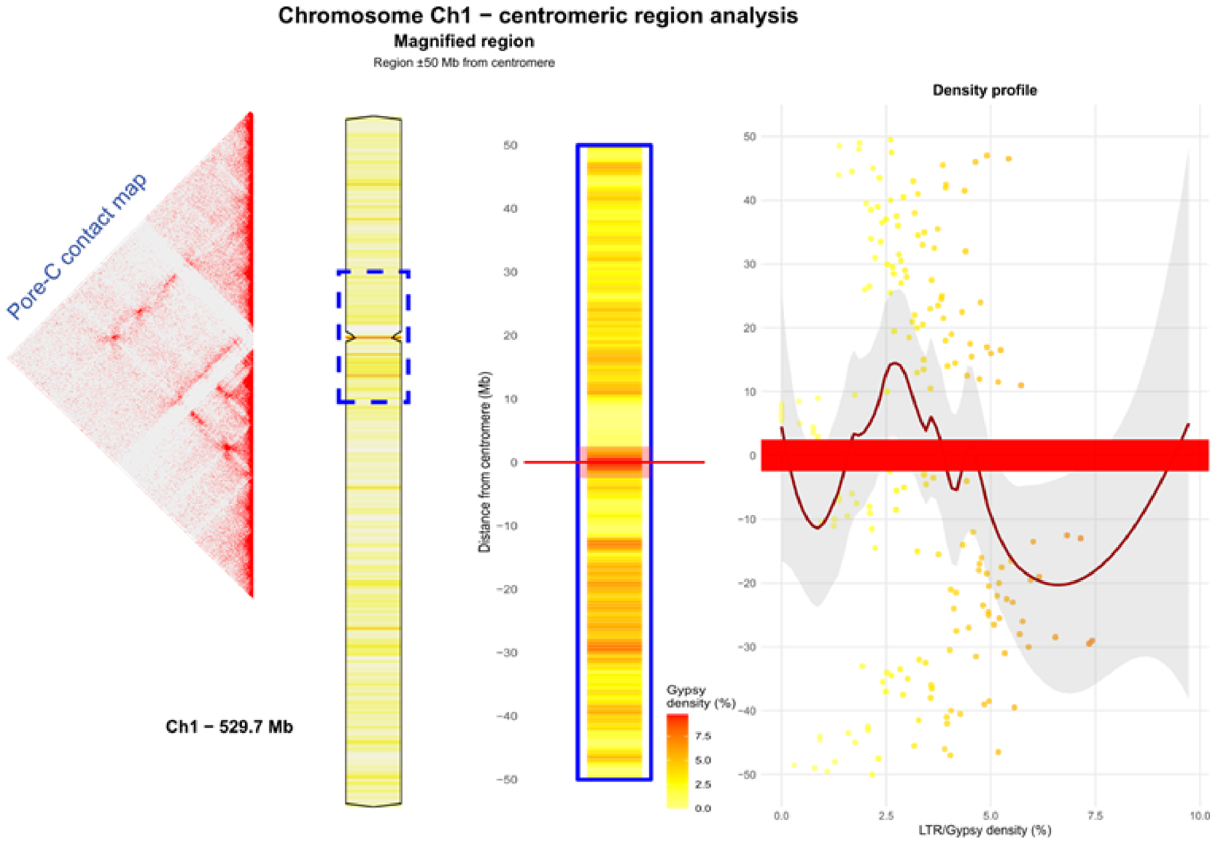

Figure 6. Detailed examination of the example centromere – Analysis of *Gypsy* retrotransposon distribution and chromatin interactions across chromosome 1. In the density profile, each dot represents a single genomic window, plotting its LTR/Gypsy density (X-axis) against its chromosomal position (Y-axis). The solid dark red line shows the overall trend in density, calculated using a LOESS regression, while the surrounding light grey shaded area indicates the 95% confidence interval for this trend. The thick red horizontal bar marks the position of the centromere across the center of the magnified plots.

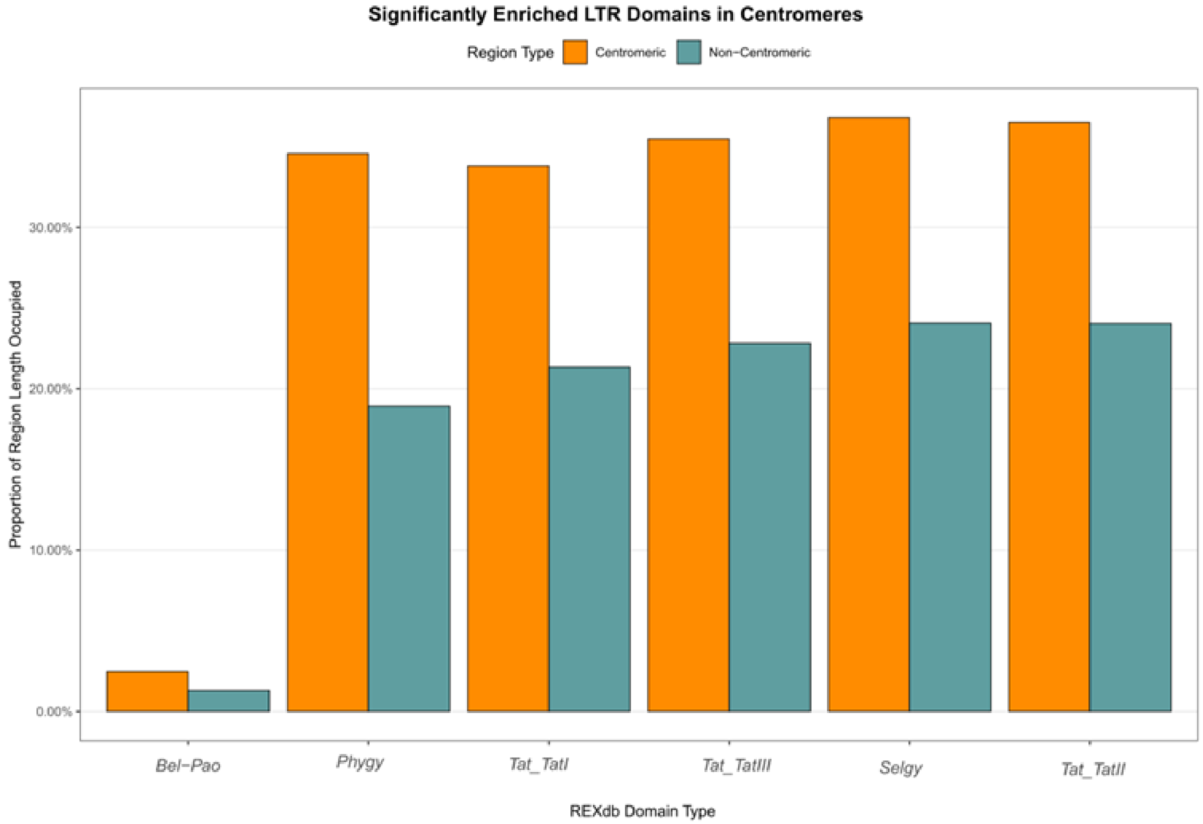

Figure 7. The proportion of regional length occupied by the top six significantly ($p<0.05$) enriched LTR domains in centromeric regions compared with non-centromeric regions.

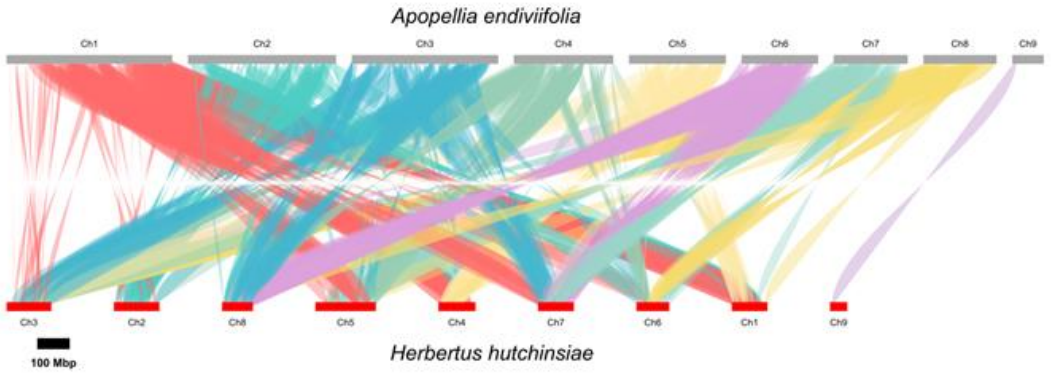

Figure 8. Synteny plot of the differences and similarities between the assembly of *A. endiviifolia* chromosomes and that of *H. hutchinsiae*. Colored lines connect collinear regions between the species' genomes.

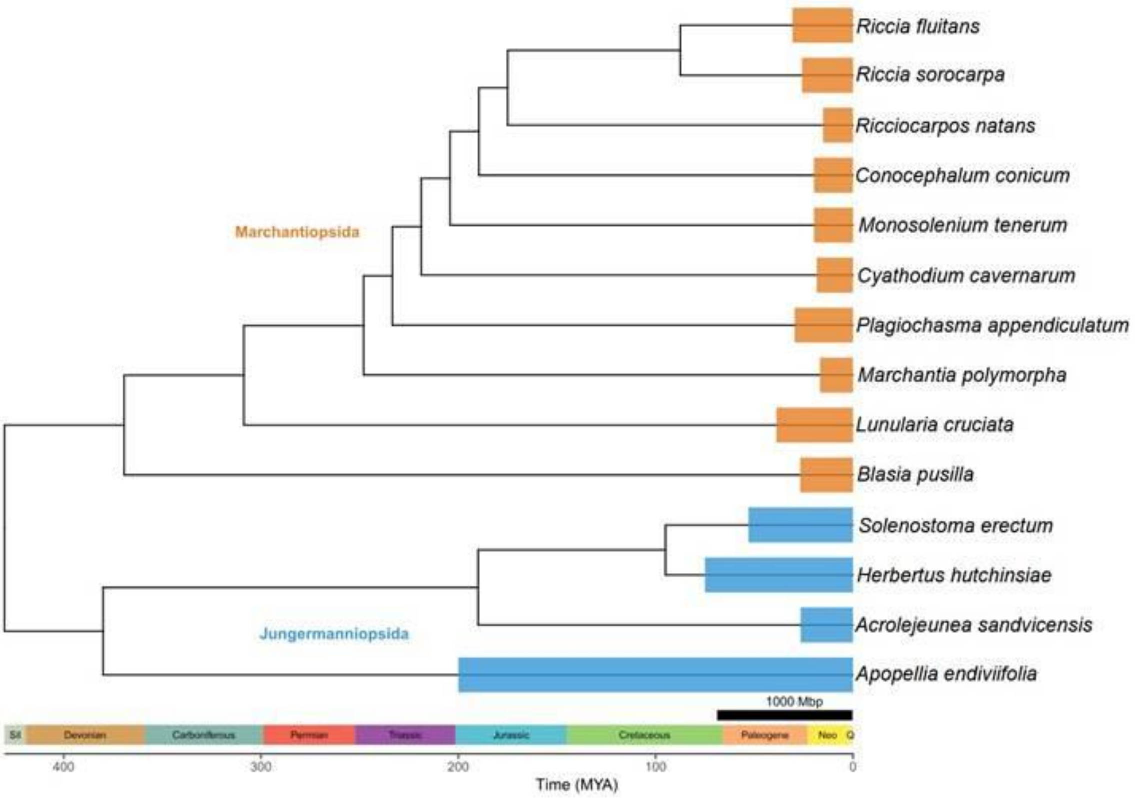

Figure 9. Phylogenetic relationships among liverworts determined by conserved single-copy orthologs. Size of orange and blue boxes corresponds to genome size variations in liverwort species. All nodes have the maximum statistical support.

Supplementary Table S1. Tandem centromeric repeats of *in silico* validated centromeric regions in *A. endiviifolia* assembly. Centromeres in chromosomes 1-3 and 6-9 were identified by the Centier program, and centromeres in chromosomes 4 and 5 were identified by the quarTeT program. All centromeres were validated using a Pore-C interaction heatmap.

Chromosomes	Tandem centromeric repeats
Ch1	CCCTAAAATAGTTTGCTGCTTCTTATGACATTCATTGTTTTGATCAACATAGCAACACGAGAGAAGGTTTATAATGAATCCTGCCA
Ch2	AATTGAAATCATAAATGAAAACCAG
Ch3	ACAACAACATGAGAGAAGGTTTATAATGAATCCTGCCACCCTAAAATCATTTGCTGCTTCTTATGAAATCATTGTTTTGATCGAC
Ch4	GAGGAGTGGTAACCTGAGTATGCAGGTCACCAGTGACCTAAACTATAGGTCACTACCTACTTCACACCTACCTCAAGTCATATCGCCTACCTCAAACCACATGAACCATGTGGTTCCTAACACATGCATCATGTGTTAGTACTTAGTACTAACTAATCATTTAGGCTAAGCTAGAGCTTGGAGTATTAACA
Ch5	TCACCGATGACCTGATCTATAGGTCACTACCTACTTCACACCTACCTCAAGTCATATGACCTACCTCAAACCTCATGAACCATGTGGTTCCTAACACATACATCATGTGTTAGTACTTAGTCCTAATTAATCATTTAGGCTAAGCTAGAACTTGGAGTATTAACAGTTTCTTCCACCAGAACTCGGAGGGTGA
Ch6	GGTTGTGAGCCACTTTTAACCGCGCGGAGTTGATCCCGGGTACAAATTTCCCCAGAAGTATATATGAGGGGGAGGGGTTCGGCGTCTCGGTCCAGGCGCACGGAACGGACCGGCCTCGGGTCTCTTCGGTTGGTCCGGCTCGGTG
Ch7	AAATCATTTGCTGCTTCTTATGAAATCATTGTTTTGATCGACACAGCACCATGAGAGAAGGTTTATAATGAATCCTGTCACCCTAAAATCATTTGCTGCTTCTTATGAAATCATTGTTTTGATCGACACAGCACCATGAGAGAAGGTTTATAATGAATCCTGTCACCCTAAAATCATTTGCTGCTTCTTATGAAATCATTGTTTTGATCGACACAGCACCATGAGAGAAGGTTTATAATGAATCCTGTCACCCTA
Ch8	CGGGTTGTGAGCCACTCTTAACCGCGCGGAGTTGATCCCGGGTACAAATTTCCCCAGAAGTATATATGAGGGGGAGGCGTTCGGCGTCTCGGTCCAGGCGCACGGAACGGACCGGCCTCGGGTCTCGGCTTGTGCTCGGTTGAGG
Ch9	TCTCTCTCTCTCTCTCTCTCTCTCTCTCCCTGCCATCGCATCTCGAGCTGATCAGAGCTCGACCTCGTCTTCTCTCTCTCTCTCTCTCTCTCTCTCTCTCTCTCTCTGCCATCGCATTTCGAGCTGATCAGAGTTCGACCTCGTCCT

Supplementary figures

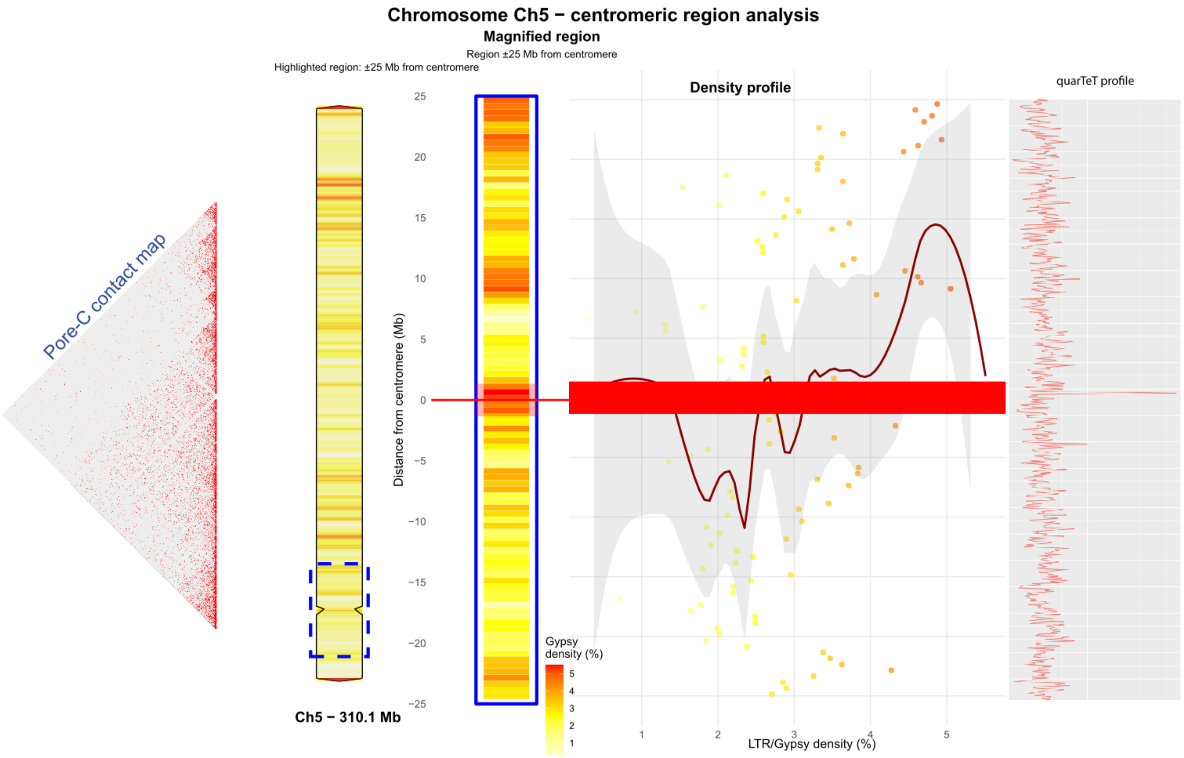

Figure S1. Detailed examination of the predicted centromere of chromosome 5 – Analysis of *Gypsy* retrotransposon distribution and chromatin interactions across chromosome 5. In the density profile, each dot represents a single genomic window, plotting its LTR/Gypsy density (X-axis) against its chromosomal position (Y-axis). The solid dark red line shows the overall trend in density, calculated using a LOESS regression, while the surrounding light grey shaded area indicates the 95% confidence interval for this trend. The thick red horizontal bar marks the position of the centromere across the center of the magnified plots.

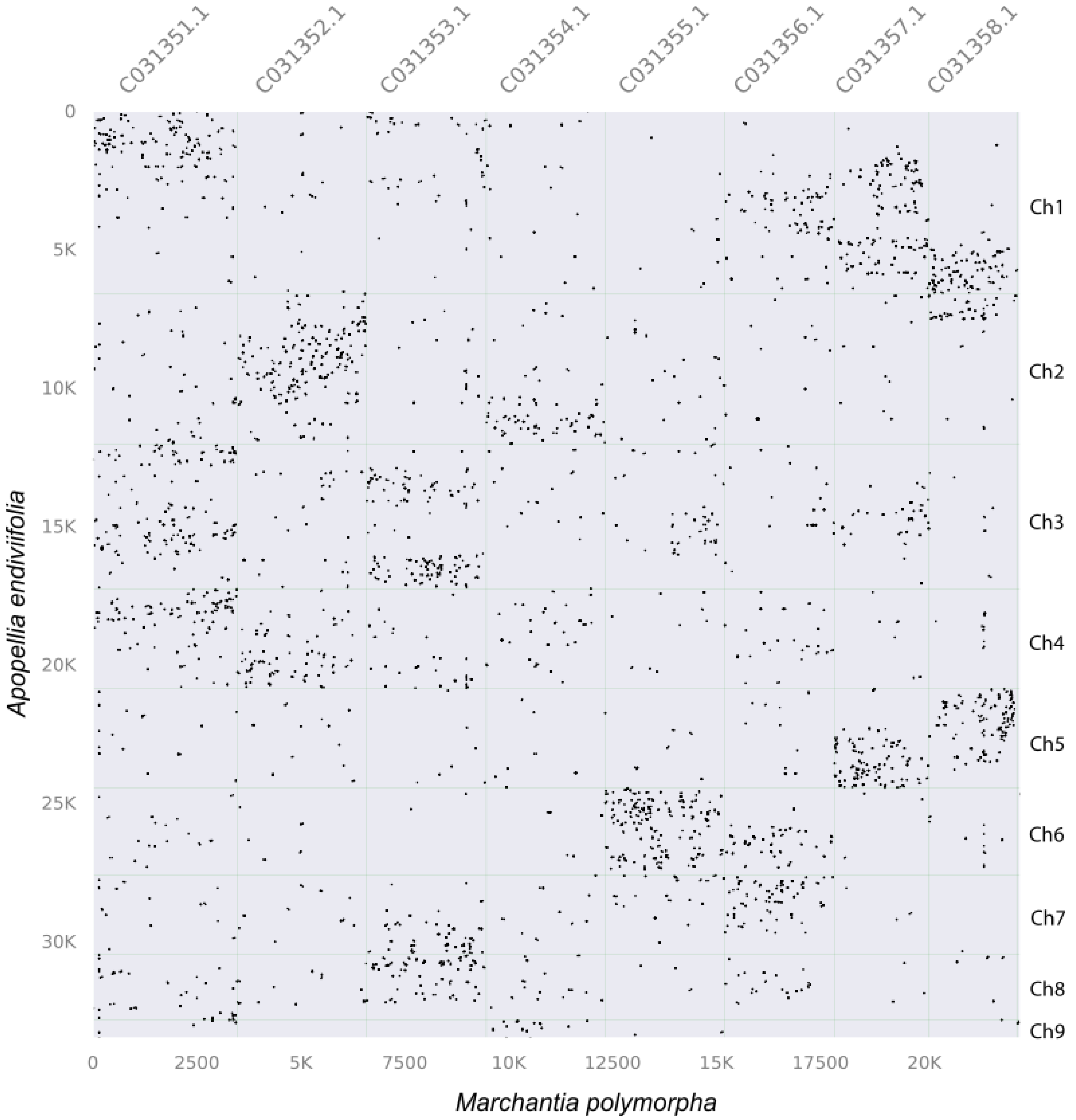

Figure S2. Synteny dot plot comparing the genomes of *M. polymorpha* and *A. endiviifolia*.

Click here to access/download
Supplementary Material
Supplementary Table S2.xlsx

Personal Cover Click here to access/download;Personal Cover;Cover letter.docx

Dear Editors of GigaScience,

We are pleased to submit our manuscript entitled "Giant chromosomes of tiny plant - the complete telomere-to-telomere genome assembly of the simple thalloid liverwort *Apopellia endiviifolia* (Jungermanniopsida, Marchantiophyta)" for consideration for publication in GigaScience.

Our study presents the first high-quality chromosome-level genome assembly of the haploid liverwort *A. endiviifolia*. This species is a key model for understanding cryptic diversity, genomic innovation, and the evolutionary mechanisms underlying speciation in liverworts. The comprehensive genomic resources and analyses we offer, encompassing genome assembly and annotation, as well as the detection and characterization of telomeres and centromeres, will be of significant interest to the genomics, evolutionary biology, and plant science communities.

We confirm that our manuscript fully adheres to the policies of GigaScience. All authors have reviewed and approved the final version of the manuscript and consented to its submission to GigaScience and declare that there are no competing interests related to this work. Furthermore, we confirm that the content of this manuscript has not been previously published, nor is it under consideration for publication elsewhere. We are confident that our manuscript is of high quality and a good fit for your journal. Thank you for your consideration.

Sincerely

Joanna Szablińska-Piernik

Jakub Sawicki
