# Supplementary material for: Giant chromosomes of a tiny plant—the complete telomere-to-telomere genome assembly of the simple thalloid liverwort Apopellia endiviifolia (Jungermanniopsida, Marchantiophyta)
Source: Gigascience. 2025 Nov 29;15:giaf145. doi: 10.1093/gigascience/giaf145 (PMC12885004; doi:10.1093/gigascience/giaf145)
Supplement: giaf145_GIGA-D-25-00252_Revision_2 [file giaf145_giga-d-25-00252_revision_2.pdf]

GigaScience

Giant chromosomes of a tiny plant - the complete telomere-to-telomere genome assembly of the simple thalloid liverwort Apopellia endiviifolia (Jungermanniopsida, Marchantiophyta)

--Manuscript Draft--

**Manuscript Number:**	GIGA-D-25-00252R2
**Full Title:**	Giant chromosomes of a tiny plant - the complete telomere-to-telomere genome assembly of the simple thalloid liverwort Apopellia endiviifolia (Jungermanniopsida, Marchantiophyta)
**Article Type:**	Data Note
**Funding Information:**	Narodowe Centrum Nauki (2020/39/B/NZ8/02504) — Prof. dr hab. Jakub Sawicki
**Abstract:**	Background<br>The liverwort A. endiviifolia, a dioicous, simple thalloid species, is notable for its cryptic diversity, habitat adaptability, genomic innovation, and represents a clade that is a sister to all other Jungermanniopsida. These features make A. endiviifolia an essential model for exploring speciation mechanisms and the evolution of genome structures within liverworts.<br>Findings<br>We present the genome assembly of haploid A. endiviifolia isolate with a total size of 2,914,960,273 bp and an N50 of 468,157,909 bp, demonstrating high completeness (99.2% BUSCO) and a high consensus quality (QV 47.6). The assembly consisted of nine chromosomes, which included validated 18 telomeres and nine centromeres (ranging from 1.9 to 5 Mbp in length). RNA-seq-based annotation identified 34,615 genes, predominantly protein-coding. The TEs comprised 12.16% LTR elements and 57 Helitrons. Among the retroelements, the Copia and Gypsy superfamilies comprised 8.94% and 2.95% of the genome, respectively. The Ty3/Gypsy superfamily was found to be significantly enriched in centromeric regions. The average GC content ranged from 38.8% to 39.6%, with gene density varied between a value 5.52 and 9.78 genes per 500 kbp. Synteny analysis of related liverwort species has revealed complex chromosomal relationships, indicating extensive genome rearrangements among species.<br>Conclusions<br><br>This study provides the first high-quality reference genome assembly of the haploid liverwort A. endiviifolia. Assembly and annotation offers valuable resources for investigating liverwort evolution, centromere biology, and genome expansion in simple thalloid liverworts.
**Corresponding Author:**	Joanna Szablińska-Piernik<br>University of Warmia and Mazury in Olsztyn: Uniwersytet Warminsko-Mazurski w Olsztynie<br>Olsztyn, POLAND
**Corresponding Author Secondary Information:**	
**Corresponding Author's Institution:**	University of Warmia and Mazury in Olsztyn: Uniwersytet Warminsko-Mazurski w Olsztynie
**Corresponding Author's Secondary Institution:**	
**First Author:**	Joanna Szablińska-Piernik
**First Author Secondary Information:**	
**Order of Authors:**	Joanna Szablińska-Piernik
	Paweł Sulima
	Jakub Sawicki

Powered by Editorial Manager® and ProduXion Manager® from Aries Systems Corporation

Order of Authors Secondary Information:	
Response to Reviewers:	We would like to thank the Reviewers for further valuable suggestions on how to improve the manuscript. Below are answers to each comment.<br><br><br>Reviewer reports:<br>Reviewer #1: Overall, the authors satisfactorily addressed most of my major concerns. However, I still feel that the current version of the manuscript needs a second round of revision, as it contains awkward and difficult-to-understand sentences. My detailed comments are provided below.<br><br>L20 Please, revise: «that is sister to all other» rather than "that is a sister to all other".<br>L24 Please, revise: "of a haploid" and not "of haploid".<br>L27 Please rather write "18 validated". But I am not sure that the ms included validation. Centromeres were not cytogenetically validated, only using bioinformatic methods. I would rather say, 18 telomeres.<br>L29 Please, revise: write "57%" instead of "57".<br>L48 Please, revise: instead "framework" use "relationship" or "backbone".<br>L49-53 Please, revise: What is this sentence want to say? It is difficult to follow.<br>L79 Please, revise: rather "evolution of genomic structure" than "structural genomic evolution".<br>L85 rather "while liverwort genomes" than "and liverwqort genomes".<br>L93 Please, revise: awkward sentence….revise for instance "Although liverwort genome sizes are less variable than those of Angiosperms"….<br>L93-96 Please, revise: The sentence is awkwardly formulated and must be revised.<br>L99 Please, add a publication referring to the statement of rare occurrence of TE bursts.<br>L99 I would start the second sentence with "Nevertheless/However a comparative….." because it challenges the statement made in the previous sentence.<br>L105-109 I understand what you want to say but the sentence is overly complicated. Please, revise it.<br>L111 Instead of "in the field of bryophyte research" write "in bryophytes".<br>L116 What is a multi-method study? Don't use this word.<br>L141 Delete "a" in "under a 16h……".<br>L193 I would rather write "For antheridia and surrounding thallus, the amount of extracted RNA was insufficient for TruSeq, libraries were prepared using the QIAseq FX Single Cell RNA Library Kit (Qiagen)".<br>L361-362 Awkward sentence, please, revise it.<br>L385 I would delete this interpretation "reflecting the relatively limited number of subcellular locations or structures annotated within the genome". It makes not too much sense to interpret why one ontology had more or less unique GO terms as this result mainly depends on the extent of homology between the annotated genome and the homologous gene set from which the GO terms were transferred.<br>L392 57% and not 57.<br>Fig9 caption: Revise sentence "Size of orange and blue boxes corresponds to genome size variations in liverwort species" for instance "Size of orange and blue boxes is proportional to the genome size of the species ".<br><br>Answer: All errors and awkward sentences mentioned above have been corrected in accordance with the comments, also the citation has been completed.<br>Reviewer #2: The authors have provided generally thorough and constructive replies to my major and minor comments, and I appreciate the efforts made to improve the manuscript. While most issues were adequately addressed, the synteny and centromere size comparisons still feel somewhat underdeveloped. Even within the constraints of a Data Note, providing a brief numerical or comparative statement (e.g., relative size ranges across liverwort genomes) would make this section more informative to the reader.<br><br>Answer: In response to the review, we have added the following information:<br>- regarding centromeres: “Compared to A. endiviifolia, Marchantia polymorpha centromeres are similarly structured with abundant retroelements and are typically centrally located, ranging from about 176 to 604 Kbp [63]. Physcomitrium patens centromeres share this organization but tend to be smaller, varying from 64 to 221 Kbp, likely reflecting its more compact genome [25]. These differences highlight

	centromere size and composition diversity within bryophytes and demonstrate the value of long-read sequencing for resolving complex genomic regions."<br>- regarding genome synteny analysis: "A total of 429 syntenic blocks spanning approximately 4.8 Mb were detected, with a median size of about 5.2 kb and a mean size of 11.2 kb, indicating small, fragmented conserved regions. These blocks were evenly distributed in terms of strand orientation, with 201 blocks on the positive strand and 228 on the negative strand." ….. "Structural variation analysis revealed numerous chromosomal breakpoints across chromosomes, predominantly in chromosome 1 (54 translocation and 18 inversion breakpoints), while chromosome 9 showed no breakpoints, consistent with its slightly more conserved relationship."<br>Supplementary Table S3 has been added.
**Additional Information:**	
**Question**	**Response**
Are you submitting this manuscript to a special series or article collection?	No
**Experimental design and statistics**<br><br>Full details of the experimental design and statistical methods used should be given in the Methods section, as detailed in our Minimum Standards Reporting Checklist. Information essential to interpreting the data presented should be made available in the figure legends.<br><br>Have you included all the information requested in your manuscript?	Yes
**Resources**<br><br>A description of all resources used, including antibodies, cell lines, animals and software tools, with enough information to allow them to be uniquely identified, should be included in the Methods section. Authors are strongly encouraged to cite Research Resource Identifiers (RRIDs) for antibodies, model organisms and tools, where possible.<br><br>Have you included the information requested as detailed in our Minimum Standards Reporting Checklist?	Yes
**Availability of data and materials**<br><br>All datasets and code on which the	Yes

conclusions of the paper rely must be either included in your submission or deposited in publicly available repositories (where available and ethically appropriate), referencing such data using a unique identifier in the references and in the “Availability of Data and Materials” section of your manuscript.<br><br>Have you have met the above requirement as detailed in our Minimum Standards Reporting Checklist?	
GigaScience has policies and guidelines in place for the use of generative AI-writing tools such as ChatGPT. If you have used such writing tools to assist with writing the manuscript this must be declared and cited in the text. Authors should not list AI-writing tools and other AI-assisted technologies as an author or co-author and should acknowledge that they are fully responsible for text generated or refined by AI-writing tools.<p><br><br>A summary of use (particularly in the introduction or among methods) needs to be included at the end of the paper, and the outputs should also be included as a supplementary file hosted in GigaDB or other open repositories. Please read our guidelines for more information. <p><br><br>By submitting to GigaScience, you are aware of the journal's AI-writing tools policy, and if you have declared use of such tools below, you have acknowledged this where appropriate in your manuscript and have made a summary of use and outputs available. <p><br><b>AI-assisted writing tools have been used in the preparation of this manuscript?	No

Manuscript

Click here to access/download;Manuscript;R1_Szablinska_Apopelia_genom

Giant chromosomes of a tiny plant - the complete telomere-to-telomere genome assembly of the simple thalloid liverwort *Apopellia endiviifolia* (Jungermanniopsida, Marchantiophyta)

Joanna Szablińska-Piernik[1,*], Paweł Sulima[2], Jakub Sawicki[1,*]

1. Department of Botany and Evolutionary Ecology, University of Warmia and Mazury in Olsztyn, Plac Łódzki 1, Olsztyn, 10-719, Poland
2. Department of Genetics, Plant Breeding and Bioresource Engineering, University of Warmia and Mazury in Olsztyn, Plac Łódzki 3, Olsztyn, 10-724, Poland

* corresponding authors: joanna.szablinska@uwm.edu.pl, jakub.sawicki@uwm.edu.pl

Keywords: *Apopellia endiviifolia*, liverworts, telomere-to-telomere [T2T], genome assembly, centromere

Joanna Szablińska-Piernik [0000-0003-0265-940X]
Paweł Sulima [0000-0002-8290-8180]
Jakub Sawicki [0000-0002-4759-8113]

Abstract

Background

The liverwort *A. endiviifolia*, a dioicous, simple thalloid species, is notable for its cryptic diversity, habitat adaptability, genomic innovation, and represents a clade that is sister to all other Jungermanniopsida. These features make *A. endiviifolia* an essential model for exploring speciation mechanisms and the evolution of genome structures within liverworts.

Findings

We present the genome assembly of a haploid *A. endiviifolia* isolate with a total size of 2,914,960,273 bp and an N50 of 468,157,909 bp, demonstrating high completeness (99.2% BUSCO) and a high consensus quality (QV 47.6). The assembly consisted of nine chromosomes, which included 18 telomeres and nine centromeres (ranging from 1.9 to 5 Mbp in length). RNA-seq-based annotation identified 34,615 genes, predominantly protein-coding. The TEs comprised 12.16% LTR elements and 57 Helitrons. Among the retroelements, the *Copia* and *Gypsy* superfamilies comprised 8.94% and 2.95% of the genome, respectively. The Ty3/*Gypsy* superfamily was found to be significantly enriched in centromeric regions. The average GC content ranged from 38.8% to 39.6%, with gene density varied between a value

5.52 and 9.78 genes per 500 kbp. Synteny analysis of related liverwort species has revealed complex chromosomal relationships, indicating extensive genome rearrangements among species.

Conclusions

This study provides the first high-quality reference genome assembly of the haploid liverwort *A. endiviifolia*. Assembly and annotation offer valuable resources for investigating liverwort evolution, centromere biology, and genome expansion in simple thalloid liverworts.

Background

Liverworts (Marchantiophyta) represent one of the earliest terrestrial plants, with fossil evidence indicating their emergence in the Middle Ordovician period approximately 419-447 million years ago [1]. They occupy a pivotal position in plant evolutionary history as members of the monophyletic bryophyte lineage, which, along with vascular plants, form two distinct monophyletic groups within terrestrial plants. Within bryophytes, liverworts are sister to mosses, forming the Setaphyta clade, which in turn is sister to hornworts. This phylogenetic relationship is supported by recent genomic analyses that affirm the monophyly of bryophytes and clarify the evolutionary relationships among early land plants [2]. Liverworts, together with other bryophytes such as mosses and hornworts, display a range of morphological and physiological traits important for plant adaptation to terrestrial environments. However, it remains debated whether these traits are ancestral characteristics or the result of secondary reductions. Recent evidence tends to support the idea of secondary reductions [3]. This group is often characterized by structural simplicity, a predominantly haploid life cycle, and a slow rate of molecular evolution, which corresponds to gradual morphological diversification [4]. However, such generalizations primarily reflect the characteristics of the Haplomitriopsida and Marchantiopsida classes (complex thalloid liverworts) [5]. Significant variation exists within the broader liverwort lineage, particularly in Jungermanniopsida, which includes leafy and simple thalloid liverworts. This class comprises the vast majority (>80%) of extant liverwort species, and demonstrates a significantly higher degree of structural complexity and a notably accelerated rate of molecular evolution. This is evidenced by their diverse leafy morphologies, intricate branching patterns, and specialized reproductive structures, which facilitate their adaptation to a wide array of ecological niches [6–8]. Within Jungermanniopsida, the order Pelliales consists of two families: *Noterocladaceae* and *Pelliaceae* [9]. Recent molecular and morphological studies have refined the taxonomy within *Pelliaceae*, distinguishing *Apopellia*

as a separate genus that includes three species: *A. apicola*, *A. megaspora*, and *A. endiviifolia* [10].

A. endiviifolia (NCBI:txid:304445) is a dioicous, simple thalloid liverwort characterized by its cuneate apical cell, the absence of a midrib in the thallus, a spherical capsule, and a robust seta. It is widely distributed across the Northern Hemisphere and thrives in a diverse array of habitats, including aquatic environments such as springs and streambanks, as well as in dry conditions often associated with limestone substrates. Its capacity to grow on limestone rocks, arid soils, aquatic habitats (hydrophytes), and decaying wood (epixyl) underscores its remarkable ecological versatility [11]. This species exemplifies cryptic speciation, with European populations diverging into two lineages: A (typical form) and B (water form), which are differentiated by molecular markers and microhabitat preferences [8,10,12,13]. This pattern reflects broader taxonomic revisions within *Pellia* s.l., which have been split into *Apopellia* and *Pellia* s.s. through integrative approaches, highlighting its importance in studying speciation mechanisms [10]. The combination of cryptic diversity, habitat versatility, and genomic novelty renders *A. endiviifolia* a valuable model for exploring speciation mechanisms and evolution of genomic structure in liverworts.

Bryophytes, including liverworts, mosses, and hornworts, are generally characterized by relatively small nuclear genomes compared to other plant groups. However, significant variation exists within these groups, particularly among liverworts. On average, hornwort genomes measure 244 Mbp (median 205 Mbp), moss genomes average around 504 Mbp (median 433 Mbp), while liverwort genomes tend to be larger, averaging 1,844 Mbp with a median of 751 Mbp [14]. Flow cytometry data further reveal that liverwort genome sizes vary widely, ranging from 206.2 Mbp in *Lejeunea cavifolia* to 20,006 Mbp in *Phyllothallia fuegiana* [15,16]. Within liverworts, the *Pelliaceae* family is notable for its particularly large genomes: *Pellia borealis* has a genome size of 7,238.3 Mbp, *P. epiphylla* 3,719.2 Mbp, and *A. endiviifolia* 3,364.0 Mbp [15]. Despite efforts to determine the nuclear genome size of over 100 liverwort species, comprehensive genomic resources remain limited, with only a handful of liverwort genomes sequenced at the chromosomal level [17–21].

Although liverwort genome sizes are less variable than those of angiosperms [22], there is still substantial variation in genome size among liverwort species. This variation presents an intriguing area of research, especially given the limited understanding of the patterns and rates of structural changes within liverwort genomes [16]. Studies of genome evolution, particularly in complex thalloid liverworts, have revealed the absence of ancient whole-genome duplication events, minimal rates of gene duplication and chromosomal rearrangements, and rare

occurrences of transposable element (TE) bursts [4]. Nevertheless, a comparative analysis of the nuclear genomes of the model liverwort *M. polymorpha* (286.7 Mbp) and *Lunularia cruciata*, which is distinguished by a genome size nearly twice as large (565.6 Mbp), highlight the role of *Ty3/Gypsy* retrotransposon proliferation in genome size expansion [4]. Furthermore, recent advances in long-read sequencing technologies have enabled the assembly of entire genomes at the telomere-to-telomere scale, providing unprecedented insights into highly repetitive regions such as centromeres and telomeres [23]. These advances have shown that centromere structure is linked to chromosome length evolution. In particular, chromosomes with longer centromeres tend to have a higher proportion and longer stretches of Copia transposable elements within their centromeric regions. This enrichment of *Copia* elements may contribute to a positive association between centromere length and overall chromosome length, potentially influencing karyotype evolution [24]. In bryophytes, the application of near telomere-to-telomere genome assembly has facilitated the identification of centromere sequences in the moss species, *Physcomitrium patens*. This has enabled the precise characterization of its centromeres, revealing 26 monocentric chromosomes, each containing a single centromeric region enriched with RLC5 retrotransposons from the *Bryco* clade of the *Copia* superfamily [25]. Moreover, this study elucidated the evolutionary dynamics of centromeres in non-seed plants, highlighting their unique composition and recent evolution. It also provides a gap-free genomic framework for investigating its role in chromosome stability and segregation. In contrast, a study of *M. polymorpha* revealed that its centromeres consist of simple 162-bp satellite repeats and lack extensive pericentromeric heterochromatin and Long Terminal Repeats (LTRs) retrotransposon enrichment typical of flowering plants. Instead, these centromeres are flanked by a specific LINE transposon family [26].

To further advance our understanding of genome evolution and the pivotal role of repetitive elements in the centromere architecture and biology of liverworts, our study provides the first comprehensive, high-quality reference genome assembly of the haploid liverwort *A. endiviifolia* using primary Oxford Nanopore long-read sequencing and Pore-C technology. This high-quality genome assembly and annotation serves as a vital resource for further exploration of liverwort evolution, and provides new perspectives on centromere biology and genome expansion mechanisms in simple thalloid liverworts.

Material and Methods

Sample collection and *in vitro* culture

A. endiviifolia plants were collected from the Nature Reserve of the Sources of the Łyna River (NE Poland; 54.6208°N, 21.2267°E). *In vitro* cultures of *A. endiviifolia* were established in the Plant Biotechnology Laboratory at the Department of Genetics, Plant Breeding and Bioresource Engineering, University of Warmia and Mazury in Olsztyn (Poland). *A. endiviifolia* fragments were washed for 15 min in tap water, surface-disinfected with 0.5% calcium hypochlorite and 0.05% TWEEN-20 for 10 min, and then triple-rinsed with sterile distilled water (5, 10, and 15 min). The sterilization method was highly effective, achieving a culture sterility of 95.65%. Sterile explants were cultured on solid ½ Gamborg's B5 medium (½ basal salts, organics, vitamins [27], 20 g/l sucrose, 8 g/l agar, pH 6.0) at 24°C under 16h light/8h dark. The upper segments of the sterilized plants served as secondary explants for micropropagation on the same medium, spaced 1–2 cm apart. Micropropagated plants were used in the subsequent experiments (Fig. 1).

A voucher specimen for the sequenced sample has been prepared and deposited in the Herbarium of the University of Warmia and Mazury in Olsztyn (OLS), with the accession number OLS-H2024P004.

DNA extraction

For genome assembly, genomic DNA was extracted from the aerial parts of the thallus using a modified ultra-long DNA extraction protocol [28]. Briefly, 500 mg of the material was ground into a fine powder in liquid nitrogen and incubated in 30 mL of homogenization buffer (HB) for 15 min on ice. The suspension was then filtered through a 40 µm cell strainer. Following centrifugation (3,000 × g) and two wash cycles with HB buffer, the pellet was dissolved in SDS lysis buffer containing 5 µL RNase A (20 µg/µL) and 75 µL proteinase K (20 µg/µL) and incubated at 50°C for 3 h. Subsequently, the DNA was extracted using chilled phenol:chloroform:isoamyl alcohol. The DNA was precipitated with isopropanol, washed with 80% ethanol, and eluted in 50 µL of water. The fragment length distribution and DNA integrity were assessed using TapeStation with the Genomic DNA ScreenTape Assay (Agilent), reaching a maximum intensity peak with length > 60,000 bp and DIN 8.2, respectively. The concentration was determined using the Qubit fluorometer HS DNA assay kit and amounted 65.2 ng/µL.

Pore-C procedure

The restriction enzyme Pore-C protocol for plant samples (RE-Pore-C, ONT) was employed to capture three-dimensional DNA interactions within chromatin, with a few modifications as

previously described by Krawczyk et al. [18]. Chromatin was preserved with formaldehyde and the crosslinked plant material was cryogenically ground. The resulting suspension was filtered through a 40 μm strainer, purified, and digested with the NlaIII (NEB) restriction enzyme for 18 hours at 37°C, followed by heat denaturation. Subsequently, a proximity ligation reaction was performed using 40,000 U of T4 DNA ligase for 6 hours at 16°C. This was followed by protein degradation and chromatin de-crosslinking with 100 μl proteinase K (20 μg/μL) and 3 μl RNase A (100 μg/μl) for 18 hours at 56°C, with additional rounds of proteinase K digestion, as per the HiPore-C v1 protocol [29]. DNA was extracted using chilled phenol:chloroform:isoamyl alcohol and EDTA, precipitated with NaCl, washed with ethanol, and eluted with TE buffer. DNA quality was assessed using TapeStation, which reached a peak length of 11,142 bp and DIN 5.8. The concentration was 43 ng/μL, as measured using a Qubit fluorometer.

Nanopore sequencing

Libraries were prepared for nanopore sequencing of native DNA and proximity-ligated DNA fragments using the Ligation Sequencing Kit V14 (SQK-LSK114) following the manufacturer's protocol. DNA sequencing data were generated using the Oxford Nanopore Technologies PromethION 2 platform on R10.4.1 flowcells (PRO-114M) and the MinKNOW sequencing software.

RNA-seq

Total RNA for short read procedure was extracted from four samples of male *A. endiviifolia*: land (i) and water (ii) form, antheridia (iii) and surrounding thallus (iv). This extraction was conducted using the RNA Plant Mini Spin Kit (Qiagen) according to the manufacturer's protocol. Short-read RNA-seq libraries were prepared using two distinct protocols. For RNA extracted from the water and land form of *A. endiviifolia*, libraries were constructed using the TruSeq Stranded Total RNA Library Prep Kit (Illumina) using the Ribo-Zero rRNA option. For antheridia and surrounding thallus, the amount of extracted RNA was insufficient for TruSeq, libraries were prepared using the QIAseq FX Single Cell RNA Library Kit (Qiagen). All libraries were sequenced on an Illumina NovaSeq 6000 platform (Macrogen, Inc., Seoul, South Korea) in 2 × 150 pair-end mode. Raw reads were deposited in BioProject PRJNA1279829 and BioSample SAMN49506713.

Genome size estimation, assembly and quality evaluation

Basecalling

Raw Nanopore signal data from both the standard and Pore-C libraries were basecalled using Dorado v0.9.1 (RRID:SCR_025883) [30]. The superior accuracy model SUP dna_r10.4.1_e8.2_400bps_sup@v5.0.0, was employed to generate high-fidelity basecalls in BAM format. In the case of high molecular weight sequencing reads, the DNA modification v3 models were used to detect all context 6mA, 4mC, and 5mC methylation at the single-base accuracy level.

A fastq file was used to count K-mer frequencies using *kmerfreq* v4.0 [31]. Subsequently, the genome size was estimated using the *GCE* v1.0.2 program [32] and kmerfreq files. The estimated genome size for k-mers from 14 to 21 fell within the 2,950-3,090 Mbp range.

Initial Contig Assembly with Hifiasm

An initial *de novo* assembly was generated directly from the base-called Nanopore long reads using Hifiasm v0.25.0 (RRID:SCR_021069) [33], which supports assembly from high-quality Nanopore data using the–ont option. Simplex basecalled reads were used as input. Hifiasm was run with parameters suitable for Nanopore reads -t64–l0. This produced a set of high-quality initial contigs. To organize the initial contig assembly into chromosome-level structures, we utilized proximity ligation sequencing reads from the Pore-C library processed using the epi2me-labs/wf-pore-c pipeline v1.3.0 [34] to generate a BED file of pairwise chromatin contacts. This contact map served as the input for scaffolding with YaHS v1.2.2 (RRID:SCR_022965) [35] with three iterative rounds. Following automated scaffolding, the resulting assembly and the Pore-C contact map were loaded into Juicerbox v2.17 [36] for manual inspection and curation. Gap-closing of assembly was performed using SAMBA with default options with exception of -m parameter (minimum matching length) set to 9000 as recommended for large plant genomes [37].

The gap-closed assembly was polished using Dorado v0.9.1 aligner and polishing functionalities. The required input for polishing, a sorted BAM file containing alignments of basecalled reads with necessary metadata, was generated using a dorado aligner. This dorado aligner output BAM, along with the gap-closed assembly FASTA, was then processed using the dorado polish command to achieve the final polished assembly.

Quality check

To ensure high quality of the final genome assembly, we employed a multi-faceted approach to evaluate the completeness, structural integrity, and assembly of repetitive regions. Gene

content completeness was assessed using BUSCO v5.8.2 (RRID:SCR_015008) [38] with the viridiplantae_odb10, eukaryota_odb10, and embryophta_odb10 lineage datasets. The structural accuracy and continuity of the nine assembled chromosomes were evaluated using Inspector v1.3.1 [39]. This was performed in a reference-free manner by mapping the long reads back to the assembly to identify potential misassemblies, structural variants, and other inconsistencies. Finally, to assess the completeness of the repetitive landscape, the LTR Assembly Index (LAI) was calculated using the EDTA v2.2.2 (Extensive *de novo* TE Annotator, RRID:SCR_022063) package [40].

Genome annotation

Structural and functional annotation of the genome was conducted using the NCBI EGAPx v0.3.2 pipeline [41] empirical evidence from all four RNA-seq libraries. Based on the provided taxonomy ID, the pipeline automatically selected appropriate protein sets for homology-based evidence, which were aligned to the assembly using miniprot v0.15 [42]. The RNA-seq reads were aligned using STAR v2.7.11 (RRID:SCR_004463) [43] to generate transcript-based evidence. The core of the annotation was performed using Gnomon [44], which first chained the protein and transcript alignments into putative gene models. Gnomon supplemented these with *ab initio* predictions derived from HMM models to identify genes lacking direct evidence. Finally, functional information was added based on the model quality and orthology, and the complete annotation set was generated as a GFF3 file.

Repeatome annotation

To identify and characterize repetitive elements within the *A. endiviifolia* genome, comprehensive repeatome annotation was performed. Initially, repetitive sequences were identified using EDTA v2.2.2 (RRID:SCR_022063) [40]. Further refinement and classification of transposable element (TE) families were performed using TESorter [45], which leverages machine learning to accurately classify diverse TE types. A phylogenetic approach was employed for the specific annotation of LTR retrotransposons. Individual LTR sequences were first aligned using MAFFT (RRID:SCR_011811) [46] with the default settings. Subsequently, based on the obtained alignment and evolutionary relationships among the distinct LTR retrotransposon families, phylogenetic trees were constructed using IQ-TREE 2 (RRID:SCR_017254) with 1000 bootstrap replicates [47].

Identification and characteristics of telomeres and centromeres

The telomeric sequences and centromeric regions within the *A. endiviifolia* genome assembly were identified using quartet (RRID:SCR_025258) [48]. The centromeres of each chromosome were further analyzed and confirmed using CentIER [49]. Both software were used with default settings.

A custom Python script (analyze_ltr_enrichment.py) [50] was developed and employed to identify LTR retrotransposon domain families that are preferentially associated with centromeric regions. Initially, all LTR element sequences were extracted from the whole-genome LTR GFF3 annotation file using reference genome assembly and subjected to six-frame translation. The resulting protein sequences were scanned for conserved LTR protein domains using HMMER (hmmsearch) against the REXdb database. This process generated a comprehensive genome-wide map linking LTR elements (identified by their unique GFF IDs) to their constituent REXdb protein domain types (e.g., Athila, Bryco, and SIRE). Next, these genome-wide LTR domain annotations were used for enrichment analysis within the predefined centromeric regions. For each REXdb domain type, the script calculated the total occupied length and the total count of distinct LTR elements containing that domain separately for centromeric and non-centromeric portions of the genome. The noncentromeric portion was determined by subtracting the total length of the defined centromeric regions from the total genome size. To assess the statistical significance of domain enrichment in centromeres, Fisher's exact test (one-tailed, testing for enrichment) was performed for each domain type based on both its total length and element count in centromeric versus non-centromeric regions.

Synteny analysis

The genomic sequences (FASTA) and gene annotation files (GFF3) for *M. polymorpha* (Tak-1/2 v7.1, GCA_037833965.1) and *A. endiviifolia* (JBRAUX000000000) were obtained from GenBank. The protein sequences for each species were extracted from their respective genomic FASTA and GFF3 files using gffread v0.12.7 (RRID:SCR_018965) [51]. For each gene, the longest protein isoform was retained for downstream analysis.

Gene coordinates were converted into BED6 format using the jcvi.formats.gff bed (v1.5.4 from JCVI utilities [52]) from the GFF3 files. The gene identifiers in these BED files were subsequently cleaned to remove prefixes (e.g., "rna-") and complex locus tag components to ensure compatibility across tools. Specifically, *Marchantia* gene IDs were processed in the format MPTK2_... and *A. endiviifolia* gene IDs were processed in egapxtmp_...-R... by using custom awk scripts. These cleaned BED files provided the chromosome, start, end, cleaned gene ID, dummy score, and strand information. Orthologous gene pairs between *M.*

polymorpha and *A. endiviifolia* were identified using OrthoFinder v3.0.1b1 (RRID:SCR_017118) [53]. The extracted protein sequences from both the species were used as inputs. OrthoFinder was run with DIAMOND v2.1.11 (RRID:SCR_016071) [54] as the sequence search tool for all-vs.-all protein comparisons. Syntenic blocks between *M. polymorpha* and *A. endiviifolia* were identified using the scan action within the jcvi.compara.synteny module (v1.5.4; JCVI utilities [52]). The cleaned BED files and pairwise ortholog files were used as inputs. The analysis was performed using relaxed parameters that are suitable for distantly related species. A minimum of two collinear gene pairs (--min_size=2) was required to define a syntenic block, and a maximum gap of 100 non-collinear genes (--dist=100) was allowed within a block. The output anchor file, containing the identified syntenic gene pairs, was retained. The identified syntenic relationships were visualized as a dot plot using jcvi.graphics.dotplot (v1.5.4, JCVI utilities [52]).

Synteny analysis of *A. endiviifolia* and the second available T2T genome assembly of Jungermanniopsida, *H. hutchinsiae* was performed using ntSynt v1.0.2 [55] since gene annotations file wasn't available in GenBank (GCA_965112325.1).The ntSynt workflow begins by generating ordered minimizer sketches for each genome. These sketches were filtered to retain only the single-copy minimizers present in all assemblies, which were then used to construct an initial graph. After simplifying this graph, linear paths were identified to compute an initial set of synteny blocks. The algorithm refines these by reanalyzing regions not covered by the initial blocks with a smaller window size and augmenting the graph. The pipeline was run with default settings and -d 40 divergence value, and the resulting synteny blocks were then visualized using the ntSynt-viz v1.0.0 pipeline [55]. The *Apopellia* genome was used as the reference, and the strands of chromosomes in the *Herbertus* genome were normalized relative to it. The final visualization of syntenic relationships was rendered as a ribbon plot using gggenomes v1.0.1 [56] R package.

Phylogenomic analysis

Chromosome-scale genome assemblies for 13 liverwort species ranging in size from 200 to 3 Gbp were obtained from the GenBank genome database in FASTA format. Single-copy orthologous genes were identified using BUSCO v5.4.7 (Benchmarking Universal Single-Copy Orthologs, RRID:SCR_015008) [38] with the Embryophyta_odb10 database, which contains 1,614 conserved ortholog groups from 50 plant species. BUSCO was run in genome mode with 32 threads using the default parameters. Custom Python scripts [50] were used to extract the protein sequences of the single-copy orthologs shared across all 14 species. Only

orthologs present in a single copy in all genomes were retained for downstream analysis, resulting in 502 shared single-copy orthologs. Individual protein sequences for each ortholog were aligned using MAFFT v7.505 (RRID:SCR_011811) [46] with the automatic algorithm selection option (--auto), which selects the optimal alignment strategy based on sequence characteristics. The alignments were subsequently trimmed to remove poorly aligned regions using trimAl v1.4 (RRID:SCR_017334) [57] with the automated1 option, which applies a heuristic selection of the optimal automated trimming method. Trimmed alignments of all 502 single-copy orthologs were concatenated into a supermatrix using custom Python scripts. Partition information was recorded for potential partitioned phylogenetic analyses with each gene treated as a separate partition. Maximum likelihood phylogenetic analysis was performed using IQ-TREE v2.2.0 (RRID:SCR_017254) [47] with automatic model selection using ModelFinder [58] implemented in IQ-TREE (-m MFP option). The analysis included 1,000 ultrafast bootstrap replicates [59] to assess branch support (− B 1000). The final phylogenetic tree was visualized using the R package ape v5.6 (RRID:SCR_017343) [60], phangorn v2.10 (RRID:SCR_017302) [61], and phytools (RRID:SCR_015502) [62]. All custom scripts used in this analysis are available at Github repository [50].

Results

Complete reference genome assembly and annotation for *A. endiviifolia*

The integration of 68.5 Gbp of raw data, consisting of 7,751,459 ONT long reads (N50=31 kbp) with a quality score of Q20 of 90.1% and Q30 of 81.3%, along with 103 Gbp of proximity-ligated DNA fragments, producing 434,262,093 reads with a mean quality score of 19, facilitated the generation of a complete assembly of the *A. endiviifolia* reference genome. Raw reads were assembled using hifiasm, resulting in 1860 contigs with a total size of 2,956 Mbp and an N50 length of 76.58 Mbp (Table 1). The initial contigs were then used as the backbone to build scaffold contigs for the chromosomes, incorporating Pore-C data. After gap filling and polishing, the final assembly had a total size of 2,914,960,273 bp with an N50 of 468,157,909 bp (Table 1), comprising nine chromosomes (with six gaps in intergenic regions) ranging from 100,551,284 to 529,742,643 bp in length (Table 2, Fig. 2 and 3).

Assembly accuracy and completeness were evaluated using multiple methodologies. The Pore-C interaction heatmap demonstrated a high degree of consistency across all chromosomes, thereby providing robust evidence of the precision of genome sequencing (Fig. 2). The BUSCO scores for Viridiplantae_odb10 and Eukaryota_odb10 were 99.2% and 95.5%, respectively, with low gene duplication levels of 6.2% and 4.4%, respectively. Furthermore, integrity

assessments of the LTRs indicated an assembled LTR assembly index (LAI) of 20.06. The genome exhibited a consensus quality value (QV) of 47.6 (Table 1). Collectively, these findings underscore the high accuracy and reliability of the *A. endiviifolia* genome assembly. The assembled genome and gene annotation can be found in the NCBI assembly with submission number JBRAUX000000000.

In the process of annotating the genome of *A. endiviifolia*, short-read RNA sequencing was performed on the thallus of both aquatic (16.8 Gbp) and terrestrial forms (20.6 Gbp), as well as on the antheridia themselves (6 Gbp) and surrounding thallus (6.3 Gbp), generating a total of 49.7 Gbp reads. Genomic annotation identified 34,615 genes, including 33,513 protein-coding genes (Table 1). In the comprehensive analysis of the overall distribution of all significant Gene Ontology (GO) terms, the "Biological Process" category contained the highest number of unique terms, significantly surpassing the other categories with 96 terms, accounting for 45.1% of the total. The "Molecular Function" category ranked second in abundance (66 terms, 31%), while the "Cellular Component" category had the fewest unique GO terms (51 terms, 23.9%) (Fig. 4). GO enrichment analysis revealed that six terms predominated among the top 15 significantly enriched GO terms across all ontologies: "poly(A)+ mRNA export from nucleus," "ethylene-activated signaling pathway," "double-stranded DNA binding," "protein-containing complex localization," "protein export from nucleus," and "ribonucleoprotein complex localization" (Fig. 4). The remaining categories contain progressively fewer terms. The composition of the TEs included 12.16% LTRs elements and 57 Helitrons. Among the classified retroelements, the *Copia* and *Gypsy* superfamilies accounted for 8.94% and 2.95% of the assembly, respectively (Fig. 3). The phylogenetic tree illustrates the diversity and evolutionary relationships among LTR retrotransposon families identified in the analyzed genome, including major lineages such as *Athila*, *Phygy*, *Tekay*, and others (Fig. 5). The GC content and gene density were assessed in 500 kbp windows across each chromosome. The average GC content across all chromosomes ranged from 38.8% to 39.6%. The highest gene density was observed on chromosome 6 with a value of 9.78, whereas the lowest gene density was recorded on chromosome 9 with a value of 5.52 (Fig. 3).

Detection and characteristics of telomeres and centromeres

The completion and accuracy of genome sequencing have enabled the identification of telomeres and centromeres (Table 2, Fig. 3). Examination of the telomeric regions by scanning chromosome ends for high-copy tandem repeats showed that both ends of the seven *A. endiviifolia* chromosomes (except chromosomes 3 and 6) feature telomere repeat sequences

(CCCTAAA/TTTAGGG) that align with telomeric structures typical of most plant species. For these seven chromosomes, the number of repeats at the left end ranged from 352 to 481, whereas those at the right end ranged from 402 to 452 (Table 2). In chromosomes 3 and 6, the typical telomeric sequence was identifiable at one end, whereas the opposite end exhibited a substantial number of repeats of an alternative motif. Specifically, the left end of chromosome 3 contained 115 repeats of the ACGCAGC motif, whereas the right end of chromosome 6 contained 176 repeats of the TGCGTCG motif (Table 2).

The application of quarTeT, a tool that identifies centromeres by computationally detecting and mapping tandem repeats and associated retrotransposons in assembled genomes, in conjunction with CentIER, which identifies centromeres by clustering tandem repeats (Supplementary Table S1), mapping their abundance and distribution, and designating regions with extensive dense arrays as candidate centromeres, facilitated reliable identification of centromeric regions across all nine chromosomes. However, to confirm the presence of centromeres in chromosomes 4 and 5, it was necessary to verify the results obtained from the quarTeT analysis by comparing them with the gaps observed in the Pore-C interaction heatmap (Supplementary Fig. S1). Furthermore, all sites identified as centromeres on the remaining seven chromosomes were corroborated by the Pore-C interaction heatmap. Examination of these centromeric regions confirmed that LTR/*Gypsy* elements were highly enriched in the immediate vicinity of the centromere and their abundance decreased as the distance from the centromere increased, as presented in detail for chromosome 1 (Fig. 6). Additionally, this analysis demonstrated a correlation between high LTR/*Gypsy* density and formation of a specialized chromatin domain at the centromere. Moreover, LTR domains from the *Ty3/Gypsy* superfamily, particularly those belonging to the *Tat* (*TatI*, *TatII*, and *TatIII*), *Phygy*, and *Selgy* family, were found to be significantly enriched in centromeric regions compared to non-centromeric chromosomal regions. Although LTR elements from the *Bel-Pao* family are infrequently present in centromeres, they nonetheless showed a statistically significant enrichment in these regions relative to the rest of the genome (Fig. 7). Finally, the validated centromeres exhibited length variation ranging from 1.9 to 5 Mbp (Table 2). Compared to *A. endiviifolia*, *Marchantia polymorpha* centromeres are similarly structured with abundant retroelements and are typically centrally located, ranging from about 176 to 604 Kbp [63]. *Physcomitrium patens* centromeres share this organization but tend to be smaller, varying from 64 to 221 Kbp, likely reflecting its more compact genome [25]. These differences highlight centromere size and composition diversity within bryophytes and demonstrate the value of long-read sequencing for resolving complex genomic regions.

Genome synteny analysis

Collinearity between the nine chromosomes of *A. endiviifolia* and the leafy liverwort *Herbertus hutchinsiae*, both belonging to the Jungermanniopsida class, revealed complex and divergent synteny patterns characterized by fragmentation into small syntenic blocks distributed across multiple chromosomes rather than forming extensive chromosome-scale fusions. A total of 429 syntenic blocks spanning approximately 4.8 Mb were detected, with a median size of about 5.2 kb and a mean size of 11.2 kb, indicating small, fragmented conserved regions. These blocks were evenly distributed in terms of strand orientation, with 201 blocks on the positive strand and 228 on the negative strand. Details of the detected syntenic blocks between *A. endiviifolia* and *H. hutchinsiae* chromosomes, including chromosome coordinates and strand orientation, are provided in Supplementary Table S2 and S3. Structural variation analysis revealed numerous chromosomal breakpoints across chromosomes, predominantly in chromosome 1 (54 translocation and 18 inversion breakpoints), while chromosome 9 showed no breakpoints, consistent with its slightly more conserved relationship (Fig. 8, Supplementary Table S3). Furthermore, analysis of genomic conservation and structural variations between *A. endiviifolia* and the model liverwort *M. polymorpha* did not reveal significant collinearity (Supplementary Fig. S2).

Phylogenetic relationship analysis

The protein sequences of single-copy orthologs conserved across *A. endiviifolia* and 13 other liverwort species with chromosome-scale genome assemblies were selected for phylogenetic analysis to elucidate evolutionary relationships among these species. The resulting phylogenetic tree clearly separated Marchantiopsida and Jungermanniopsida into distinct evolutionary lineages. Notably, all internal nodes in the tree were supported by 100% bootstrap values, reflecting the maximal confidence in the overall tree topology. This strong support underscores the reliability of the inferred evolutionary relationships. Additionally, the observed variation in genome size, particularly in the large genomes of some Jungermanniopsida, may reflect lineage-specific differences in genome dynamics, such as variation in transposable element activity, gene duplication, and structural rearrangements (Fig. 9).

Re-use potential

The telomere-to-telomere reference genome assembly of the haploid liverwort *A. endiviifolia* is a high-quality genomic resource with a broad potential for reuse across multiple research

fields. Generated using Oxford Nanopore long-read sequencing combined with Pore-C technology, this 2,914,960,273 bp assembly achieves chromosome-scale resolution with exceptional completeness (99.2% BUSCO) and accuracy (QV 47.6). It comprises nine experimentally validated chromosomes featuring 18 telomeres and nine predicted centromeres enriched in LTR/*Gypsy* retrotransposons, and includes 34,615 annotated genes. The unusual presence of non-canonical telomeric-like repeats (ACGCAGC and TGCGTCG) on chromosomes 3 and 6 in *A. endiviifolia* suggests species-specific telomere evolution or chromosomal rearrangements, though their biological significance is unclear. Further comparative and functional studies are needed to understand their origin and role. These findings contribute to the genomic landscape of *A. endiviifolia* and support future research on plant telomere diversity and evolution. Notably, the centromeres have been predicted solely through in silico analyses, representing a plausible but not yet fully validated hypothesis.

This comprehensive dataset enables comparative genomics within liverworts and across land plants, facilitating studies of genome evolution, centromere biology, chromosome end structures, genome stability, and chromosome segregation. By providing a complete, well-annotated, and experimentally validated genome, this resource provides a robust foundation for future research in plant genomics, cytogenetics, and evolutionary biology, extending beyond the scope of the current study.

Data Availability

The genomic and transcriptomic sequence data is deposited in NCBI BioProject PRJNA1279829. All additional supporting data are available in the *GigaScience* repository, GigaDB [64].

Contributions

J.S.-P. and J.S. contributed to research design, J.S-P. carried out laboratory analyses, J.S. carried out bioinformatic analyses, visualized data and prepared figures, P.S. was responsible for in vitro cultures, J.S.-P. and J.S. analyzed the data and wrote the original draft. J.S. obtained funding. All authors revised and approved the final version of the manuscript.

Funding

This study was financially supported by the National Science Center, Kraków, Poland (Grant No. 2020/39/B/NZ8/02504.

Acknowledgements

We would like to thank Dr. Katarzyna Krawczyk for her assistance with the Pore-C procedure. We also extend our gratitude to the staff of the Department of Botany and Evolutionary Ecology at the University of Warmia and Mazury in Olsztyn for the pleasant working atmosphere.

References

1. Bechteler J, Peñaloza-Bojacá G, Bell D, Gordon Burleigh J, McDaniel SF, Christine Davis E, et al.. Comprehensive phylogenomic time tree of bryophytes reveals deep relationships and uncovers gene incongruences in the last 500 million years of diversification. *Am J Bot*. John Wiley and Sons Inc; 2023; doi: 10.1002/AJB2.16249.

2. Donoghue PCJ, Harrison CJ, Paps J, Schneider H. The evolutionary emergence of land plants. *Current Biology*. Cell Press; 2021; doi: 10.1016/J.CUB.2021.07.038.

3. Harris BJ, Clark JW, Schrempf D, Szöllősi GJ, Donoghue PCJ, Hetherington AM, et al.. Divergent evolutionary trajectories of bryophytes and tracheophytes from a complex common ancestor of land plants. *Nat Ecol Evol*. Nature Research; 2022; doi: 10.1038/S41559-022-01885-X;TECHMETA=42,45;SUBJMETA=181,2669,449,631,757;KWRD=PHYLOGENETICS,PLANT+EVOLUTION.

4. Linde AM, Singh S, Bowman JL, Eklund M, Cronberg N, Lagercrantz U. Genome Evolution in Plants: Complex Thalloid Liverworts (Marchantiopsida). *Genome Biol Evol*. Oxford University Press; 2023; doi: 10.1093/GBE/EVAD014.

5. Villarreal A. JC, Crandall-Stotler BJ, Hart ML, Long DG, Forrest LL. Divergence times and the evolution of morphological complexity in an early land plant lineage (Marchantiopsida) with a slow molecular rate. *New Phytologist*. Blackwell Publishing Ltd; 2016; doi: 10.1111/NPH.13716,.

6. Renner MAM, Heslewood MM, Patzak SDF, Schäfer-Verwimp A, Heinrichs J. By how much do we underestimate species diversity of liverworts using morphological evidence? An example from Australasian Plagiochila (Plagiochilaceae: Jungermanniopsida). *Mol Phylogenet Evol*. Academic Press; 2017; doi: 10.1016/J.YMPEV.2016.12.018.

7. Söderström L, Hagborg A, Von Konrat M, Bartholomew-Began S, Bell D, Briscoe L, et al.. World checklist of hornworts and liverworts. *PhytoKeys 59: 1-828*. Pensoft Publishers; 2016; doi: 10.3897/PHYTOKEYS.59.6261.

8. Paukszto Ł, Górski P, Krawczyk K, Maździarz M, Szczecińska M, Ślipiko M, et al.. The organellar genomes of Pellidae (Marchantiophyta): the evidence of cryptic speciation, conflicting phylogenies and extraordinary reduction of mitogenomes in simple thalloid liverwort lineage. *Sci Rep*. Nature Research; 2023; doi: 10.1038/S41598-023-35269-3,.

9. Crandall-Stotler B, Stotler RE, Zhang L, Forrest LL. On the morphology, systematics and phylogeny of Noteroclada (Noterocladaceae, Marchantiophyta). *Nova Hedwigia*. Schweizerbart'sche Verlagsbuchhandlung; 2010; doi: 10.1127/0029-5035/2010/0091-0421.

10. Schütz N, Quandt D, Nebel M. The position of the genus Apopellia stat. Nov. within the Pelliales (Marchantiophytina: Jungermanniopsida). *Taxon*. International Association for Plant Taxonomy; 2016; doi: 10.12705/652.1.

11. Crandall-Stotler B, Stotler RE, Long DG. PHYLOGENY AND CLASSIFICATION OF THE MARCHANTIOPHYTA. *Edinb J Bot*. Cambridge University Press; 2009; doi: 10.1017/S0960428609005393.

12. Sawicki J, Krawczyk K, Ślipiko M, Szandar K, Szczecińska M. Comparative analysis of apopellia endiviifolia plastomes reveals a strikingly high level of differentiation between its terrestrial and water form. *Diversity (Basel)*. MDPI; 2021; doi: 10.3390/D13120674/S1.
13. : Interpretation of Electrophoretic Patterns in Population Genetics of Bryophytes: VI. Genetic Variation and Evolution of the Liverwort Genus Pellia with Special Reference to Central European Territory on JSTOR. https://www.jstor.org/stable/20149587?seq=1 Accessed 2025 Jun 25.
14. Pellicer J, Hidalgo O, Dodsworth S, Leitch IJ. Genome Size Diversity and Its Impact on the Evolution of Land Plants. *Genes (Basel)*. MDPI AG; 2018; doi: 10.3390/GENES9020088.
15. Temsch EM, Greilhuber J, Krisai R. Genome size in liverworts. *Preslia*. 82:63–802010;
16. Bainard JD, Forrest LL, Goffinet B, Newmaster SG. Nuclear DNA content variation and evolution in liverworts. *Mol Phylogenet Evol*. Academic Press; 2013; doi: 10.1016/J.YMPEV.2013.04.008.
17. Singh S, Bowman JL. The monoicous secondarily aquatic liverwort Ricciocarpos natans as a model within the radiation of derived Marchantiopsida. *Front Plant Sci*. Frontiers Media SA; 2023; doi: 10.3389/FPLS.2023.1260596/ENDNOTE.
18. Krawczyk K, Szablińska-Piernik J, Paukszto Ł, Maździarz M, Sulima P, Przyborowski JA, et al.. Chromosome-scale telomere to telomere genome assembly of common crystalwort (Riccia sorocarpa Bisch.). *Scientific Data 2025 12:1*. Nature Publishing Group; 2025; doi: 10.1038/s41597-025-04373-6.
19. Levins J, Paukszto Ł, Krawczyk K, Maździarz M, Arch BC, Cargill DC, et al.. Evolution of sexual systems and regressive evolution in Riccia. *New Phytologist*. John Wiley & Sons, Ltd; 2025; doi: 10.1111/NPH.20454.
20. Fu Y, Zhang X, Zhang T, Sun W, Yang W, Shi Y, et al.. Evidence for evolution of a new sex chromosome within the haploid-dominant Marchantiales plant lineage. *J Integr Plant Biol*. John Wiley and Sons Inc; 2025; doi: 10.1111/JIPB.13867/SUPPINFO.
21. Bowman JL, Kohchi T, Yamato KT, Jenkins J, Shu S, Ishizaki K, et al.. Insights into Land Plant Evolution Garnered from the Marchantia polymorpha Genome. *Cell*. Cell Press; 2017; doi: 10.1016/J.CELL.2017.09.030.
22. Linde AM, Eklund DM, Cronberg N, Bowman JL, Lagercrantz U. Rates and patterns of molecular evolution in bryophyte genomes, with focus on complex thalloid liverworts, Marchantiopsida. *Mol Phylogenet Evol*. Academic Press; 2021; doi: 10.1016/J.YMPEV.2021.107295.
23. Miga KH. Centromere studies in the era of 'telomere-to-telomere' genomics. *Exp Cell Res*. Elsevier Inc.; 2020; doi: 10.1016/J.YEXCR.2020.112127.
24. Peng D, Hong Z, Kan S, Wu Z, Liao X. The telomere-to-telomere (T2T) genome provides insights into the evolution of specialized centromere sequences in sandalwood. *Gigascience*. 2024; doi: 10.1093/gigascience/giae096.
25. Bi G, Zhao S, Yao J, Wang H, Zhao M, Sun Y, et al.. Near telomere-to-telomere genome of the model plant Physcomitrium patens. *Nature Plants* . Nature Research; 2024; doi: 10.1038/s41477-023-01614-7.
26. Montgomery SA, Tanizawa Y, Galik B, Wang N, Ito T, Mochizuki T, et al.. Chromatin Organization in Early Land Plants Reveals an Ancestral Association between H3K27me3, Transposons, and Constitutive Heterochromatin. *Curr Biol*. Cell Press; 2020; doi: 10.1016/J.CUB.2019.12.015.
27. Sawicki J, Krawczyk K, Kurzyński M, Maździarz M, Paukszto Ł, Sulima P, et al.. Nanopore sequencing of organellar genomes revealed heteroplasmy in simple thalloid and

leafy liverworts. *Acta Societatis Botanicorum Poloniae*. Polish Botanical Society; 2023; doi: 10.5586/ASBP/172516.

28. Lu D, Liu C, Ji W, Xia R, Li S, Liu Y, et al.. Nanopore ultra-long sequencing and adaptive sampling spur plant complete telomere-to-telomere genome assembly. *Mol Plant*. Cell Press; 2024; doi: 10.1016/J.MOLP.2024.10.008.

29. Zhong JY, Niu L, Lin Z Bin, Bai X, Chen Y, Luo F, et al.. High-throughput Pore-C reveals the single-allele topology and cell type-specificity of 3D genome folding. *Nature Communications 2023 14:1*. Nature Publishing Group; 2023; doi: 10.1038/s41467-023-36899-x.

30. : GitHub - nanoporetech/dorado: Oxford Nanopore's Basecaller. https://github.com/nanoporetech/dorado Accessed 2025 Sep 10.

31. Liu B, Shi Y, Yuan J, Hu X, Zhang H, Li N, et al.. Estimation of genomic characteristics by analyzing k-mer frequency in de novo genome projects. 2013;

32. Wang H, Liu B, Zhang Y, Jiang F, Ren Y, Yin L, et al.. Estimation of genome size using k-mer frequencies from corrected long reads. 2020;

33. Cheng H, Concepcion GT, Feng X, Zhang H, Li H. Haplotype-resolved de novo assembly using phased assembly graphs with hifiasm. *Nat Methods*. Nature Research; 2021; doi: 10.1038/S41592-020-01056-5;SUBJMETA=114,208,212,2302,2785,61,631,794;KWRD=GENOME+ASSEMBLY+ALGORITHMS,SOFTWARE.

34. : GitHub - epi2me-labs/wf-pore-c. https://github.com/epi2me-labs/wf-pore-c Accessed 2025 Sep 10.

35. Zhou C, McCarthy SA, Durbin R. YaHS: yet another Hi-C scaffolding tool. *Bioinformatics*. Oxford Academic; 2023; doi: 10.1093/BIOINFORMATICS/BTAC808.

36. Durand NC, Robinson JT, Shamim MS, Machol I, Mesirov JP, Lander ES, et al.. Juicebox Provides a Visualization System for Hi-C Contact Maps with Unlimited Zoom. *Cell Syst*. Cell Press; 2016; doi: 10.1016/j.cels.2015.07.012.

37. : GitHub - alekseyzimin/masurca. https://github.com/alekseyzimin/masurca Accessed 2025 Sep 10.

38. Manni M, Berkeley MR, Seppey M, Simão FA, Zdobnov EM. BUSCO Update: Novel and Streamlined Workflows along with Broader and Deeper Phylogenetic Coverage for Scoring of Eukaryotic, Prokaryotic, and Viral Genomes. *Mol Biol Evol*. Oxford University Press; 2021; doi: 10.1093/MOLBEV/MSAB199,.

39. Chen Y, Zhang Y, Wang AY, Gao M, Chong Z. Accurate long-read de novo assembly evaluation with Inspector. *Genome Biol*. BioMed Central Ltd; 2021; doi: 10.1186/S13059-021-02527-4/FIGURES/4.

40. Ou S, Su W, Liao Y, Chougule K, Agda JRA, Hellinga AJ, et al.. Benchmarking transposable element annotation methods for creation of a streamlined, comprehensive pipeline. *Genome Biol*. BioMed Central Ltd.; 2019; doi: 10.1186/S13059-019-1905-Y/FIGURES/6.

41. : GitHub - ncbi/egapx: Eukaryotic Genome Annotation Pipeline-External caller scripts and documentation. https://github.com/ncbi/egapx Accessed 2025 Sep 10.

42. Li H. Protein-to-genome alignment with miniprot. *Bioinformatics*. Oxford University Press; 2023; doi: 10.1093/BIOINFORMATICS/BTAD014,.

43. Dobin A, Davis CA, Schlesinger F, Drenkow J, Zaleski C, Jha S, et al.. STAR: Ultrafast universal RNA-seq aligner. *Bioinformatics*. Bioinformatics; 2013; doi: 10.1093/BIOINFORMATICS/BTS635,.

44. : Gnomon - the NCBI eukaryotic gene prediction tool. https://www.ncbi.nlm.nih.gov/refseq/annotation_euk/gnomon/ Accessed 2025 Sep 10.

45. Zhang RG, Li GY, Wang XL, Dainat J, Wang ZX, Ou S, et al.. TEsorter: An accurate and fast method to classify LTR-retrotransposons in plant genomes. *Hortic Res*. Oxford University Press; 2022; doi: 10.1093/HR/UHAC017.

46. Katoh K, Standley DM. MAFFT Multiple Sequence Alignment Software Version 7: Improvements in Performance and Usability. *Mol Biol Evol*. Oxford Academic; 2013; doi: 10.1093/MOLBEV/MST010.

47. Minh BQ, Schmidt HA, Chernomor O, Schrempf D, Woodhams MD, Von Haeseler A, et al.. IQ-TREE 2: New Models and Efficient Methods for Phylogenetic Inference in the Genomic Era. *Mol Biol Evol*. Oxford University Press; 2020; doi: 10.1093/MOLBEV/MSAA015,.

48. Lin Y, Ye C, Li X, Chen Q, Wu Y, Zhang F, et al.. quarTeT: a telomere-to-telomere toolkit for gap-free genome assembly and centromeric repeat identification. *Hortic Res*. Oxford Academic; 2023; doi: 10.1093/HR/UHAD127.

49. Xu D, Yang J, Wen H, Feng W, Zhang X, Hui X, et al.. CentIER: Accurate centromere identification for plant genomes. *Plant Commun*. Elsevier; 2024; doi: 10.1016/J.XPLC.2024.101046.

50. : GitHub - kubek78/PhylogenomicsME: Phylogenomics Made Easy. https://github.com/kubek78/PhylogenomicsME/tree/main Accessed 2025 Sep 10.

51. Pertea G, Pertea M. GFF Utilities: GffRead and GffCompare. *F1000Res*. NLM (Medline); 2020; doi: 10.12688/F1000RESEARCH.23297.2/DOI.

52. Tang H, Krishnakumar V, Zeng X, Xu Z, Taranto A, Lomas JS, et al.. JCVI: A versatile toolkit for comparative genomics analysis. *iMeta*. John Wiley & Sons, Ltd; 2024; doi: 10.1002/IMT2.211.

53. Emms DM, Kelly S. OrthoFinder: Phylogenetic orthology inference for comparative genomics. *Genome Biol*. BioMed Central Ltd.; 2019; doi: 10.1186/S13059-019-1832-Y,.

54. Buchfink B, Reuter K, Drost HG. Sensitive protein alignments at tree-of-life scale using DIAMOND. *Nat Methods*. Nature Research; 2021; doi: 10.1038/S41592-021-01101-X,.

55. Coombe L, Kazemi P, Wong J, Birol I, Warren RL. Multi-genome synteny detection using minimizer graph mappings. *bioRxiv*. Cold Spring Harbor Laboratory; 2024; doi: 10.1101/2024.02.07.579356.

56. Hackl T, Ankenbrand M, van Adrichem B, Wilkins D, Haslinger K. gggenomes: effective and versatile visualizations for comparative genomics. 2024;

57. Capella-Gutiérrez S, Silla-Martínez JM, Gabaldón T. trimAl: a tool for automated alignment trimming in large-scale phylogenetic analyses. *Bioinformatics*. Oxford Academic; 2009; doi: 10.1093/BIOINFORMATICS/BTP348.

58. Kalyaanamoorthy S, Minh BQ, Wong TKF, Von Haeseler A, Jermiin LS. ModelFinder: Fast model selection for accurate phylogenetic estimates. *Nat Methods*. Nature Publishing Group; 2017; doi: 10.1038/NMETH.4285;SUBJMETA=114,181,631,739;KWRD=COMPUTATIONAL+BIOLOGY+AND+BIOINFORMATICS,EVOLUTION,PHYLOGENY.

59. Hoang DT, Chernomor O, Von Haeseler A, Minh BQ, Vinh LS. UFBoot2: Improving the Ultrafast Bootstrap Approximation. *Mol Biol Evol*. Oxford Academic; 2018; doi: 10.1093/MOLBEV/MSX281.

60. Paradis E, Schliep K. ape 5.0: an environment for modern phylogenetics and evolutionary analyses in R. *Bioinformatics*. Oxford Academic; 2019; doi: 10.1093/BIOINFORMATICS/BTY633.

61. Schliep KP. phangorn: phylogenetic analysis in R. *Bioinformatics*. Oxford Academic; 2011; doi: 10.1093/BIOINFORMATICS/BTQ706.
62. Revell LJ. phytools 2.0: an updated R ecosystem for phylogenetic comparative methods (and other things). *PeerJ*. PeerJ Inc.; 2024; doi: 10.7717/PEERJ.16505,.
63. Tanizawa Y, Mochizuki T, Yagura M, Sakamoto M, Fujisawa T, Kawamura S, et al.. MarpolBase: Genome database for Marchantia polymorpha featuring high quality reference genome sequences. *bioRxiv*. Cold Spring Harbor Laboratory; 2025; doi: 10.1101/2025.03.30.646155.
64. Szablinska-Piernik J, Sulima P, Sawicki J. Supporting data for "Giant chromosomes of a tiny plant - the complete telomere-to-telomere genome assembly of the simple thalloid liverwort Apopellia endiviifolia (Jungermanniopsida, Marchantiophyta)" GigaScience Database. 2025. https://doi.org/10.5524/102776

Table 1. Assembly statistics

**Genome assembly statistics**	
Genome size [bp]	2,914,960,273
Genome coverage [median]	47.6
Number of chromosomes	9
Number of telomeres	18
Number of centromeres	9
Contig N50 [bp]	76,579,043
Scaffold N50 [bp]	468,157,909
Number of gaps	6
Number of total/protein-coding genes	34,615/33,513
QV	47.6
LAI	20.06
BUSCO - genome (Eukaryota/Viridiplantae/Embryophyta) [%]	C:99.2/95.5/63.2 [S:93.0/91.1/60.0, D:6.2/4.4/3.2]

Table 2. The identified telomeres and centromeres in *A. endiviifolia* assembly

Chromosomes	Length [bp]	Telomeres		Centromeres	
		Number of repeats at the left end	Number of repeats at the right end	Start	End
Ch1	529,742,643	425	418	359,400,001	363,400,000
Ch2	473,504,363	352	422	232,800,001	237,800,000
Ch3	468,157,909	115*	414	446,400,001	449,400,000
Ch4	317,755,521	408	402	239,340,762**	241,950,761**
Ch5	310,129,949	481	421	37,548,478**	39,527,839**
Ch6	244,886,949	419	176*	242,160,000	244,159,999
Ch7	236,110,306	393	452	189,670,000	191,669,999
Ch8	234,121,349	428	403	202,730,000	204,729,999
Ch9	100,551,284	446	420	89,933,334	93,766,667

* - telomeres with a non-canonical ACGCAGC/TGCGTCG motif

** - centromeres verification based only on quarTeT and Pore-C contact map

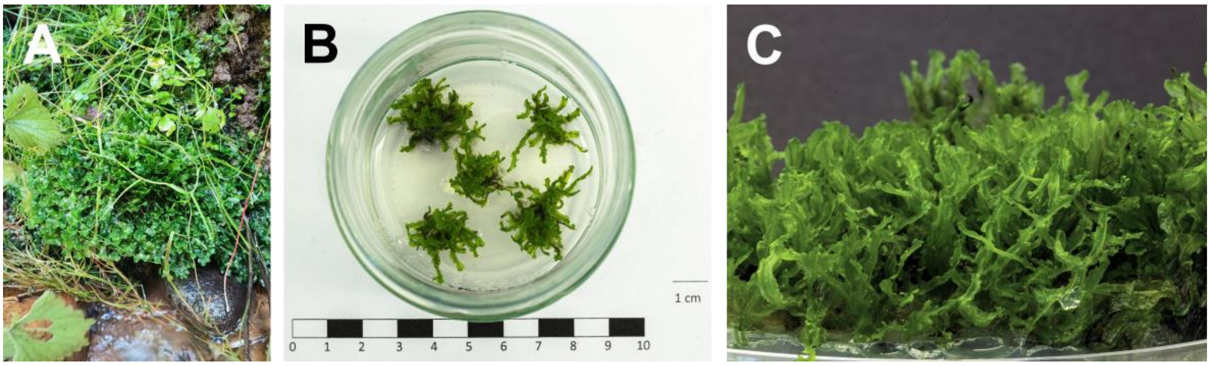

Figure 1. *A. endiviifolia* plants located in the Nature Reserve of the Sources of the Łyna River (A), *in vitro* culture (B), and thalli morphology under *in vitro* conditions (C).

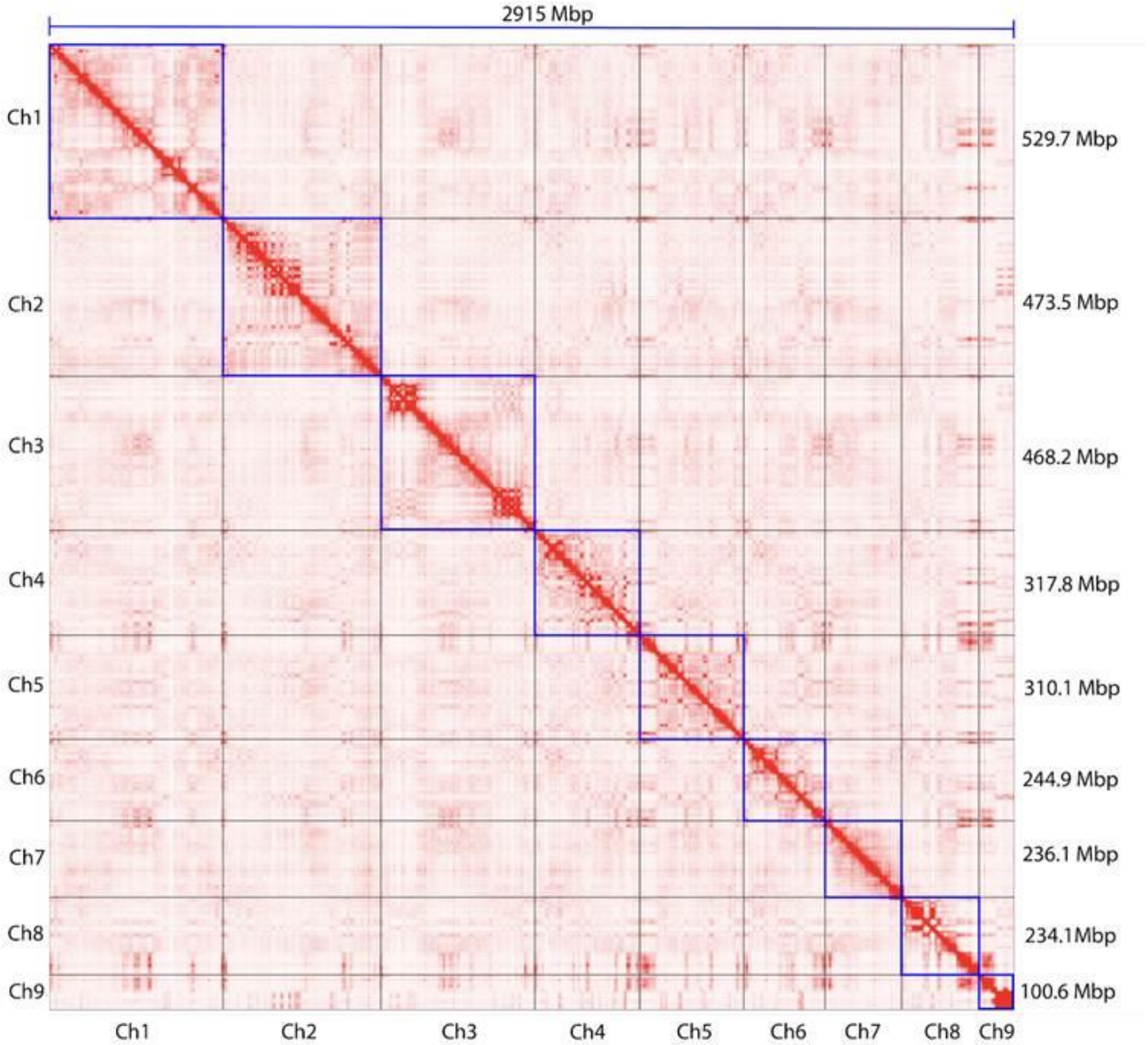

Figure 2. Pore-C interaction heatmap of the *A. endiviifolia* genome.

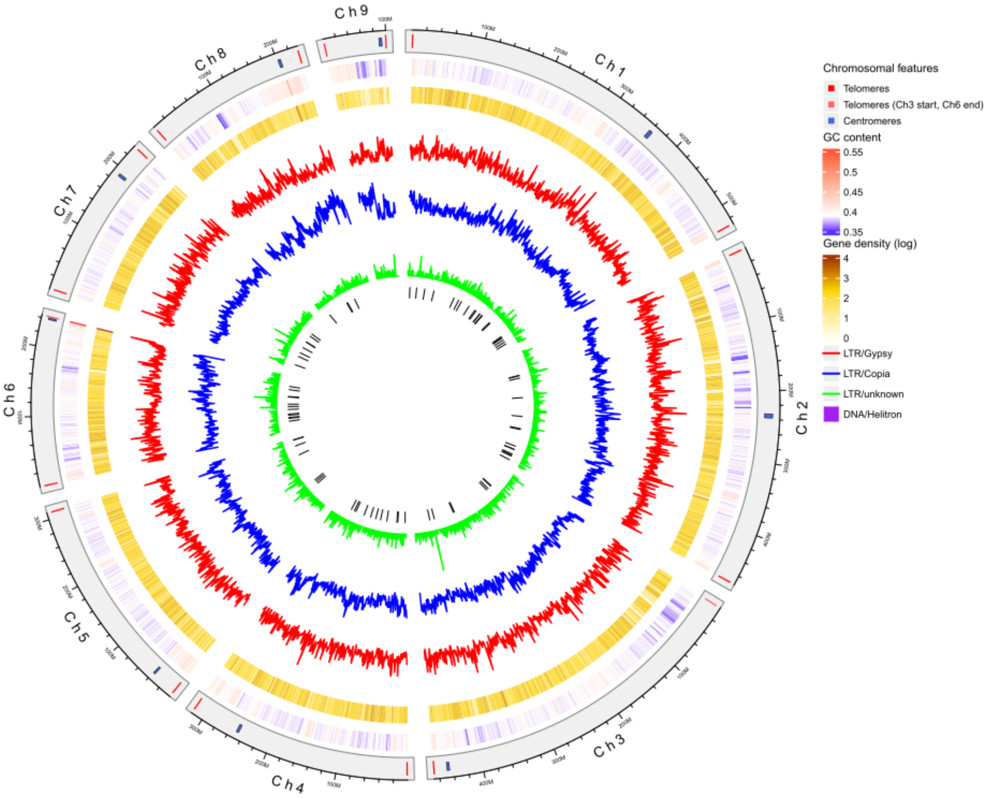

Figure 3. Circos plot showing detailed characterization of the nine *A. endiviifolia* chromosomes. From outside to inside: length of chromosomes in Mbp with marked telomeric (red bars) and centromeric regions (blue bars), GC content, gene density, distribution of LTR/*Gypsy* transposons, distribution of LTR/*Copia* transposons, distribution of LTR/unknown transposons, DNA/Helitron locations.

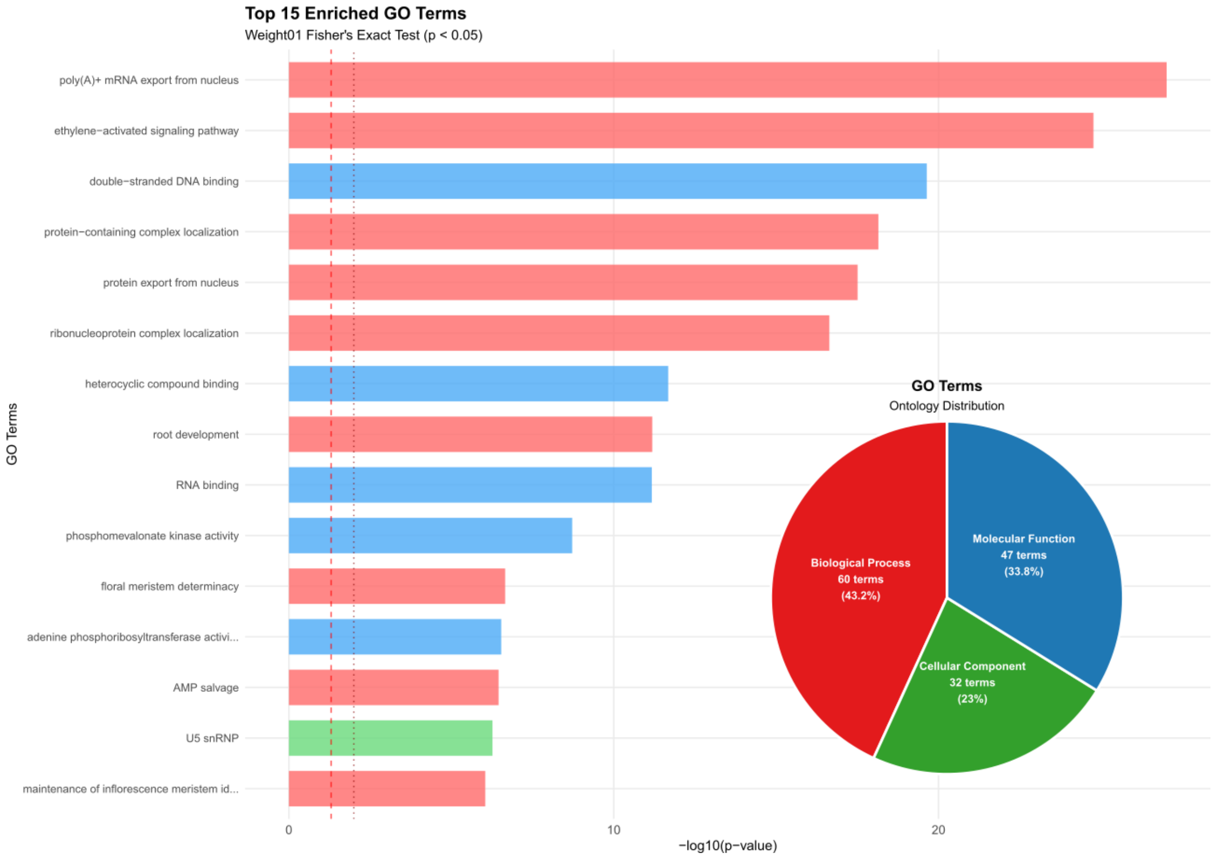

Figure 4. Distribution of all significant GO terms in the genome and the top 15 enriched GO terms across all ontologies in the genome.

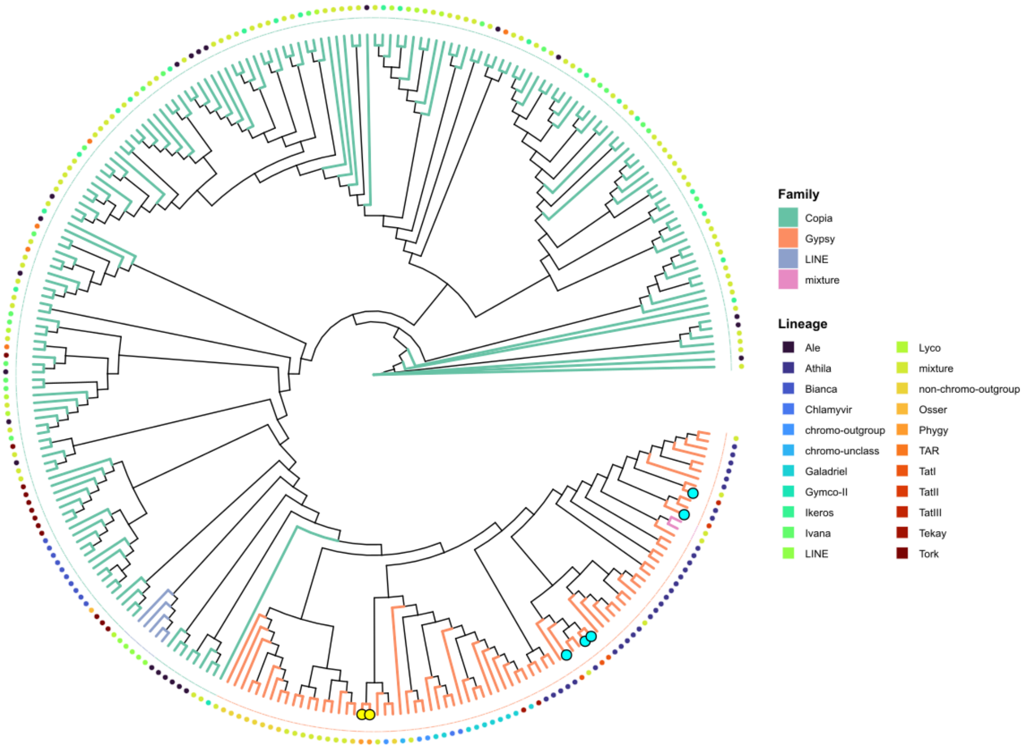

Figure 5. Maximum likelihood phylogenetic tree of LTR retrotransposon lineages identified in the analyzed genome. The tips highlighted in color represent LTR elements enriched in the centromeric regions.

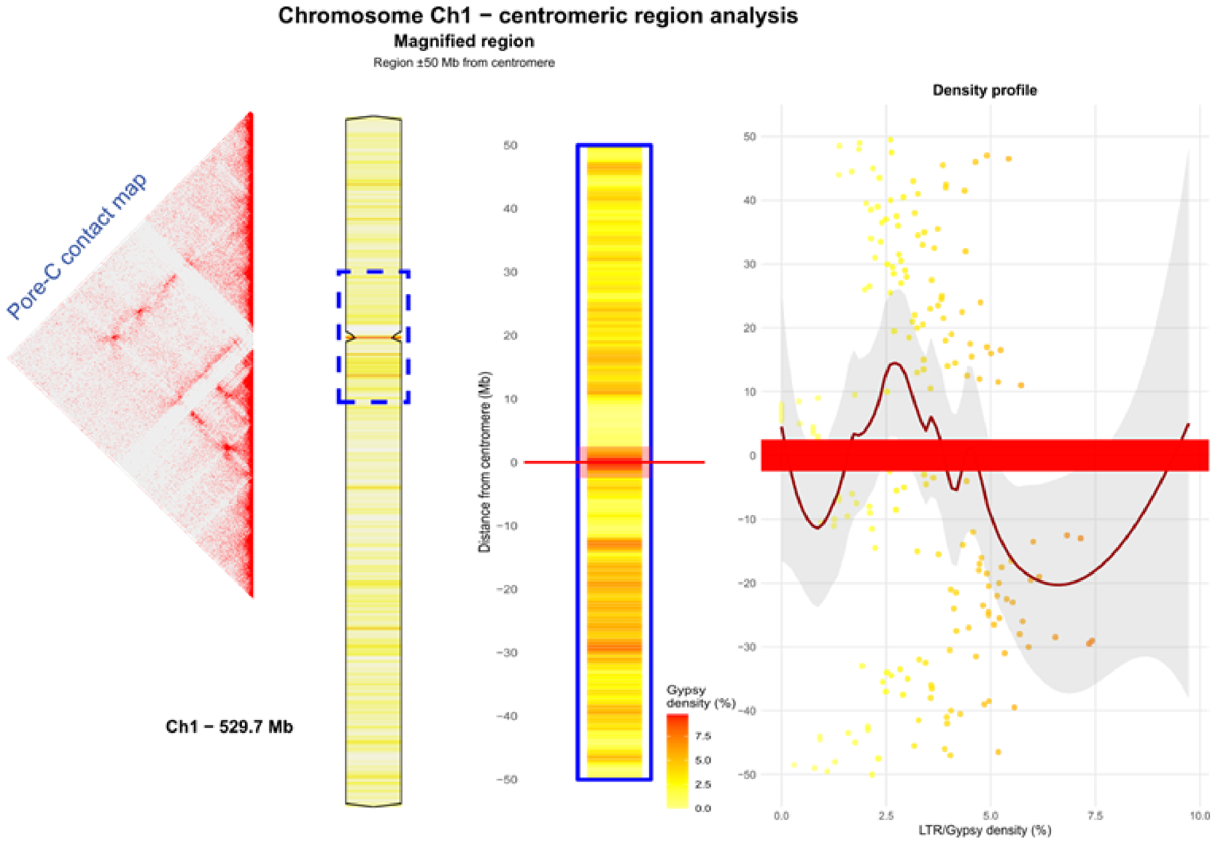

Figure 6. Detailed examination of the example centromere – Analysis of *Gypsy* retrotransposon distribution and chromatin interactions across chromosome 1. In the density profile, each dot represents a single genomic window, plotting its LTR/Gypsy density (X-axis) against its chromosomal position (Y-axis). The solid dark red line shows the overall trend in density, calculated using a LOESS regression, while the surrounding light grey shaded area indicates the 95% confidence interval for this trend. The thick red horizontal bar marks the position of the centromere across the center of the magnified plots.

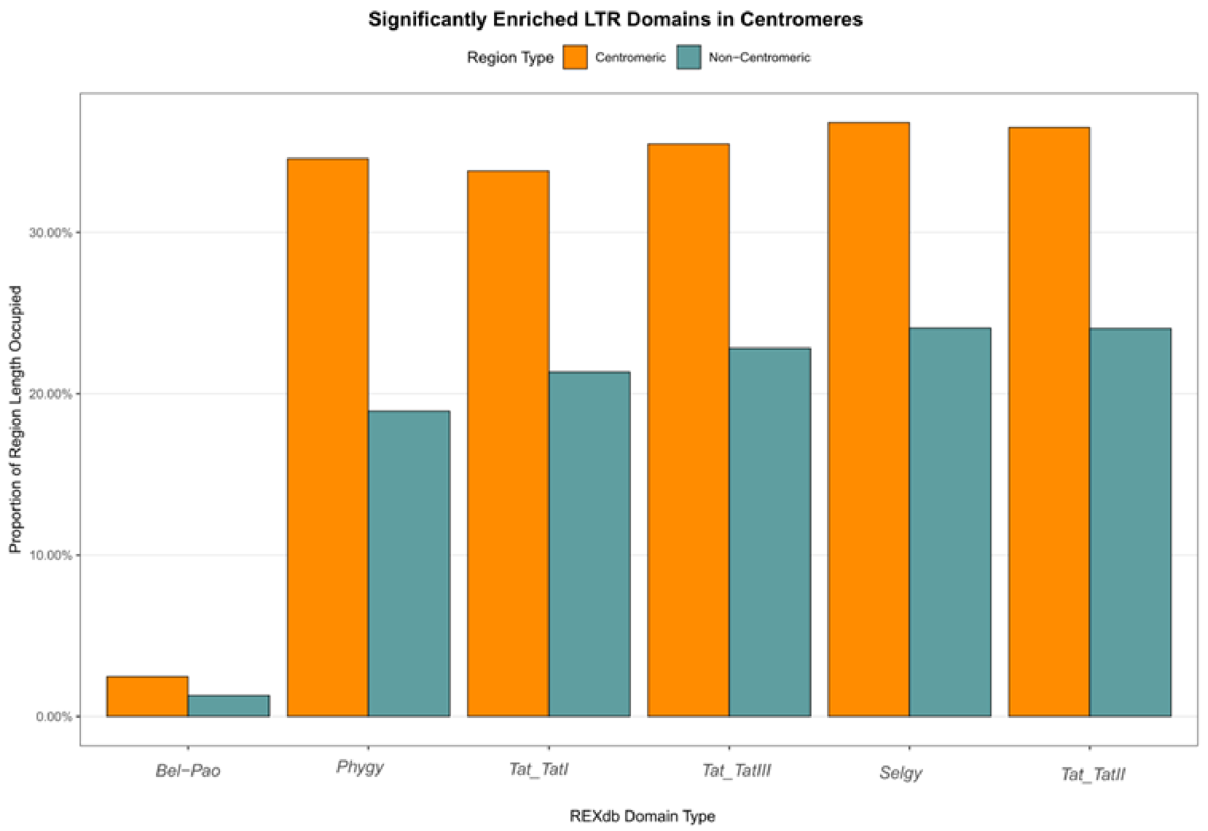

Figure 7. The proportion of regional length occupied by the top six significantly ($p<0.05$) enriched LTR domains in centromeric regions compared with non-centromeric regions.

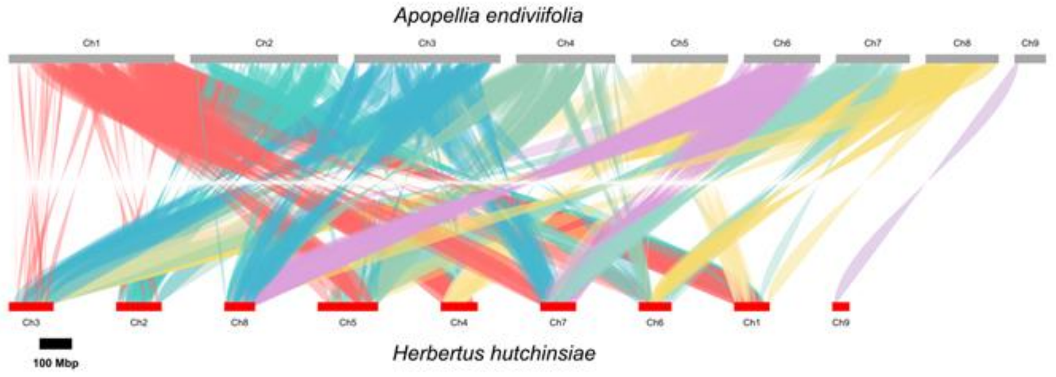

Figure 8. Synteny plot of the differences and similarities between the assembly of *A. endiviifolia* chromosomes and that of *H. hutchinsiae*. Colored lines connect collinear regions between the species' genomes.

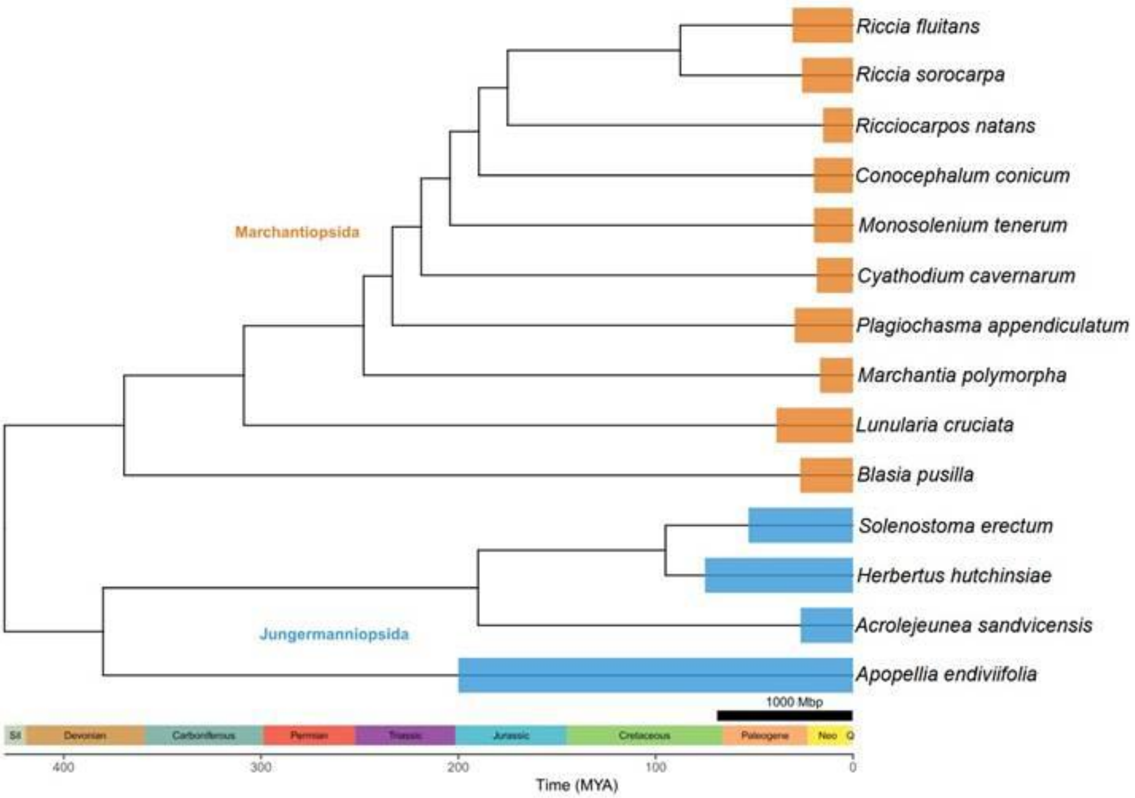

Figure 9. Phylogenetic relationships among liverworts determined by conserved single-copy orthologs. Size of orange and blue boxes is proportional to the genome size of the species. All nodes have the maximum statistical support.

Supplementary Table S1. Tandem centromeric repeats of *in silico* validated centromeric regions in *A. endiviifolia* assembly. Centromeres in chromosomes 1-3 and 6-9 were identified by the Centier program, and centromeres in chromosomes 4 and 5 were identified by the quarTeT program. All centromeres were validated using a Pore-C interaction heatmap.

Chromosomes	Tandem centromeric repeats
Ch1	CCCTAAAATAGTTTGCTGCTTCTTATGACATTCATTGTTTTGATCAACATAGCAACACGAGAGAAGGTTTATAATGAATCCTGCCA
Ch2	AATTGAAATCATAAATGAAAACCAG
Ch3	ACAACAACATGAGAGAAGGTTTATAATGAATCCTGCCACCCTAAAATCATTTGCTGCTTCTTATGAAATCATTGTTTTGATCGAC
Ch4	GAGGAGTGGTAACCTGAGTATGCAGGTCACCAGTGACCTAAACTATAGGTCACTACCTACTTCACACCTACCTCAAGTCATATCGCCTACCTCAAACCACATGAACCATGTGGTTCCTAACACATGCATCATGTGTTAGTACTTAGTACTAACTAATCATTTAGGCTAAGCTAGAGCTTGGAGTATTAACA
Ch5	TCACCGATGACCTGATCTATAGGTCACTACCTACTTCACACCTACCTCAAGTCATATGACCTACCTCAAACCTCATGAACCATGTGGTTCCTAACACATACATCATGTGTTAGTACTTAGTCCTAATTAATCATTTAGGCTAAGCTAGAACTTGGAGTATTAACAGTTTCTTCCACCAGAACTCGGAGGGTGA
Ch6	GGTTGTGAGCCACTTTTAACCGCGCGGAGTTGATCCCGGGTACAAATTTCCCCAGAAGTATATATGAGGGGGAGGGGTTCGGCGTCTCGGTCCAGGCGCACGGAACGGACCGGCCTCGGGTCTCTTCGGTTGGTCCGGCTCGGTG
Ch7	AAATCATTTGCTGCTTCTTATGAAATCATTGTTTTGATCGACACAGCACCATGAGAGAAGGTTTATAATGAATCCTGTCACCCTAAAATCATTTGCTGCTTCTTATGAAATCATTGTTTTGATCGACACAGCACCATGAGAGAAGGTTTATAATGAATCCTGTCACCCTAAAATCATTTGCTGCTTCTTATGAAATCATTGTTTTGATCGACACAGCACCATGAGAGAAGGTTTATAATGAATCCTGTCACCCTA
Ch8	CGGGTTGTGAGCCACTCTTAACCGCGCGGAGTTGATCCCGGGTACAAATTTCCCCAGAAGTATATATGAGGGGGAGGCGTTCGGCGTCTCGGTCCAGGCGCACGGAACGGACCGGCCTCGGGTCTCGGCTTGTGCTCGGTTGAGG
Ch9	TCTCTCTCTCTCTCTCTCTCTCTCTCTCCCTGCCATCGCATCTCGAGCTGATCAGAGCTCGACCTCGTCTTCTCTCTCTCTCTCTCTCTCTCTCTCTCTCTCTCTGCCATCGCATTTCGAGCTGATCAGAGTTCGACCTCGTCCT

Supplementary figures

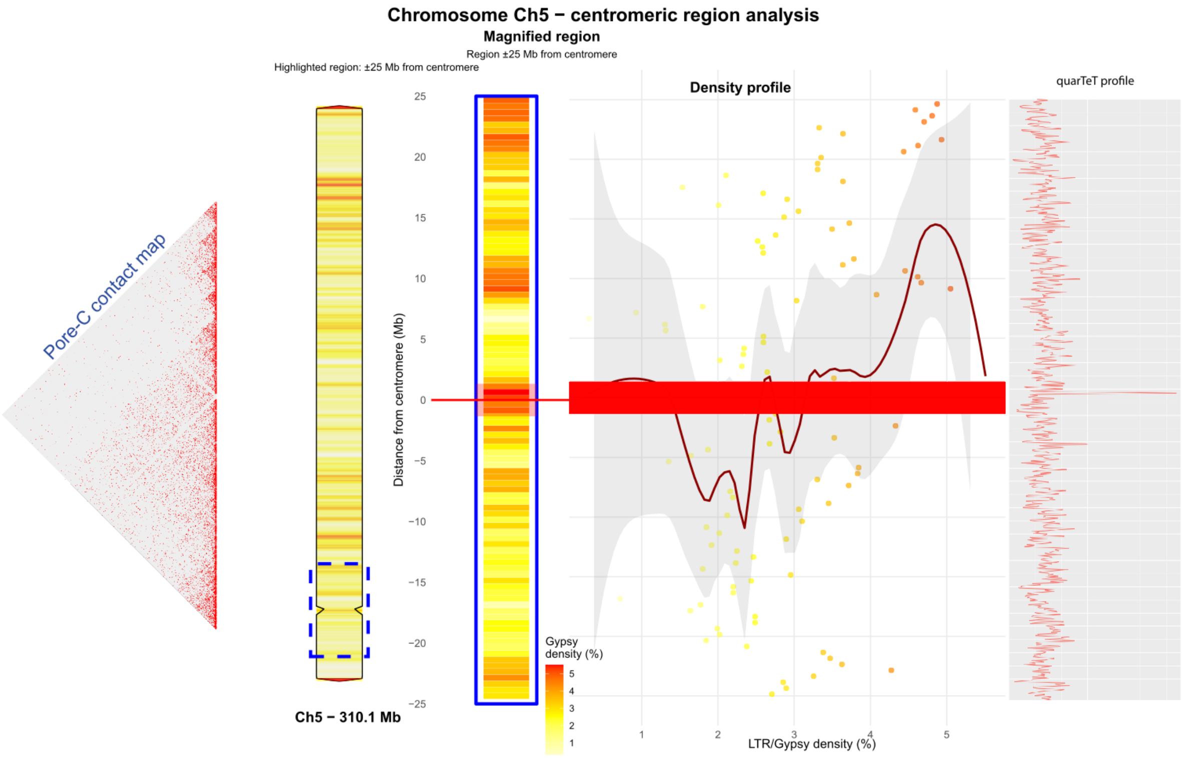

Figure S1. Detailed examination of the predicted centromere of chromosome 5 – Analysis of *Gypsy* retrotransposon distribution and chromatin interactions across chromosome 5. In the density profile, each dot represents a single genomic window, plotting its LTR/Gypsy density (X-axis) against its chromosomal position (Y-axis). The solid dark red line shows the overall trend in density, calculated using a LOESS regression, while the surrounding light grey shaded area indicates the 95% confidence interval for this trend. The thick red horizontal bar marks the position of the centromere across the center of the magnified plots.

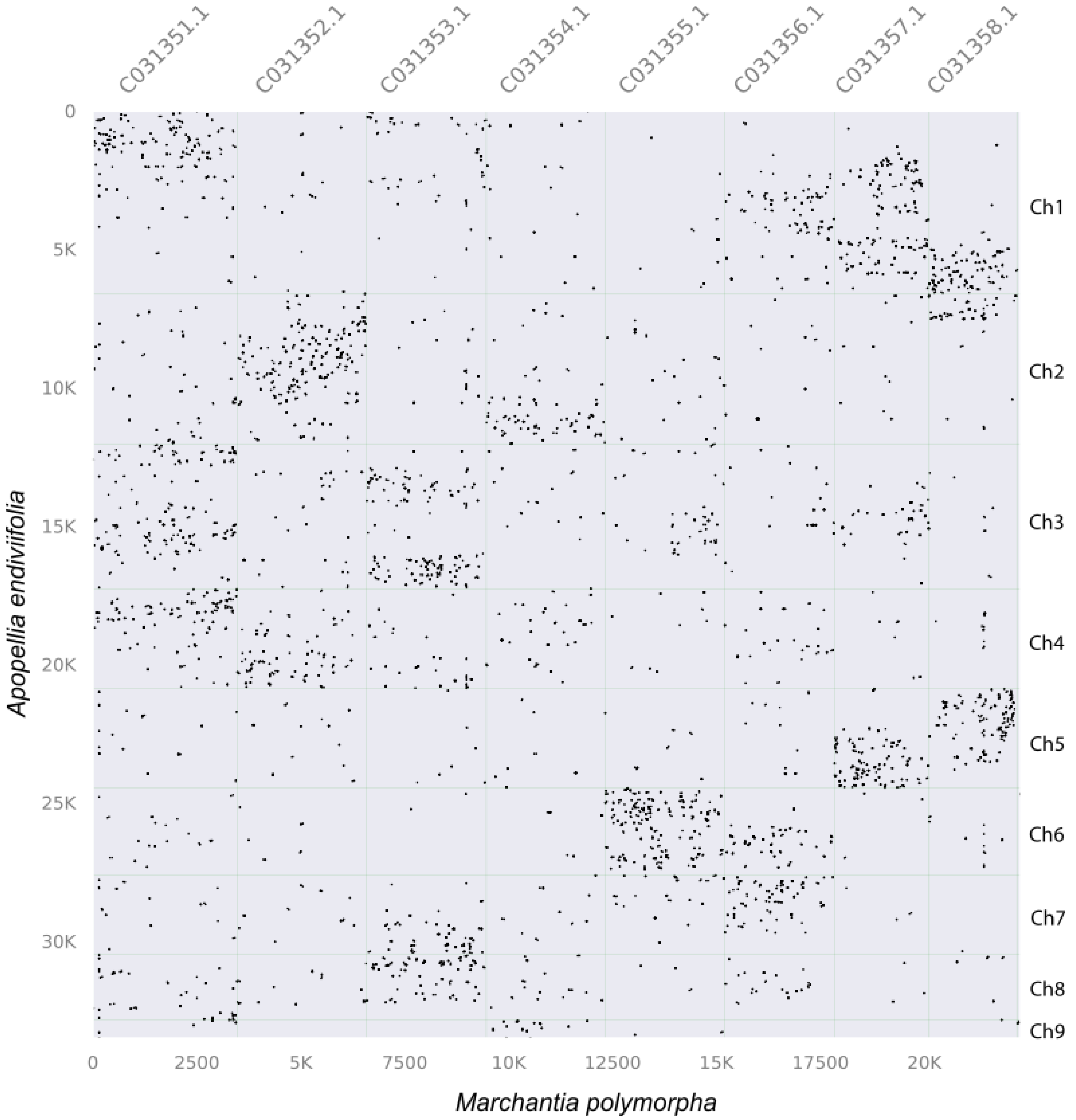

Figure S2. Synteny dot plot comparing the genomes of *M. polymorpha* and *A. endiviifolia*.

Click here to access/download
Supplementary Material
Reviewer reports MINOR.docx

Click here to access/download
Supplementary Material
Supplementary Table S2.xlsx

Personal Cover
Click here to access/download;Personal Cover;Cover letter.docx

Dear Editors of GigaScience,

We are pleased to submit our manuscript entitled "Giant chromosomes of tiny plant - the complete telomere-to-telomere genome assembly of the simple thalloid liverwort *Apopellia endiviifolia* (Jungermanniopsida, Marchantiophyta)" for consideration for publication in GigaScience.

Our study presents the first high-quality chromosome-level genome assembly of the haploid liverwort *A. endiviifolia*. This species is a key model for understanding cryptic diversity, genomic innovation, and the evolutionary mechanisms underlying speciation in liverworts. The comprehensive genomic resources and analyses we offer, encompassing genome assembly and annotation, as well as the detection and characterization of telomeres and centromeres, will be of significant interest to the genomics, evolutionary biology, and plant science communities.

We confirm that our manuscript fully adheres to the policies of GigaScience. All authors have reviewed and approved the final version of the manuscript and consented to its submission to GigaScience and declare that there are no competing interests related to this work. Furthermore, we confirm that the content of this manuscript has not been previously published, nor is it under consideration for publication elsewhere. We are confident that our manuscript is of high quality and a good fit for your journal. Thank you for your consideration.

Sincerely
Joanna Szablińska-Piernik
Jakub Sawicki
