# Supplementary material for: Giant chromosomes of a tiny plant—the complete telomere-to-telomere genome assembly of the simple thalloid liverwort Apopellia endiviifolia (Jungermanniopsida, Marchantiophyta)
Source: Gigascience. 2025 Nov 29;15:giaf145. doi: 10.1093/gigascience/giaf145 (PMC12885004; doi:10.1093/gigascience/giaf145)
Supplement: giaf145_Authors_Response_To_Reviewer_Comments_original_submission [file giaf145_authors_response_to_reviewer_comments_original_submission.pdf]

Author's Response To Reviewer Comments

We thank the Editor and Reviewers for all their valuable comments that helped us improve our manuscript. Below are detailed answers to each question or comment raised in the reviews.

Responses to Editor comments:

1. All web links and URLs should be given a reference number and included in the reference list rather than within the text of the manuscript. Please remove the URLs, cite them as reference and adjust the order of the reference accordingly. If you are using a reference manager then it's much easier for you to fix this.

Answer: All links and URLs have been added to the reference list.

2. In the methods section it would be useful if you add RRID details to some of the software tools and sequencers. These needed to be listed after resources in brackets. If you are citing papers for these resources, the RRID do not replace these, and both should be included. This can be included in the methods section of the paper similar to the RRIDs included here:
DNBSEQ-T7 (RRID:SCR_017981); PacBio Sequel II System (RRID:SCR_017990); PLINK (RRID:SCR_001757)

Answer: RRID details have been added to all software tools and sequencers for which they were found.

Responses to Reviewer #1:
Reviewer #1: Review to the manuscript entitled: "Giant chromosomes of tiny plant - the complete telomere-1 to-telomere genome assembly of the simple thalloid liverwort Apopellia endiviifolia (Jungermanniopsida,Marchantiophyta)"
Overall quality
The manuscript by Joanna Szablińska-Piernik et al. reports the annotated genome of the liverwort Apopellia endiviifolia. The manuscript properly follows the formal requirements of genome reports published in Gigascience. It contains a sufficiently detailed introduction a well-structured result, a method and a short discussion section. The manuscript is relatively easy to read but its text would benefit from some editing and streamlining. Interpretation of the data is aided by figures and tables as well as supplementary files. Assembly and annotation files are provided and available on Gigascience`s ftp site. Figures and tables are informative with some errors that must be corrected prior publishing. The methods section needs also further improvement as some analyses are not sufficiently described in detail. The A. endiviifolia genome is of great importance because of its phylogenetic position (first simple thalloid genome sequenced) as well as its large size which contrasts with currently sequenced liverwort genomes.
Furthermore, A. endiviiifolia has been already used in the past as a model to study cytology, molecular biology, speciation as well as physiology of liverworts. This genome sequence will make it possible to develop this species into a proper model system within liverworts. Therefore, the manuscript provides a great contribution to the field, and the liverwort community will benefit from the availability of the chromosomal-scale assembly of A. endiviifolia.

Answer: We sincerely appreciate your detailed, thorough, and constructive review. We have carefully considered all your comments and suggestions, and have revised the manuscript accordingly to enhance its overall quality.

Nevertheless, the manuscript needs heavy editing to be publishable. My general concerns are the following:
Intro: The intro and the results section refer to liverworts as well as to Apopellia as a basal linage. This term should not be used as lineages are not basal or derived and their position is only relative and depends on the way (top, bottom) the phylogenetic tree was drawn (which is in itself an arbitrary decision). Please, revise these statements and rather refer to the age of the split between Pelliaceae and other liverworts. A similar statement must be revised claiming that liverworts are sister to all land plants as well as that they retained more ancestral characters than vascular plants.

Answer: Thank you for this important clarification regarding the terminology and phylogenetic interpretation. We have revised the Background and Results sections accordingly, avoiding phrases 'basal lineage' and 'sister to all land plants' and a clear statement about 'ancestral traits'.

Methods: for many of the software codes used the version is not reported. This is necessary to make the analyses repeatable. Also, some analyses lack sufficient details that must be further supplemented.

Answer: Thank you for pointing this out. We have now included the specific version numbers for all software tools used throughout the study to ensure full reproducibility of our analyses.

Results: One interesting part of this manuscript is the discovery of centromeres that appear to be different from what has been described for other liverworts sequenced so far. While it is tempting to believe that centromere structure of Apopellia is unique within liverworts, I think the interpretation must mention that centromeres have been only in silico predicted which represents a good but not yet fully verified hypothesis. Furthermore, the plots showing detailed genomic features of the centromeric regions, the tandem and nested putative centromeric repeats are not shown. I do think that this information would help the reader to interpret the data and vet the validity of the predicted centromeres.

Answer: Your comment has been included in the 're-use potential' section. Additionally, tandem centromeric repeats are provided in the Supplementary Table S1.

Detailed comments to the authors:
L1: "a tiny plant" instead of "tiny plant"
L19: it is "dioicous" and not "dioecious", also remove "and" after dioicous. "dioicous" is the term used for haploid plants.
L20: there is no "basal phylogenetic position". You could write "represents the deepest split within Jungemanniopsida" or "in a clade that is sister to all other Jungemanniopsida". Please, revise.
L22: What is "genomic structure", I would rather say "genome evolution" or "genome structure".
L24: write rather "haploid A. endiviifolia isolate"
L26 What does accuracy refer to ?
L27 rather "18 validated Telomeres"
L29 LTR rather than LTRs
L32: Gene density needs a unit: number of genes / 1kb or so. Please, revise.
L34: rather "extensive rearrangements among species"

Answer: All errors and typos mentioned above have been corrected in accordance with the comments.

L41 There is no "early divergent" rephrase it. Also, it would be better to say that they are part of bryophytes, but it forms a monophyletic clade with mosses. This is what the current phylogenetic data suggest.
L43-44: It is not sister to all other land plants. Revise. The best supported phylogenetic hypothesis resolves vascular plants and bryophytes within land plants each as monophyletic lineages. Within bryophytes, liverworts are sister to mosses and this clade is further sister to hornworts.
L46: again, it is not clear whether their features are retained or secondarily reduced. Currently the reduction hypothesis is preferred.

Answer: Your comments have been taken into account and the beginning of the introduction has been edited as follows: 'Liverworts (Marchantiophyta) represent one of the earliest terrestrial plants, with fossil evidence indicating their emergence in the Middle Ordovician period approximately 419–447 million years ago [1]. They occupy a pivotal position in plant evolutionary history as members of the monophyletic bryophyte lineage, which, along with vascular plants, form two distinct monophyletic groups within terrestrial plants. Within bryophytes, liverworts are sister to mosses, forming the Setaphyta clade, which in turn is sister to hornworts. This phylogenetic framework is supported by recent genomic analyses that affirm the monophyly of bryophytes and clarify the evolutionary relationships among early land plants [2]. Liverworts, along with other bryophytes such as mosses and hornworts, exhibit a suite of morphological and physiological features that shed light on plant adaptation to terrestrial environments, although it

remains debated whether these features represent ancestral traits or are the result of secondary reductions, with recent evidence favoring the latter [3]. This group is often characterized by structural simplicity, a predominantly haploid life cycle, and a slow rate of molecular evolution, which corresponds to gradual morphological diversification [4].’

L47-50 I miss the citations referring to the slow evolutionary rates mentioned.

Answer: The citation has been completed: Linde AM, Singh S, Bowman JL, Eklund M, Cronberg N, Lagercrantz U. Genome Evolution in Plants: Complex Thalloid Liverworts (Marchantiopsida). Genome Biol Evol. Oxford University Press; 2023; doi: 10.1093/GBE/EVAD014.

L57 there is no "basal lineage", revise.

Answer: This has been removed.

L84 approximately, does this refer to the mean value?

Answer: Yes, this refers to the average value, 'approximately' has been removed.

L134: In vitro cultures are not "conducted" rather "established".

Answer: This has been corrected.

L140: Explain the meaning of culture sterility of 95%.

Answer: The results of culture sterility were given here to demonstrate the high effectiveness of the sterilization method, which we have used. Only sterile plants were used in the studies.

L169: I think it is protein digestion and not degradation.

Answer: The original ONT protocol (https://nanoporetech.com/document/extraction-method/plant-pore-c) uses the word ‘degradation’ and we agree that it is degradation rather than digestion.

L189 Please, report concentration of proteinase K and RNase used.

Answer: This information has been supplemented: ‘100 µl proteinase K (20 µg/µL) and 3 µl RNase A (100 µg/µl)’.

L180 please report version of minknow used.

Answer: This information has been supplemented: ‘v24.11.10’.

L189: please, explain why two different library preps. were used.

Answer: The entire paragraph on RNA-seq has been edited and described in more detail as follows: Total RNA for short read procedure was extracted from four samples of male A. endiviifolia: land (i) and water (ii) form, antheridia (iii) and surrounding thallus (iv). This extraction was conducted using the RNA Plant Mini Spin Kit (Qiagen) according to the manufacturer’s protocol. Short-read RNA-seq libraries were prepared using two distinct protocols. For RNA extracted from the water and land form of A. endiviifolia, libraries were constructed using the TruSeq Stranded Total RNA Library Prep Kit (Illumina) using the Ribo-Zero rRNA option, whereas for antheridia and surrounding thallus, which amount of extracted RNA was insufficient for TruSeq, libraries were prepared using the QIAseq FX Single Cell RNA Library Kit (Qiagen). All libraries were sequenced on an Illumina NovaSeq 6000 platform (Macrogen, Inc., Seoul, South Korea) in 2 × 150 pair-end mode. Raw reads were deposited in BioProject PRJNA1279829 and BioSample SAMN49506713.

L190: sequencing, how, 150 bp paired-end? Stranded? please, explain.

Answer: This information has been supplemented ‘in 2x150 pair-end mode’.

L215: You say SAMBA gap-closed assembly was…. Please, let us know the parameters used for the SAMBA gap-closing procedure.

Answer: The following information has been added: ‘Samba scaffolders for gap-closing were run with default option with exception of -m parameter (minimum matching length) set to 9000 as recommended for large plant genomes [37].’

L218: citation of dorado is missing.

Answer: The citation has been completed: https://github.com/nanoporetech/dorado.

L223: I would also report results for the embryophyte ODB data set.
Answer: we added embryophyte OBD10 BUSCO results to the Table 1
L233: Could you explain why did you prefer EGAPx over other genome annotation pipelines? For instance, EGAPx only uses a restricted set of plant proteins. Especially bryophyte proteins are missing.

Answer: In our internal testing EGAPx generates more accurate annotation than BREAKER2, which was used in our previous studies [Levins et al. 2025 (Levins J, Paukszto Ł, Krawczyk K, Maździarz M, Arch BC, Cargill DC, et al.. Evolution of sexual systems and regressive evolution in Riccia. New Phytologist. John Wiley & Sons, Ltd; 2025; doi: 10.1111/NPH.20454.), Krawczyk et al. 2025 (Krawczyk K, Szablińska-Piernik J, Paukszto Ł, Maździarz M, Sulima P, Przyborowski JA, et al.. Chromosome-scale telomere to telomere genome assembly of common crystalwort (Riccia sorocarpa Bisch.). Scientific Data 2025 12:1. Nature Publishing Group; 2025; doi: 10.1038/s41597-025-04373-6.)]. Moreover, EGAPx enables the use of a custom protein set (in this study we used a protein set from Marchantia polymorpha (Tak-1/2 v7.1).

L234: Was this a male isolate as the RNAseq used was from a male?

Answer: Yes, all isolates used in this study were from males.

L247: which edta version was used? Some versions have issues, so it is important to report the version.

Answer: This information has been supplemented: ‘v2.2.2.’

L256: For each software please describe the version and options used. If it was run using default options, please, state this.

Answer: The information for each software version and option has been supplemented.

L277 indicate M poly genome version.

Answer: Marchantia polymorpha genome version is ‘Tak-1/2 v7.1’

L278 CHEK GENBANK accession

Answer: Yes, we made a mistake here, the accession numbers have been corrected as follows: ‘The genomic sequences (FASTA) and gene annotation files (GFF3) for M. polymorpha (Tak-1/2 v7.1, GCA_037833965.1) and A. endiviifolia (JBRAUX000000000) were obtained from GenBank.’

L312: gggenomes package version

Answer: This information has been supplemented: ‘v1.0.1’.

L339_341: "differring evolutionayr pressures"? could you explain this a bit more in details?

Answer: Thank you for pointing this out. We agree that the phrase “differing evolutionary pressures” was rather unclear, and we have revised the text to provide a clearer explanation: ‘Additionally, the observed variation in genome size, particularly in the large genomes of some Jungermanniopsida, may reflect lineage-specific differences in genome dynamics, such as variation in transposable element activity, gene duplication, and structural rearrangements.’

L466: Close the bracket.

Answer: This has been corrected.

References: formatting must be checked. Some formatting is incorrect (see L508).

Answer: This has been corrected.

Results
Table 2: reports telomere but the identification of telomeres is not described in the methods. ** shows verification with poreC and quartet what about the others? Have they been identified in another way?

Answer: The identification of telomeres was performed using quartet (RRID:SCR_025258).
‘*’ and ‘**’ explanations have been changed as follows: * - telomeres with a non-canonical ACGCAGC/TGCGTCG motif; ** - centromeres verification based only on quarTeT and Pore-C contact map. Tandem centromeric repeats were provided in the Supplementary Table S1.

Fig5 Do tips labelled with the colored circles represent the centromere localized LTRs?

Answer: Yes, the marked tips represent centromere enriched LTRs. This has already been stated in the caption of the figure.

Fig6 needs some further explanations in the legend. Lines, dots are not clearly explained. Also, meaning of the shaded area and that of the Y axis is not explained.

Answer: Additional explanation has been provided: ‘In the density profile, each dot represents a single genomic window, plotting its LTR/Gypsy density (X-axis) against its chromosomal position (Y-axis). The solid dark red line shows the overall trend in density, calculated using a LOESS regression, while the surrounding light grey shaded area indicates the 95% confidence interval for this trend. The thick red horizontal bar marks the position of the centromere across the center of the magnified plots.’

Fig 8. The legend states that colored line thickness reflects collinearity. I think the ribbons just connect collinear regions, but their thickness has nothing to do with the strengths/extent of collinearity at all. Please, revise it.

Answer: The legend has been revised: ‘Figure 8. Synteny plot of the differences and similarities between the assembly of A. endiviifolia chromosomes and that of H. hutchinsiae. Colored lines connect collinear regions between the species' genomes.’

Fig9. The legend does not state that boxes refer to genome sizes of the species. Also, a scale to the genome sizes is missing. Revise.

Answer: The legend has been revised: ‘Figure 9. Phylogenetic relationships among liverworts determined by conserved single-copy orthologs. Size of orange and blue boxes corresponds to genome size variations in liverwort species. All nodes have the maximum statistical support.’
The genome size scale is shown in the figure. It is the black box representing 1000 Mbp.

FigS1 The legend must be revised and extended to provide more information on which symbol refers to which feature of the genome. Also, it is not clear whether the pore-c contact map spans the entire chromosome or not as it lacks a scale. I would also suggest changing the representation a bit as the figure mainly focuses on the density of Gypsy elements, but it does not show the tandem-nested putative

centromeric repeats. Finally, the gypsy element density appears to be only considerably high on one side of the putative centromere while the other side is not that rich. I think this should be mentioned as this type of centromere identification is ambiguous. This is fine but must be acknowledged.

Answer: The pore-C range is now better explained on Figure S1 and Figure 6 and is no longer misleading. We also provide detailed captions to Gypsy density plot. 'In the density profile, each dot represents a single genomic window, plotting its LTR/Gypsy density (X-axis) against its chromosomal position (Y-axis). The solid dark red line shows the overall trend in density, calculated using a LOESS regression, while the surrounding light grey shaded area indicates the 95% confidence interval for this trend. The thick red horizontal bar marks the position of the centromere across the center of the magnified plots.'

Responses to Reviewer #2:
Reviewer #2: The authors present a telomere-to-telomere (T2T) genome assembly of the liverwort Apopellia endiviifolia using Oxford Nanopore long-read sequencing combined with Pore-C proximity ligation data. The study reports a complete assembly of nine chromosomes with telomeres and centromeres identified, alongside genome annotation and synteny analyses. This work represents a valuable addition to bryophyte genomics, particularly given the scarcity of high-quality T2T assemblies for liverworts.

However, several aspects of the manuscript require substantial clarification and expansion before it can be considered for publication. In its current form, the manuscript reads more like a technical report than a genomic resource paper that provides novel biological insights. Key biological findings are underdeveloped, figures lack clarity, and comparisons with existing T2T plant genomes are minimal. I recommend major revisions.

Answer: We sincerely appreciate your insightful and constructive feedback, which has been instrumental in refining and strengthening our manuscript. Below, we detail the revisions made in response to your comments.

Major Comments:
1. The background section devotes substantial space to discussing speciation mechanisms and RNA editing. However, these topics are not addressed in the results or discussion, leaving the reader with unfulfilled expectations. If these topics are retained in the introduction, relevant analyses or interpretations should be provided in later sections; otherwise, the background should be streamlined.

Answer: Thank you for highlighting this point. We have taken your suggestion into account and consequently removed the section on RNA editing from the Introduction to maintain focus and avoid raising expectations that are not addressed later in the manuscript.

2. The authors report non-canonical telomeric sequences (ACGCAGC and TGCGTCG) on chromosomes 3 and 6, which is an unusual and potentially important finding. However, no biological interpretation or comparison with other bryophytes or plant lineages is provided. This omission weakens the impact of the study. The authors should at least speculate on possible mechanisms or evolutionary significance, or cite similar patterns in other species if known.

Answer: We have added commentary on telomeres in the 're-use potential' section: 'The unusual presence of non-canonical telomeric-like repeats (ACGCAGC and TGCGTCG) on chromosomes 3 and 6 in A. endiviifolia suggests species-specific telomere evolution or chromosomal rearrangements, though their biological significance is unclear. Further comparative and functional studies are needed to understand their origin and role. These findings contribute to the genomic landscape of A. endiviifolia and support future research on plant telomere diversity and evolution. Notably, the centromeres have been predicted solely through in silico analyses, representing a plausible but not yet fully validated hypothesis.'

3. While the authors identify centromeres and note Gypsy/Tat family enrichment, there is minimal discussion of how this compares to other bryophyte or land plant centromeres. For example, how does the 1.9-5 Mbp centromere size compare to Riccia, Marchantia, or Physcomitrium? Are these unusually large? What might this suggest about karyotype or genome evolution? The current treatment is descriptive but

lacks insight.

Answer: This article has been prepared in 'Data Note' format. As required by the publisher, this type of article in GigaScience does not include discussion within its structure. The section on actor instructions includes the following information: ‘Data Notes focus on a particular dataset, and provide detailed methodology on data production, validation, and potential reuse. … Manuscripts containing more detailed biological, medical or technical analyses of data should be submitted as a Research Article. One of the aims of a Data Note is to incentivize and more rapidly release data before subsequent detailed analysis has been carried out.’

4. The synteny analysis between A. endiviifolia and other liverworts is reduced to a brief mention of 'fragmented collinearity.' No quantification of syntenic block sizes, gene retention, or structural rearrangements is provided. Similarly, the phylogenetic tree, though technically sound, is not interpreted in depth. What does this tree reveal about genome size evolution or divergence times? The authors should expand the discussion and contextualize their findings.

Answer: Details of the detected syntenic blocks between A. endiviifolia and H. hutchinsiae chromosomes, including chromosome coordinates and strand orientation, are provided in Supplementary Table S2.

Minor Comments
Background
-"dioecious" should be corrected to "dioicious."

Answer: This has been corrected.

-The term "basal phylogenetic position" is problematic. In phylogenetic trees, clade relationships are dichotomous, and describing a clade as "basal" is misleading.

-The authors state, "They hold a crucial position in plant evolutionary history as sister groups to all other extant land plants." However, the phylogenetic relationships among the three bryophyte lineages and their placement relative to other land plants have been the subject of long-standing debate. Recent genomic analyses tend to support bryophyte monophyly, with liverworts and mosses forming a clade.

Answer: Your comments have been taken into account, the beginning of the introduction has been edited as follows: ‘Liverworts (Marchantiophyta) represent one of the earliest terrestrial plants, with fossil evidence indicating their emergence in the Middle Ordovician period approximately 419–447 million years ago [1]. They occupy a pivotal position in plant evolutionary history as members of the monophyletic bryophyte lineage, which, along with vascular plants, form two distinct monophyletic groups within terrestrial plants. Within bryophytes, liverworts are sister to mosses, forming the Setaphyta clade, which in turn is sister to hornworts. This phylogenetic framework is supported by recent genomic analyses that affirm the monophyly of bryophytes and clarify the evolutionary relationships among early land plants [2]. Liverworts, along with other bryophytes such as mosses and hornworts, exhibit a suite of morphological and physiological features that shed light on plant adaptation to terrestrial environments, although it remains debated whether these features represent ancestral traits or are the result of secondary reductions, with recent evidence favoring the latter [3]. This group is often characterized by structural simplicity, a predominantly haploid life cycle, and a slow rate of molecular evolution, which corresponds to gradual morphological diversification [4].

-The authors write, "Its chloroplast genome exhibits an intermediate level of RNA editing, bridging the gap between non-editing liverworts, such as Marchantia polymorpha, and seed plants." In liverworts, only the complex thalloid lineage lacks RNA editing; most other liverworts possess RNA editing sites in their organellar genomes. Please refer to:
Dong SS, Zhao CX, Zhang SZ, Wu H, Mu WX, Wei T, Li N, Wan T, Liu H, Cui J, Zhu RL, Goffinet B, Liu Y. 2019. The amount of RNA editing sites in liverwort organellar genes is correlated with GC content and nuclear PPR protein diversity. Genome Biology and Evolution 11: 3233-3239.

Answer: The text section regarding RNA editing has been removed.

Material and Methods
-Was a voucher specimen prepared for the sequenced sample? If so, please indicate where it is deposited.

Answer: A voucher specimen was prepared for the sequenced sample. It has been deposited in the Herbarium of University of Warmia and Mazury in Olsztyn (OLS), under the accession number OLS-H2024P004.

-Although the experiments appear to have used axenic culture of the liverwort (please confirm if this is correct), the culture was not initiated from a single spore/shoot. This implies that the material may represent a population of the species. When assembling such sequence reads, how were polymorphisms among the reads handled? Please clarify.

Answer: Yes, the experiments appear to have used axenic culture of the liverwort. And also the culture was initiated from a single fragment of the thallus, that was used for the growth and development of the plant, which was then multiplied in vitro. The plants used in the study were genotypically identical.

-For RNA-seq, the tissue type used for RNA extraction was not specified. Please provide this information.

Answer: This information has been supplemented: 'Total RNA for short read procedure was extracted from four samples of male A. endiviifolia: land (i) and water (ii) form, antheridia (iii) and surrounding thallus (iv). This extraction was conducted using the RNA Plant Mini Spin Kit (Qiagen) according to the manufacturer's protocol.'

-The statement "A custom Python script (analyze_genome_wide_ltr_domains.py) was developed" should be accompanied by information on where this script is deposited.

-Similarly, for "Custom Python scripts were used to extract the protein sequences of the single-copy orthologs shared across all 14 species", please clarify what these scripts are and where they are deposited.

Answer: All custom scripts used have been provided with citations indicating where they were deposited. [50 - GitHub - kubek78/PhylogenomicsME: Phylogenomics Made Easy. https://github.com/kubek78/PhylogenomicsME/tree/main]

Results
-The manuscript mentions six remaining gaps but does not indicate whether these are intergenic, pericentromeric, or subtelomeric. Given the emphasis on T2T quality, this should be specified.

Answer: We clarified that these were intergenic regions.

-The reported 68.5 Gbp of Nanopore raw data would theoretically yield only ~20× average depth, and the reads have a mean quality score of 19. This raises concerns about sequencing accuracy; in such cases, ~100× coverage with WGS short reads would be advisable to improve assembly accuracy.

Answer: Thank you for pointing it out. We provide SRA accession number for the second nanopore library we used for polishing and final coverage calculation (the value was calculated using both libraries) but the SRR35424238 wasn't used in initial assembly steps, due to lower N50 of raw reads.

Figures and other:
-The manuscript alternates between "Helitron" and "Helintron" in the text and figure captions. Please ensure consistent and correct use of TE family names.

Answer: Thank you for pointing that out. It was a typo. The correct name is 'Helitron'.

-Several key figures (e.g., dot plots, Circos plots) are difficult to interpret due to low resolution and unclear labeling. Axis labels, legends, and color schemes should be revised for clarity and readability.

Answer: We acknowledge that the current figures provided are of lower resolution for the initial review. We will prepare and submit higher-resolution versions of the key figures to enhance clarity and readability during the production stage.

-Data availability is incomplete. Only a submission ID (SUB15403958) is provided for the genome assembly. Please include complete accession numbers for raw data, assemblies, and annotations (e.g., BioProject, BioSample, GCA/GCF IDs) before publication.

Answer: Information has been added: For A. endiviifolia genome assembly 'The assembled genome and gene annotation can be found in the NCBI assembly with submission number JBRAUX000000000.' For RNA-seq 'The raw reads were deposited in BioProject PRJNA1279829 and BioSample SAMN49506713.' For M. polymorpha 'GCA_037833965.1' and for H. hutchinsiae 'GCA_965112325.1'.

Language and phrasing issues:
-Some phrases are awkward or unclear. For example, in the Findings section: "gene density varied between a value 5.52 and 9.78" should be revised to "gene density ranged from 5.52 to 9.78 genes per xx kbp." Similar issues occur in lines 383-384. A careful language edit throughout the manuscript would improve readability.

Answer: This has been corrected: '5.52 and 9.78 genes per 500 kbp.'

Cookie Preference Center

We use cookies which are necessary to make our site work. We may also use additional cookies to analyze, improve and personalize our content and your digital experience. For more information, see our Cookie Policy.

You may choose not to allow some types of cookies. However, blocking some types may impact your experience of our site and the services we are able to offer. See the different category headings below to find out more or change your settings.

Allow all

Manage Consent Preferences

Strictly Necessary Cookies

Always active

These cookies are necessary for the website to function and cannot be switched off in our systems. They are usually only set in response to actions made by you which amount to a request for services, such as setting your privacy preferences, logging in or filling in forms. You can set your browser to block or alert you about these cookies, but some parts of the site will not then work.

Functional Cookies

These cookies enable the website to provide enhanced functionality and personalisation. They may be set by us or by third party providers whose services we have added to our pages. If you do not allow these cookies then some or all of these services may not function properly.

Performance Cookies

These cookies allow us to count visits and traffic sources so we can measure and improve the performance of our site. They help us to know which pages are the most and least popular and see how visitors move around the site.

Targeting Cookies

These cookies may be set through our site by our advertising partners. They may be used by those companies to build a profile of your interests and show you relevant adverts on other sites. If you do not allow these cookies, you will experience less targeted advertising.

Cookie List

Clear

Apply Cancel

Consent Leg.Interest

Confirm my choices

Powered by onetrus
