# Supplementary material for: Giant chromosomes of a tiny plant—the complete telomere-to-telomere genome assembly of the simple thalloid liverwort Apopellia endiviifolia (Jungermanniopsida, Marchantiophyta)
Source: Gigascience. 2025 Nov 29;15:giaf145. doi: 10.1093/gigascience/giaf145 (PMC12885004; doi:10.1093/gigascience/giaf145)
Supplement: giaf145_Authors_Response_To_Reviewer_Comments_Revision_1 [file giaf145_authors_response_to_reviewer_comments_revision_1.pdf]

Author's Response To Reviewer Comments

We would like to thank the Reviewers for further valuable suggestions on how to improve the manuscript. Below are answers to each comment.

Reviewer reports:
Reviewer #1: Overall, the authors satisfactorily addressed most of my major concerns. However, I still feel that the current version of the manuscript needs a second round of revision, as it contains awkward and difficult-to-understand sentences. My detailed comments are provided below.

L20 Please, revise: «that is sister to all other» rather than "that is a sister to all other".
L24 Please, revise: "of a haploid" and not "of haploid".
L27 Please rather write "18 validated". But I am not sure that the ms included validation. Centromeres were not cytogenetically validated, only using bioinformatic methods. I would rather say, 18 telomeres.
L29 Please, revise: write "57%" instead of "57".
L48 Please, revise: instead "framework" use "relationship" or "backbone".
L49-53 Please, revise: What is this sentence want to say? It is difficult to follow.
L79 Please, revise: rather "evolution of genomic structure" than "structural genomic evolution".
L85 rather "while liverwort genomes" than "and liverwqort genomes".
L93 Please, revise: awkward sentence….revise for instance "Although liverwort genome sizes are less variable than those of Angiosperms"….
L93-96 Please, revise: The sentence is awkwardly formulated and must be revised.
L99 Please, add a publication referring to the statement of rare occurrence of TE bursts.
L99 I would start the second sentence with "Nevertheless/However a comparative….." because it challenges the statement made in the previous sentence.
L105-109 I understand what you want to say but the sentence is overly complicated. Please, revise it.
L111 Instead of "in the field of bryophyte research" write "in bryophytes".
L116 What is a multi-method study? Don't use this word.
L141 Delete "a" in "under a 16h……".
L193 I would rather write "For antheridia and surrounding thallus, the amount of extracted RNA was insufficient for TruSeq, libraries were prepared using the QIAseq FX Single Cell RNA Library Kit (Qiagen)".
L361-362 Awkward sentence, please, revise it.
L385 I would delete this interpretation "reflecting the relatively limited number of subcellular locations or structures annotated within the genome". It makes not too much sense to interpret why one ontology had more or less unique GO terms as this result mainly depends on the extent of homology between the annotated genome and the homologous gene set from which the GO terms were transferred.
L392 57% and not 57.
Fig9 caption: Revise sentence "Size of orange and blue boxes corresponds to genome size variations in liverwort species" for instance "Size of orange and blue boxes is proportional to the genome size of the species ".

Answer: All errors and awkward sentences mentioned above have been corrected in accordance with the comments, also the citation has been completed.
Reviewer #2: The authors have provided generally thorough and constructive replies to my major and minor comments, and I appreciate the efforts made to improve the manuscript. While most issues were adequately addressed, the synteny and centromere size comparisons still feel somewhat underdeveloped. Even within the constraints of a Data Note, providing a brief numerical or comparative statement (e.g., relative size ranges across liverwort genomes) would make this section more informative to the reader.

Answer: In response to the review, we have added the following information:
- regarding centromeres: "Compared to A. endiviifolia, Marchantia polymorpha centromeres are similarly structured with abundant retroelements and are typically centrally located, ranging from about 176 to 604 Kbp [63]. Physcomitrium patens centromeres share this organization but tend to be smaller, varying from 64 to 221 Kbp, likely reflecting its more compact genome [25]. These differences highlight centromere size

and composition diversity within bryophytes and demonstrate the value of long-read sequencing for resolving complex genomic regions."
- regarding genome synteny analysis: "A total of 429 syntenic blocks spanning approximately 4.8 Mb were detected, with a median size of about 5.2 kb and a mean size of 11.2 kb, indicating small, fragmented conserved regions. These blocks were evenly distributed in terms of strand orientation, with 201 blocks on the positive strand and 228 on the negative strand." ….. "Structural variation analysis revealed numerous chromosomal breakpoints across chromosomes, predominantly in chromosome 1 (54 translocation and 18 inversion breakpoints), while chromosome 9 showed no breakpoints, consistent with its slightly more conserved relationship."
Supplementary Table S3 has been added.

Editorial Manager®

Cookie Preference Center

We use cookies which are necessary to make our site work. We may also use additional cookies to analyze, improve and personalize our content and your digital experience. For more information, see our Cookie Policy.

You may choose not to allow some types of cookies. However, blocking some types may impact your experience of our site and the services we are able to offer. See the different category headings below to find out more or change your settings.

Allow all

Manage Consent Preferences

Strictly Necessary Cookies

Always active

These cookies are necessary for the website to function and cannot be switched off in our systems. They are usually only set in response to actions made by you which amount to a request for services, such as setting your privacy preferences, logging in or filling in forms. You can set your browser to block or alert you about these cookies, but some parts of the site will not then work.

Functional Cookies

These cookies enable the website to provide enhanced functionality and personalisation. They may be set by us or by third party providers whose services we have added to our pages. If you do not allow these cookies then some or all of these services may not function properly.

Performance Cookies

These cookies allow us to count visits and traffic sources so we can measure and improve the performance of our site. They help us to know which pages are the most and least popular and see how visitors move around the site.

Targeting Cookies

These cookies may be set through our site by our advertising partners. They may be used by those companies to build a profile of your interests and show you relevant adverts on other sites. If you do not allow these cookies, you will experience less targeted advertising.

Cookie List

Clear

Apply Cancel

Consent Leg.Interest

Confirm my choices

Powered by onetrus
