# Supplementary material for: Giant chromosomes of a tiny plant—the complete telomere-to-telomere genome assembly of the simple thalloid liverwort Apopellia endiviifolia (Jungermanniopsida, Marchantiophyta)
Source: Gigascience. 2025 Nov 29;15:giaf145. doi: 10.1093/gigascience/giaf145 (PMC12885004; doi:10.1093/gigascience/giaf145)
Supplement: giaf145_Reviewer_1_Report_Original_Submission — Péter Szövényi -- 8/7/2025 [file giaf145_reviewer_1_report_original_submission.pdf]

Reviewer Report

Title: Giant chromosomes of a tiny plant - the complete telomere-to-telomere genome assembly of the simple thalloid liverwort Apopellia endiviifolia (Jungermanniopsida, Marchantiophyta)

Version: Original Submission **Date:** 8/7/2025

Reviewer name: PÃ©ter SzÃ¶vÃ©nyi

Reviewer Comments to Author:

Review to the manuscript entitled: "Giant chromosomes of tiny plant - the complete telomere-1 to-telomere genome assembly of the simple thalloid liverwort Apopellia endiviifolia (Jungermanniopsida,Marchantiophyta)"Overall qualityThe manuscript by Joanna SzabliÅ„ska-Piernik et alReports the annotated genome of the liverwort Apopellia endiviifolia. The manuscript properly follows the formal requirements of genome reports published in Gigascience. It contains a sufficiently detailed introduction a well-structured result, a method and a short discussion section. The manuscript is relatively easy to read but its text would benefit from some editing and streamlining. Interpretation of the data is aided by figures and tables as well as supplementary files. Assembly and annotation files are provided and available on Gigascience`s ftp site. Figures and tables are informative with some errors that must be corrected prior publishing. The methods section needs also further improvement as some analyses are not sufficiently described in detail. The A. endiviifolia genome is of great importance because of its phylogenetic position (first simple thalloid genome sequenced) as well as its large size which contrasts with currently sequenced liverwort genomes. Furthermore, A. endiviiifolia has been already used in the past as a model to study cytology, molecular biology, speciation as well as physiology of liverworts. This genome sequence will make it possible to develop this species into a proper model system within liverworts. Therefore, the manuscript provides a great contribution to the field, and the liverwort community will benefit from the availability of the chromosomal-scale assembly of A. endiviifolia.Nevertheless, the manuscript needs heavy editing to be publishable. My general concerns are the following:Intro: The intro and the results section refer to liverworts as well as to Apopellia as a basal linage. This term should not be used as lineages are not basal or derived and their position is only relative and depends on the way (top, bottom) the phylogenetic tree was drawn (which is in itself an arbitrary decision). Please, revise these statements and rather refer to the age of the split between Pelliaceae and other liverworts. A similar statement must be revised claiming that liverworts are sister to all land plants as well as that they retained more ancestral characters than vascular plants.Methods: for many of the software codes used the version is not reported. This is necessary to make the analyses repeatable. Also, some analyses lack sufficient details that must be further supplemented.Results: One interesting part of this manuscript is the discovery of centromeres that appear to be different from what has been described for other liverworts sequenced so far. While it is tempting to believe that centromere structure of Apopellia is unique within liverworts, I think the interpretation must mention that centromeres have been only in silico predicted which represents a good but not yet fully verified hypothesis. Furthermore, the plots showing detailed genomic features of the centromeric regions, the tandem and nested putative centromeric repeats are not shown. I do think that this information would

help the reader to interpret the data and vet the validity of the predicted centromeres.Detailed comments to the authors:L1: "a tiny plant" instead of "tiny plant"L19: it is "dioicous" and not "dioecious", also remove "and" after dioicous. "dioicous" is the term used for haploid plants.L20: there is no "basal phylogenetic position". You could write "represents the deepest split within Jungemanniopsida" or "in a clade that is sister to all other Jungemanniopsida". Please, revise.L22: What is "genomic structure", I would rather say "genome evolution" or "genome structure".L24: write rather "haploid A. endiviifolia isolate"L26 What does accuracy refer to ?L27 rather "18 validated Telomeres"L29 LTR rather than LTRsL32: Gene density needs a unit: number of genes / 1kb or so. Please, revise.L34: rather "extensive rearrangements among species"L41 There is no "early divergent" rephrase it. Also, it would be better to say that they are part of bryophytes, but it forms a monophyletic clade with mosses. This is what the current phylogenetic data suggest.L43-44: It is not sister to all other land plants. Revise. The best supported phylogenetic hypothesis resolves vascular plants and bryophytes within land plants each as monophyletic lineages. Within bryophytes, liverworts are sister to mosses and this clade is further sister to hornworts.L46: again, it is not clear whether their features are retained or secondarily reduced. Currently the reduction hypothesis is preferred.L47-50 I miss the citations referring to the slow evolutionary rates mentioned.L57 there is no "basal lineage", revise.L84 approximately, does this refer to the mean value?L134: In vitro cultures are not "conducted" rather "established".L140: Explain the meaning of culture sterility of 95%.L169: I think it is protein digestion and not degradation.L189 Please, report concentration of proteinase K and RNase used.L180 please report version of minknow used.L189: please, explain why two different library preps. were used.L190: sequencing, how, 150 bp paired-end? Stranded? please, explain.L215: You say SAMBA gap-closed assembly was…. Please, let us know the parameters used for the SAMBA gap-closing procedure.L218: citation of dorado is missing.L223: I would also report results for the embryophyte ODB data set.L233: Could you explain why did you prefer EGAPx over other genome annotation pipelines? For instance, EGAPx only uses a restricted set of plant proteins. Especially bryophyte proteins are missing.L234: Was this a male isolate as the RNAseq used was from a male?L247: which edta version was used? Some versions have issues, so it is important to report the version.L256: For each software please describe the version and options used. If it was run using default options, please, state this.L277 indicate M poly genome version.L278 CHEK GENBANK accessionL312: gggenomes package version.L339_341: "differring evolutionayr pressures"? could you explain this a bit more in details?L466: Close the bracket.References: formatting must be checked. Some formatting is incorrect (see L508).ResultsTable 2: reports telomere but the identification of telomeres is not described in the methods. ** shows verification with poreC and quartet what about the others? Have they been identified in another way?Fig5 Do tips labelled with the colored circles represent the centromere localized LTRs?Fig6 needs some further explanations in the legend. Lines, dots are not clearly explained. Also, meaning of the shaded area and that of the Y axis is not explained.Fig 8. The legend states that colored line thickness reflects collinearity. I think the ribbons just connect collinear regions, but their thickness has nothing to do with the strengths/extent of collinearity at all. Please, revise it.Fig9. The legend does not state that boxes refer to genome sizes of the species. Also, a scale to the genome sizes is missing. Revise.FigS1 The legend must be revised and extended to provide more information on which symbol refers to which feature of the genome. Also, it is not clear whether the pore-c contact map spans the entire chromosome or not as it lacks a scale. I would also suggest changing the representation a bit as the figure mainly focuses on the density of Gypsy elements, but it

does not show the tandem-nested putative centromeric repeats. Finally, the gypsy element density appears to be only considerably high on one side of the putative centromere while the other side is not that rich. I think this should be mentioned as this type of centromere identification is ambiguous. This is fine but must be acknowledged.

Level of Interest

Please indicate how interesting you found the manuscript: Choose an item.

Quality of Written English

Please indicate the quality of language in the manuscript: Choose an item.

Declaration of Competing Interests

Please complete a declaration of competing interests, considering the following questions:

- Have you in the past five years received reimbursements, fees, funding, or salary from an organisation that may in any way gain or lose financially from the publication of this manuscript, either now or in the future?
- Do you hold any stocks or shares in an organisation that may in any way gain or lose financially from the publication of this manuscript, either now or in the future?
- Do you hold or are you currently applying for any patents relating to the content of the manuscript?
- Have you received reimbursements, fees, funding, or salary from an organization that holds or has applied for patents relating to the content of the manuscript?
- Do you have any other financial competing interests?
- Do you have any non-financial competing interests in relation to this paper?

If you can answer no to all of the above, write 'I declare that I have no competing interests' below. If your reply is yes to any, please give details below.

I declare that I have no competing interest

I agree to the open peer review policy of the journal. I understand that my name will be included on my report to the authors and, if the manuscript is accepted for publication, my named report including any attachments I upload will be posted on the website along with the authors' responses. I agree for my report to be made available under an Open Access Creative Commons CC-BY license (http://creativecommons.org/licenses/by/4.0/). I understand that any comments which I do not wish to be included in my named report can be included as confidential comments to the editors, which will not be published.

Choose an item.

To further support our reviewers, we have joined with Publons, where you can gain additional credit to further highlight your hard work (see: https://publons.com/journal/530/gigascience). On publication of this paper, your review will be automatically added to Publons, you can then choose whether or not to claim your Publons credit. I understand this statement.

Yes Choose an item.
