# Supplementary material for: Giant chromosomes of a tiny plant—the complete telomere-to-telomere genome assembly of the simple thalloid liverwort Apopellia endiviifolia (Jungermanniopsida, Marchantiophyta)
Source: Gigascience. 2025 Nov 29;15:giaf145. doi: 10.1093/gigascience/giaf145 (PMC12885004; doi:10.1093/gigascience/giaf145)
Supplement: giaf145_Reviewer_1_Report_Revision_1 — Péter Szövényi -- 11/6/2025 [file giaf145_reviewer_1_report_revision_1.pdf]

Reviewer Report

Title: Giant chromosomes of a tiny plant - the complete telomere-to-telomere genome assembly of the simple thalloid liverwort Apopellia endiviifolia (Jungermanniopsida, Marchantiophyta)

Version: Revision 1 **Date:** 11/6/2025

Reviewer name: PÃ©ter SzÃ¶vÃ©nyi

Reviewer Comments to Author:

Overall, the authors satisfactorily addressed most of my major concerns. However, I still feel that the current version of the manuscript needs a second round of revision, as it contains awkward and difficult-to-understand sentences. My detailed comments are provided below.L20 Please, revise: Â«that is sister to all otherÂ» rather than "that is a sister to all other".L24 Please, revise: "of a haploid" and not "of haploid".L27 Please rather write "18 validated". But I am not sure that the ms included validation. Centromeres were not cytogenetically validated, only using bioinformatic methods. I would rather say, 18 telomeres.L29 Please, revise: write "57%" instead of "57".L48 Please, revise: instead "framework" use "relationship" or "backbone".L49-53 Please, revise: What is this sentence want to say? It is difficult to follow.L79 Please, revise: rather "evolution of genomic structure" than "structural genomic evolution".L85 rather "while liverwort genomes" than "and liverwqort genomes".L93 Please, revise: awkward sentence….revise for instance "Although liverwort genome sizes are less variable than those of Angiosperms"….L93-96 Please, revise: The sentence is awkwardly formulated and must be revised.L99 Please, add a publication referring to the statement of rare occurrence of TE bursts.L99 I would start the second sentence with "Nevertheless/However a comparative….." because it challenges the statement made in the previous sentence.L105-109 I understand what you want to say but the sentence is overly complicated. Please, revise it.L111 Instead of "in the field of bryophyte research" write "in bryophytes".L116 What is a multi-method study? Don't use this word.L141 Delete "a" in "under a 16h……".L193 I would rather write "For antheridia and surrounding thallus, the amount of extracted RNA was insufficient for TruSeq, libraries were prepared using the QIAseq FX Single Cell RNA Library Kit (Qiagen)".L361-362 Awkward sentence, please, revise it.L385 I would delete this interpretation "reflecting the relatively limited number of subcellular locations or structures annotated within the genome". It makes not too much sense to interpret why one ontology had more or less unique GO terms as this result mainly depends on the extent of homology between the annotated genome and the homologous gene set from which the GO terms were transferred.L392 57% and not 57.Fig9 caption: Revise sentence "Size of orange and blue boxes corresponds to genome size variations in liverwort species" for instance "Size of orange and blue boxes is proportional to the genome size of the species ".

Level of Interest

Please indicate how interesting you found the manuscript: Choose an item.

Quality of Written English

Please indicate the quality of language in the manuscript: Choose an item.

Declaration of Competing Interests

Please complete a declaration of competing interests, considering the following questions:

- Have you in the past five years received reimbursements, fees, funding, or salary from an organisation that may in any way gain or lose financially from the publication of this manuscript, either now or in the future?
- Do you hold any stocks or shares in an organisation that may in any way gain or lose financially from the publication of this manuscript, either now or in the future?
- Do you hold or are you currently applying for any patents relating to the content of the manuscript?
- Have you received reimbursements, fees, funding, or salary from an organization that holds or has applied for patents relating to the content of the manuscript?
- Do you have any other financial competing interests?
- Do you have any non-financial competing interests in relation to this paper?

If you can answer no to all of the above, write 'I declare that I have no competing interests' below. If your reply is yes to any, please give details below.

I declare that I have no competing interests

I agree to the open peer review policy of the journal. I understand that my name will be included on my report to the authors and, if the manuscript is accepted for publication, my named report including any attachments I upload will be posted on the website along with the authors' responses. I agree for my report to be made available under an Open Access Creative Commons CC-BY license (http://creativecommons.org/licenses/by/4.0/). I understand that any comments which I do not wish to be included in my named report can be included as confidential comments to the editors, which will not be published.

Choose an item.

To further support our reviewers, we have joined with Publons, where you can gain additional credit to further highlight your hard work (see: https://publons.com/journal/530/gigascience). On publication of this paper, your review will be automatically added to Publons, you can then choose whether or not to claim your Publons credit. I understand this statement.

Yes Choose an item.
