# Supplementary material for: Giant chromosomes of a tiny plant—the complete telomere-to-telomere genome assembly of the simple thalloid liverwort Apopellia endiviifolia (Jungermanniopsida, Marchantiophyta)
Source: Gigascience. 2025 Nov 29;15:giaf145. doi: 10.1093/gigascience/giaf145 (PMC12885004; doi:10.1093/gigascience/giaf145)
Supplement: giaf145_Reviewer_2_Report_Original_Submission — Yang Liu -- 8/10/2025 [file giaf145_reviewer_2_report_original_submission.pdf]

Reviewer Report

Title: Giant chromosomes of a tiny plant - the complete telomere-to-telomere genome assembly of the simple thalloid liverwort Apopellia endiviifolia (Jungermanniopsida, Marchantiophyta)

Version: Original Submission **Date:** 8/10/2025

Reviewer name: Yang Liu

Reviewer Comments to Author:

The authors present a telomere-to-telomere (T2T) genome assembly of the liverwort Apopellia endiviifolia using Oxford Nanopore long-read sequencing combined with Pore-C proximity ligation data. The study reports a complete assembly of nine chromosomes with telomeres and centromeres identified, alongside genome annotation and synteny analyses. This work represents a valuable addition to bryophyte genomics, particularly given the scarcity of high-quality T2T assemblies for liverworts.However, several aspects of the manuscript require substantial clarification and expansion before it can be considered for publication. In its current form, the manuscript reads more like a technical report than a genomic resource paper that provides novel biological insights. Key biological findings are underdeveloped, figures lack clarity, and comparisons with existing T2T plant genomes are minimal. I recommend major revisions.Major Comments:1. The background section devotes substantial space to discussing speciation mechanisms and RNA editing. However, these topics are not addressed in the results or discussion, leaving the reader with unfulfilled expectations. If these topics are retained in the introduction, relevant analyses or interpretations should be provided in later sections; otherwise, the background should be streamlined.2. The authors report non-canonical telomeric sequences (ACGCAGC and TGCGTCG) on chromosomes 3 and 6, which is an unusual and potentially important finding. However, no biological interpretation or comparison with other bryophytes or plant lineages is provided. This omission weakens the impact of the study. The authors should at least speculate on possible mechanisms or evolutionary significance, or cite similar patterns in other species if known.3. While the authors identify centromeres and note Gypsy/Tat family enrichment, there is minimal discussion of how this compares to other bryophyte or land plant centromeres. For example, how does the 1.9-5 Mbp centromere size compare to Riccia, Marchantia, or Physcomitrium? Are these unusually large? What might this suggest about karyotype or genome evolution? The current treatment is descriptive but lacks insight.4. The synteny analysis between A. endiviifolia and other liverworts is reduced to a brief mention of 'fragmented collinearity.' No quantification of syntenic block sizes, gene retention, or structural rearrangements is provided. Similarly, the phylogenetic tree, though technically sound, is not interpreted in depth. What does this tree reveal about genome size evolution or divergence times? The authors should expand the discussion and contextualize their findings.Minor CommentsBackground-"dioecious" should be corrected to "dioicious."-The term "basal phylogenetic position" is problematic. In phylogenetic trees, clade relationships are dichotomous, and describing a clade as "basal" is misleading.-The authors state, "They hold a crucial position in plant evolutionary history as sister groups to all other extant land plants." However, the phylogenetic relationships among the three bryophyte lineages and their placement relative to other land plants have been the subject of

long-standing debate. Recent genomic analyses tend to support bryophyte monophyly, with liverworts and mosses forming a clade.-The authors write, "Its chloroplast genome exhibits an intermediate level of RNA editing, bridging the gap between non-editing liverworts, such as Marchantia polymorpha, and seed plants." In liverworts, only the complex thalloid lineage lacks RNA editing; most other liverworts possess RNA editing sites in their organellar genomes. Please refer to:Dong SS, Zhao CX, Zhang SZ, Wu H, Mu WX, Wei T, Li N, Wan T, Liu H, Cui J, Zhu RL, Goffinet B, Liu Y. 2019. The amount of RNA editing sites in liverwort organellar genes is correlated with GC content and nuclear PPR protein diversity. Genome Biology and Evolution 11: 3233-3239.Material and Methods-Was a voucher specimen prepared for the sequenced sample? If so, please indicate where it is deposited.-Although the experiments appear to have used axenic culture of the liverwort (please confirm if this is correct), the culture was not initiated from a single spore/shoot. This implies that the material may represent a population of the species. When assembling such sequence reads, how were polymorphisms among the reads handled? Please clarify.-For RNA-seq, the tissue type used for RNA extraction was not specified. Please provide this information.-The statement "A custom Python script (analyze_genome_wide_ltr_domains.py) was developed" should be accompanied by information on where this script is deposited.-Similarly, for "Custom Python scripts were used to extract the protein sequences of the single-copy orthologs shared across all 14 species", please clarify what these scripts are and where they are deposited.Results-The manuscript mentions six remaining gaps but does not indicate whether these are intergenic, pericentromeric, or subtelomeric. Given the emphasis on T2T quality, this should be specified.-The reported 68.5 Gbp of Nanopore raw data would theoretically yield only ~20Ã— average depth, and the reads have a mean quality score of 19. This raises concerns about sequencing accuracy; in such cases, ~100Ã— coverage with WGS short reads would be advisable to improve assembly accuracy.Figures and other:-The manuscript alternates between "Helitron" and "Helintron" in the text and figure captions. Please ensure consistent and correct use of TE family names.-Several key figures (e.g., dot plots, Circos plots) are difficult to interpret due to low resolution and unclear labeling. Axis labels, legends, and color schemes should be revised for clarity and readability.-Data availability is incomplete. Only a submission ID (SUB15403958) is provided for the genome assembly. Please include complete accession numbers for raw data, assemblies, and annotations (e.g., BioProject, BioSample, GCA/GCF IDs) before publication.Language and phrasing issues:-Some phrases are awkward or unclear. For example, in the Findings section: "gene density varied between a value 5.52 and 9.78" should be revised to "gene density ranged from 5.52 to 9.78 genes per xx kbp." Similar issues occur in lines 383-384. A careful language edit throughout the manuscript would improve readability.

Level of Interest

Please indicate how interesting you found the manuscript: Choose an item.

Quality of Written English

Please indicate the quality of language in the manuscript: Choose an item.

Declaration of Competing Interests

Please complete a declaration of competing interests, considering the following questions:

- Have you in the past five years received reimbursements, fees, funding, or salary from an organisation that may in any way gain or lose financially from the publication of this manuscript, either now or in the future?
- Do you hold any stocks or shares in an organisation that may in any way gain or lose financially from the publication of this manuscript, either now or in the future?
- Do you hold or are you currently applying for any patents relating to the content of the manuscript?
- Have you received reimbursements, fees, funding, or salary from an organization that holds or has applied for patents relating to the content of the manuscript?
- Do you have any other financial competing interests?
- Do you have any non-financial competing interests in relation to this paper?

If you can answer no to all of the above, write 'I declare that I have no competing interests' below. If your reply is yes to any, please give details below.

I declare that I have no competing interests.

I agree to the open peer review policy of the journal. I understand that my name will be included on my report to the authors and, if the manuscript is accepted for publication, my named report including any attachments I upload will be posted on the website along with the authors' responses. I agree for my report to be made available under an Open Access Creative Commons CC-BY license (http://creativecommons.org/licenses/by/4.0/). I understand that any comments which I do not wish to be included in my named report can be included as confidential comments to the editors, which will not be published.

Choose an item.

To further support our reviewers, we have joined with Publons, where you can gain additional credit to further highlight your hard work (see: https://publons.com/journal/530/gigascience). On publication of this paper, your review will be automatically added to Publons, you can then choose whether or not to claim your Publons credit. I understand this statement.

Yes Choose an item.
